# 1,6-*epi*-Cyclophellitol Cyclosulfamidate Is a Bona Fide Lysosomal α-Glucosidase Stabilizer for the Treatment of Pompe Disease

**DOI:** 10.1021/jacs.2c05666

**Published:** 2022-08-02

**Authors:** Ken Kok, Chi-Lin Kuo, Rebecca E. Katzy, Lindsey T. Lelieveld, Liang Wu, Véronique Roig-Zamboni, Gijsbert A. van der Marel, Jeroen D. C. Codée, Gerlind Sulzenbacher, Gideon J. Davies, Herman S. Overkleeft, Johannes M. F. G. Aerts, Marta Artola

**Affiliations:** † Department of Medical Biochemistry, Leiden Institute of Chemistry, Leiden University, Einsteinweg 55, Leiden 2333 CC, The Netherlands; ‡ Department of Chemistry, University of York, York YO10 5DD, U.K.; § Architecture et Fonction des Macromolécules Biologiques (AFMB), CNRS, Aix-Marseille University, Marseille 13288, France; ∥ Department of Bio-Organic Synthesis, Leiden Institute of Chemistry, Leiden University, Einsteinweg 55, Leiden 2333 CC, The Netherlands

## Abstract

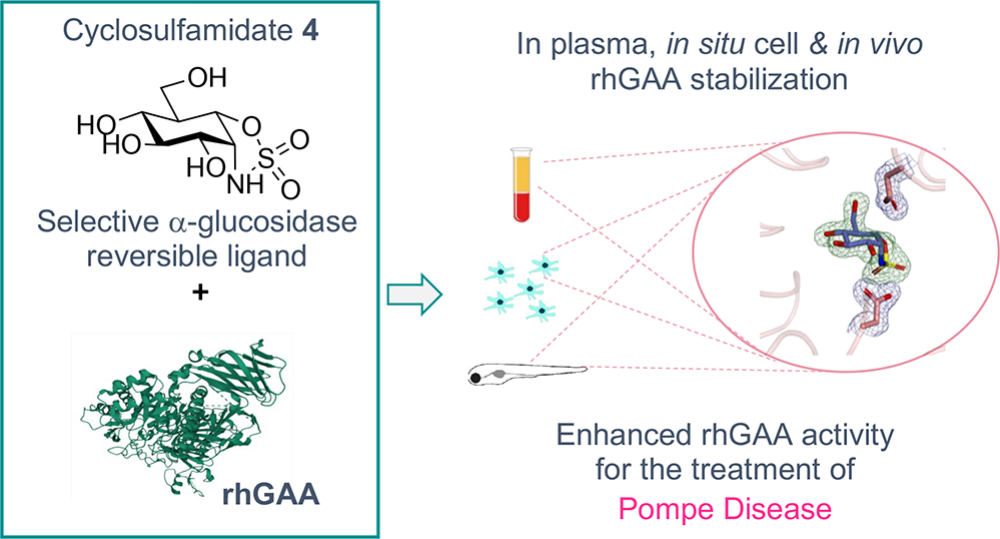

α-Glucosidase inhibitors are potential therapeutics for the treatment of diabetes, viral infections, and Pompe disease. Herein, we report a 1,6-*epi*-cyclophellitol cyclosulfamidate as a new class of reversible α-glucosidase inhibitors that displays enzyme inhibitory activity by virtue of its conformational mimicry of the substrate when bound in the Michaelis complex. The α-d-*glc-*configured cyclophellitol cyclosulfamidate **4** binds in a competitive manner the human lysosomal acid α-glucosidase (GAA), ER α-glucosidases, and, at higher concentrations, intestinal α-glucosidases, displaying an excellent selectivity over the human β-glucosidases GBA and GBA2 and glucosylceramide synthase (GCS). Cyclosulfamidate **4** stabilizes recombinant human GAA (rhGAA, alglucosidase alfa, Myozyme) in cell medium and plasma and facilitates enzyme trafficking to lysosomes. It stabilizes rhGAA more effectively than existing small-molecule chaperones and does so *in vitro*, *in cellulo*, and *in vivo* in zebrafish, thus representing a promising therapeutic alternative to Miglustat for Pompe disease.

## Introduction

Human acid α-glucosidase, also known as acid maltase (GAA, EC 3.2.1.20), is a retaining α-glucosidase of the glycoside hydrolase GH31 family (http://www.cazy.org) and is responsible for the lysosomal degradation of glycogen.^1,2^ Mutations in the gene encoding for GAA can lead to enzyme deficiency which generates multiple phenotypic forms of the lysosomal storage disorder (LSD) Pompe disease (PD).^3,4^ Lysosomal accumulation of glycogen, which is the result of these mutations, causes progressive dysfunction and apoptosis primarily in skeletal and cardiac muscle cells, promoting muscle hypotonia and loss of respiratory and cardiac motor functions, and affects the liver and the nervous system in more severe cases.^5−7^

**Figure 1:**
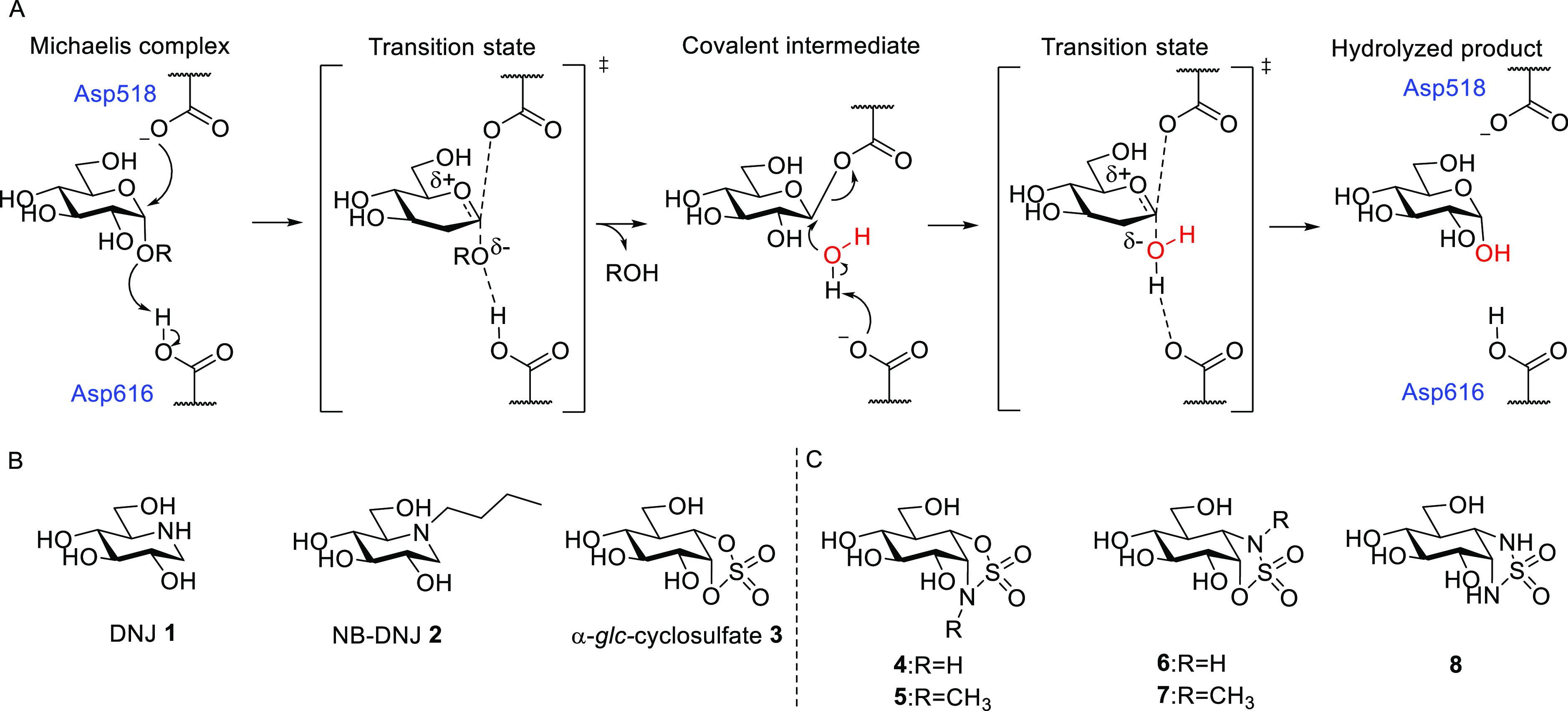
α-Glucosidase mechanism and chemical structures of α-glucosidase inhibitors. (A) Koshland double displacement mechanism of α-glucosidases. (B) Deoxynojirimycin (DNJ **1**, duvoglustat), *N*-butyldeoxynojirimycin (NB-DNJ **2**, miglustat), and epicyclophellitol cyclosulfate **3**. (C) New α-d-glucose-configured cyclosulfamidates **4**–**7** and cyclosulfamide **8**.

The only current treatment for PD comprises enzyme replacement therapy (ERT), in which recombinant human GAA [rhGAA, alglucosidase alpha, Myozyme (ex-US) or Lumizyme (US)] is administered intravenously to the patient.^8,9^ The trafficking and lysosomal delivery and therefore clinical efficacy of the recombinant enzyme are limited due to their plasma instability and massive autophagic buildup in the Pompe skeletal muscle, with only a small part of the administered enzyme reaching its point of destination: muscle tissue.^10,11^ Furthermore, the development of host antibodies against rhGAA also affects the efficacy of the treatment.^12,13^ An alternative therapeutic intervention for LSDs termed pharmacological chaperone therapy (PCT) involves the administration of a small reversible inhibitor. By reversible occupancy of the (endogenous, mutant) enzyme active site, chaperones stabilize its mature protein fold, thereby allowing it to survive the quality control machinery of the endoplasmic reticulum (ER) and assisting its transport toward lysosomes. However, only a small group of Pompe patients carry mutations that respond to PCT.^14^ Combining ERT and PCT was demonstrated in murine Pompe models and shown to be more efficacious than either of the individual treatments alone.^15^ PCT using the iminosugars deoxynojirimycin (DNJ **1**, Duvoglustat)^16^ or *N*-butyldeoxynojirimycin (NB-DNJ **2**, Miglustat) as enzyme stabilizers is currently in phase 2 and 3 clinical trials, either as monotherapy-stabilizing endogenous enzyme or in combination with ERT-stabilizing rhGAA (Figure 1B).^17,18^ Of note, DNJ and NB-DNJ are not selective for GAA and instead target a range of other carbohydrate-processing enzymes such as GBA, GBA2, and GCS.^19^ A small group of reversible GAA inhibitors have been described in the past. *N*-Acetylcysteine, an allosteric positive modulator, is able to stabilize rhGAA, although it does so at concentrations (10 mM) that might not be therapeutically relevant.^20,21^ A set of *in vitro* rhGAA activators were described by Marugan et al. more than a decade ago for which the stabilization mechanism and binding mode remain to be investigated.^22^ More recently, Kato and collaborators described a set of C-branched arabinose- and glucose-configured iminosugars able to inhibit rhGAA *in vitro* at nanomolar concentrations.^23,24^ The most advanced inhibitor, 5-C-heptyl-deoxynojirimycin, despite displaying a nanomolar IC_50_, stabilizes rhGAA in Pompe fibroblasts at 10 μM, and its selectivity toward related glycosidases needs to be determined. This illustrates that high *in vitro* inhibitory potencies do not necessarily correlate with an optimal enzyme stabilization. In principle, the ideal pharmacological chaperone in a combined chaperone-ERT setting binds, and stabilizes, recombinant enzyme (here rhGAA) in circulation but is outcompeted by its natural substrate (here lysosomal glycogen) upon reaching its (sub)cellular target. Fine-tuning this inhibition/binding versus an optimal stabilization effect is not trivial and cannot be predicted by low *in vitro* inhibitory constants.

Retaining α-glucosidases employ a conserved two-step Koshland double displacement mechanism, in which α-glucosidase substrates are hydrolyzed using two key carboxylic acid residues functioning as the catalytic nucleophile (Asp518 in GAA and Asp412 in *Cj*Agd31B, a bacterial homologue of GAA) and catalytic acid/base (Asp616 in GAA and Asp480 in *Cj*Agd31B) (Figure 1A). We have recently described 1,6-*epi*-cyclophellitol cyclosulfate **3** (Figure 1B) as a potent and irreversible covalent retaining α-glucosidase inhibitor with excellent selectivity over retaining β-glucosidases by mimicking the ^4^C_1_ Michaelis complex conformation of α-glucoside substrates when bound in the enzyme active site at the onset of catalytic hydrolysis catalyzed by an aspartic acid.^25^ We recently reported on the development of an α-galactose-configured cyclophellitol cyclosulfamidate as an effective reversible α-galactosidase inhibitor. This sulfamidate was designed based on the rationale that reducing the electrophilicity of the cyclic sulfate by substituting oxygen(s) for less electronegative nitrogen(s) may prevent its nucleophilic displacement by the enzyme’s catalytic nucleophile.^26^ C–N bonds are less polarized than C–O bonds and sulfamidates are thus less electrophilic than sulfates. This α-*gal*-cyclosulfamidate, also a ^4^C_1_ Michaelis complex mimetic, and although less effective than commercial Migalastat, turned out to be an effective reversible α-galactosidase A (α-GalA) inhibitor able to stabilize α-GalA—which in genetically mutated forms is at the basis of Fabry disease—*in vitro* and *in cellulo* at high micromolar concentrations.^26^ Both GAA and α-GalA utilize an identical Koshland double displacement reaction mechanism with ^4^C_1_ (Michaelis) → [^4^H_3_]^‡^ → ^1^S_3_ (intermediate) → [^4^H_3_]^‡^ → ^4^C_1_ (product) as substrate conformational itineraries. Considering the reversibility and enzyme stabilization effect of the α-*gal*-cyclosulfamidate and the nanomolar GAA inhibition by cyclophellitol cyclosulfate **3**, we envisioned that the corresponding α-*glc*-cyclosulfamidates could also lead to competitive α-glucosidase inhibitors that could act as enzyme stabilizers in the context of PD. Herein, we describe the development of epicyclophellitol cyclosulfamidates **4**–**7** and sulfamide **8** and reveal their mechanism of action, their potential as inhibitors for lysosomal, ER, and intestinal α-glucosidases, as well as their superiority as (recombinant) enzyme stabilizers (Figure 1C).

## Results and Discussion

### Design and Synthesis of Human Acid α-Glucosidase Inhibitors

**Scheme 1:**
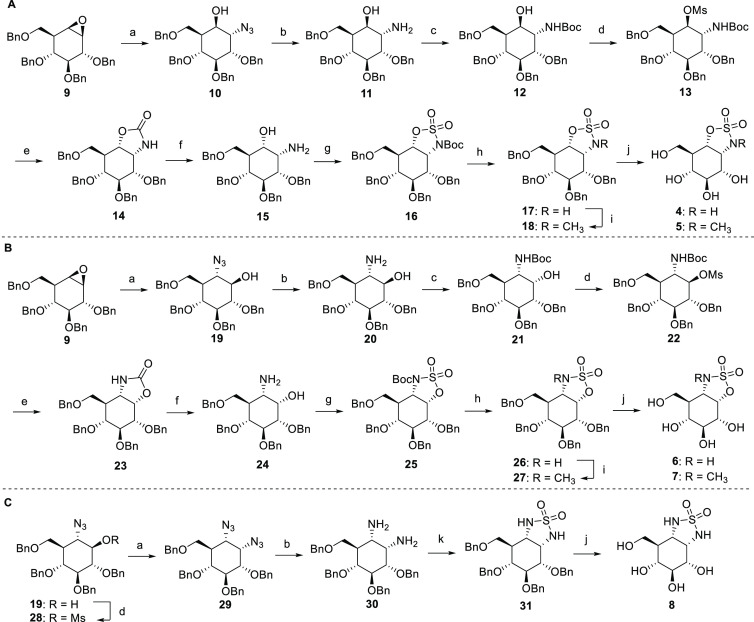
Synthesis of Cyclosulfamidates **4** and **5** (A), Cyclosulfamidates **6** and **7** (B), and Cyclosulfamide **8** (C) Reagents and conditions: (a) NaN_3_, DMF, 18 h, 38% (**10**), 34% (**19**), 71% (**29**); (b) PtO_2_, H_2_, THF, rt, 18 h, 98% (**11**), 97% (**20**), 92% (**30**); (c) Boc_2_O, Et_3_N, DCM, 18 h, rt, 82% (**12**), 78% (**21**); (d) MsCl, Me-imidazole, Et_3_N, CHCl_3_, 5 h, rt, 91% (**13**), 80% (**22**), 88% (**28**); (e) DMF, 24 h, 120 °C, 64% (**14**), 85% (**23**); (f) 1 M NaOH, EtOH, 70 °C, 18 h, 96% (**15**), 82% (**24**); (g) (i) Boc_2_O, Et_3_N, DCM, rt, 18 h; (ii) SOCl_2_, Et_3_N, imidazole, DCM, 15 min, 0 °C; (iii) RuCl_3_, NaIO_4_, 1:1:1 H_2_O, EtOAc, MeCN, 1 h, 0 °C, 77% (**16**), 33% (**25**) over 3 steps; (h) TFA, DCM, rt, 1 h, 62% (**17**), 65% (**26**); (i) MeI, K_2_CO_3_, TBAI, 18 h, rt, 65% (**18**), 79% (**27**); (j) Pd/C, H_2_, MeOH, rt, 18 h, 99% (**4**), 55% (**5**), 87% (**6**), 89% (**7**), 96% (**8**); (k) sulfamide, pyridine, reflux, 6 h, 91%.

The synthesis of sulfamidates **4**–**7** started by nucleophilic addition of sodium azide to tetra-*O*-benzyl-cyclophellitol **9**,^27^ yielding a mixture of trans-azido alcohols **10** and **19**, which were purified and subsequently reduced to trans-amino alcohols **11** and **20** (Scheme 1A,B). *N*-Boc protection of **11** and mesylation of the remaining free hydroxyl of **12** yielded **13** as the only product. In contrast, mesylation of **21** also resulted in the formation of *trans*-oxazolidinone **32** due to double inversion caused by 1-methylimidazole (Scheme 1C and Supporting Information). Heating of **13** and **22** resulted in the formation of oxazolidinones **14** and **23** through neighboring-group participation, which after hydrolysis of the cyclic carbamate gave the desired *cis*-amino alcohols **15** and **24**, respectively. *N*-Boc protection followed by treatment with thionyl chloride and subsequent oxidation gave fully protected α-glc-cyclosulfamidates **16** and **25**, which after global deprotection yielded target compounds **4** and **6**. *N*-Boc deprotection of **16** and **25** and methylation under basic conditions with methyl iodide followed by hydrogenation afforded **5** and **7**. α-*Glc*-cyclosulfamide was synthesized from trans-azido alcohol **19**. Diazido-compound **29** was obtained through mesylation of the remaining hydroxyl and subsequent nucleophilic substitution of mesylate **28** with sodium azide. Reduction of the azides was followed by cyclization of diamine **30** with sulfamide under basic conditions to obtain benzyl-protected cyclosulfamide **31**, which after debenzylation resulted in final cyclosulfamide **8**.

### *In Vitro* Inhibition of Human Acid α-Glucosidase

Having **4**–**8** in hand, we first evaluated their potency as inhibitors of the human glycoprocessing enzymes, α-glucosidase (GAA), ER α-glucosidase II (GANAB), the retaining β-glucosidases, GBA and GBA2, and glucosylceramide synthase (GCS), in comparison to the known GAA inhibitors NB-DNJ **2** and α-*glc*-cyclosulfate **3** (Table 1). Apparent glucosidase IC_50_ values were determined *in vitro* by measuring the inhibition of processing of the respective (alpha or beta) 4-methylumbelliferyl (4-MU)-d-glucopyranoside fluorogenic substrates, whereas GCS inhibition was evaluated in RAW 264.7 cells by measuring inhibitor-dependent glucosylation of C6-NBD-ceramide as the fluorescent substrate. Cyclosulfamidate **4** proved to be a low-micromolar inhibitor of GAA (IC_50_ = 5.17 μM) and is 5 times more potent than NB-DNJ **2** (IC_50_ = 26.7 μM). Furthermore, cyclosulfamidate **4** showed considerably more selectivity compared to NB-DNJ **2**, which inhibits GBA2 (IC_50_ = 957 nM) and GCS (IC_50_ = 43 μM) rather more potently. Methylated analogue **5** proved to be a much weaker inhibitor toward α-glucosidase GAA (IC_50_ = 485 μM) and inactive toward GANAB, suggesting that there is no room for functionalization at the endocyclic nitrogen. In comparison, sulfamidate **6** with an oxygen at the pseudo-anomeric position showed to be a 10-fold less potent lysosomal GAA inhibitor, with comparable activity for the GANAB (IC_50_ = 112 and 47 μM, respectively). Methylation of analogue **6** is somehow tolerated when inhibiting GANAB with methylated cyclosulfamidate **7** showing inhibition of GANAB with an IC_50_ of 132 μM but targeting GBA and GBA2 as well (IC_50_ = 93 and 652 μM, respectively). Finally, the double substitution of endocyclic oxygens by nitrogens in sulfamide **8** is detrimental for α-glucosidase activity.

**Table 1:** Apparent IC_50_ Values for *In Vitro* Inhibition of α-Glucosidases GAA (Myozyme) and GANAB (from Pompe Disease Fibroblast Lysates), β-Glucosidases GBA1 (Cerezyme) and GBA2 (GBA2-Overexpressing HEK293T Lysate), and *In Situ* Cell Inhibition of Glucosylceramide Synthase (GCS) (RAW 264.7 Cells)^a^

compound	*in vitro* GAA IC_50_ (μM)	*in vitro* GANAB IC_50_ (μM)	*in vitro* GBA IC_50_ (μM)	*in vitro* GBA2 IC_50_ (μM)	*in situ* GCS IC_50_ (μM)
**2**	26.7 ± 0.60	153 ± 21.5	>500	0.957 ± 0.499	43.0 ± 3.60
**3**	0.048^b^ ± 1.0 × 10^–4^	0.0260^b^ ± 0.004	>500	62% inhibition at 500 μM	N.D.
**4**	5.17 ± 0.195	496 ± 30.0	>500	>500	>50
**5**	485 ± 146	>500	>500	393 ± 93.7	N.D.
**6**	112 ± 2.54	47.0 ± 1.75	>500	>500	>50
**7**	>1000	132 ± 11.8	93.4 ± 7.83	652 ± 87.3	>50
**8**	>500	>500	>500	>500	>50

a Reported values are mean ± standard deviation from three technical triplicates. N.D.: not determined.

b Values in accordance with ref (25).

**Figure 2:**
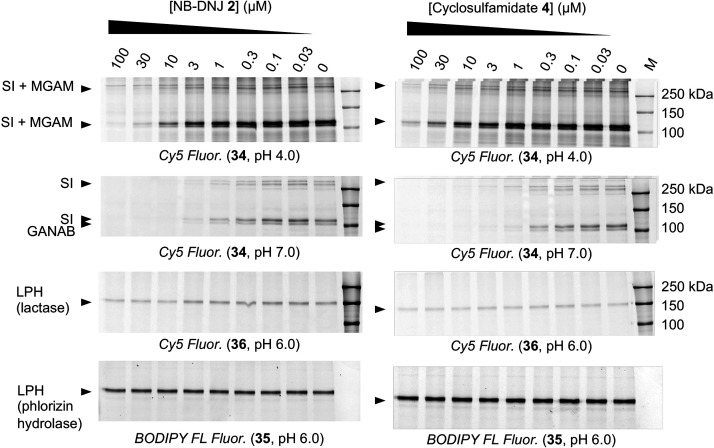
Competitive ABPP (cABPP) in mouse intestine homogenates. Mouse duodenum (60 μg protein) extracts were preincubated with cyclosulfamidate **4** or NB-DNJ **2** (0–100 μM) for 30 min at 37 °C at pH 4.0, 6.0, or 7.0, followed by labeling of sucrase-isomaltase (SI) and maltase-glucoamylase (MGAM) by Cy5 α-glucosidase ABP **34** (pH 4.0 or 7.0). Lactase pocket of lactase-phlorizin hydrolase (LPH) was labeled with Cy5 ABP **36**, and the phlorizin hydrolase pocket of LPH was labeled by preblocking the lactase pocket of LPH with β-galactosidase ABP **36** for 30 min at 37 °C at pH 6.0, followed by subsequent incubation with **4** and NB-DNJ **2**, and final labeling with green BODIPY β-glucosidase ABP **35**.

The selectivity of sulfamidate **4** for α-glucosidases over β-glucosidases and β-galactosidases was further investigated by competitive activity-based protein profiling (cABPP) in human fibroblast homogenates using α-glucosidase ABP **34**, broad-spectrum β-glucosidase ABP **35**, and β-galactosidase ABP **36** (Figure S1). cABPP in mouse intestine homogenates demonstrated that sulfamidate **4** and iminosugar **2** inhibit intestinal sucrase-isomaltase (SI) and maltase-glucoamylase (MGAM), which are intestinal α-glucosidases involved in food processing that have been extensively targeted to prevent and treat type II diabetes mellitus.^28,29^ Inhibition of gastrointestinal α-glucosidases with antidiabetic drugs has been related to undesired effects, including flatulence, diarrhea, and abdominal pain. Cyclosulfamidate **4** inhibits SI and MGAM but does so about a 100-fold less potently than it inhibits rhGAA or GANAB, and we conclude that it has a more beneficial therapeutic window in this regard compared to NB-DNJ **2**. Sulfamidate **4** and NB-DNJ **2** inhibit neither β-glucosidase nor β-galactosidase activities of lactase-phlorizin hydrolase (LPH) (Figure 2).

A closer look into inhibition kinetics with inhibitor **4** or **6** preincubated with Myozyme at concentrations of their corresponding apparent IC_50_ values for different time periods (15, 30, 45, 60, 120, 180, and 240 min) showed that while cyclosulfate **6** is an irreversible inhibitor (a decrease in residual α-glucosidase activity is observed with longer incubations), cyclosulfamidate **4** inhibits rhGAA in a competitive manner (Figure S2). Kinetic parameters determined with increasing 4-MU-α-glucoside concentrations proved that cyclosulfamidate **4** reversibly inhibits GAA with a *K*_i_ of 3.40 μM, approximately 5-fold more potent than NB-DNJ **2** (*K*_i_ 15.2 μM) (Table 2). Contrary to the previously observed inactivity of the galactose-configured cyclosulfamidate analogue toward α-GalA,^26^ cyclosulfamidate **6** in turn proved to be an irreversible inhibitor of GAA with a *K*_I_ of 378 μM and *k*_inact_ of 0.35 min^–1^.

**Table 2:** Inhibition Constants (*K*_i_, *K*_I_, and *k*_inact_) in Recombinant Human α-Glucosidase (rhGAA, Myozyme)

compound	kinetic parameters in rhGAA
NB-DNJ **2**	*K*_i_ = 15.2 μM
**4**	*K*_i_ = 3.4 μM
**6**	*K*_I_ = 456 μM and *k*_inact_ = 0.35 min^–1^

### Structural Characterization of Enzyme–Inhibitor Complexes

**Figure 3:**
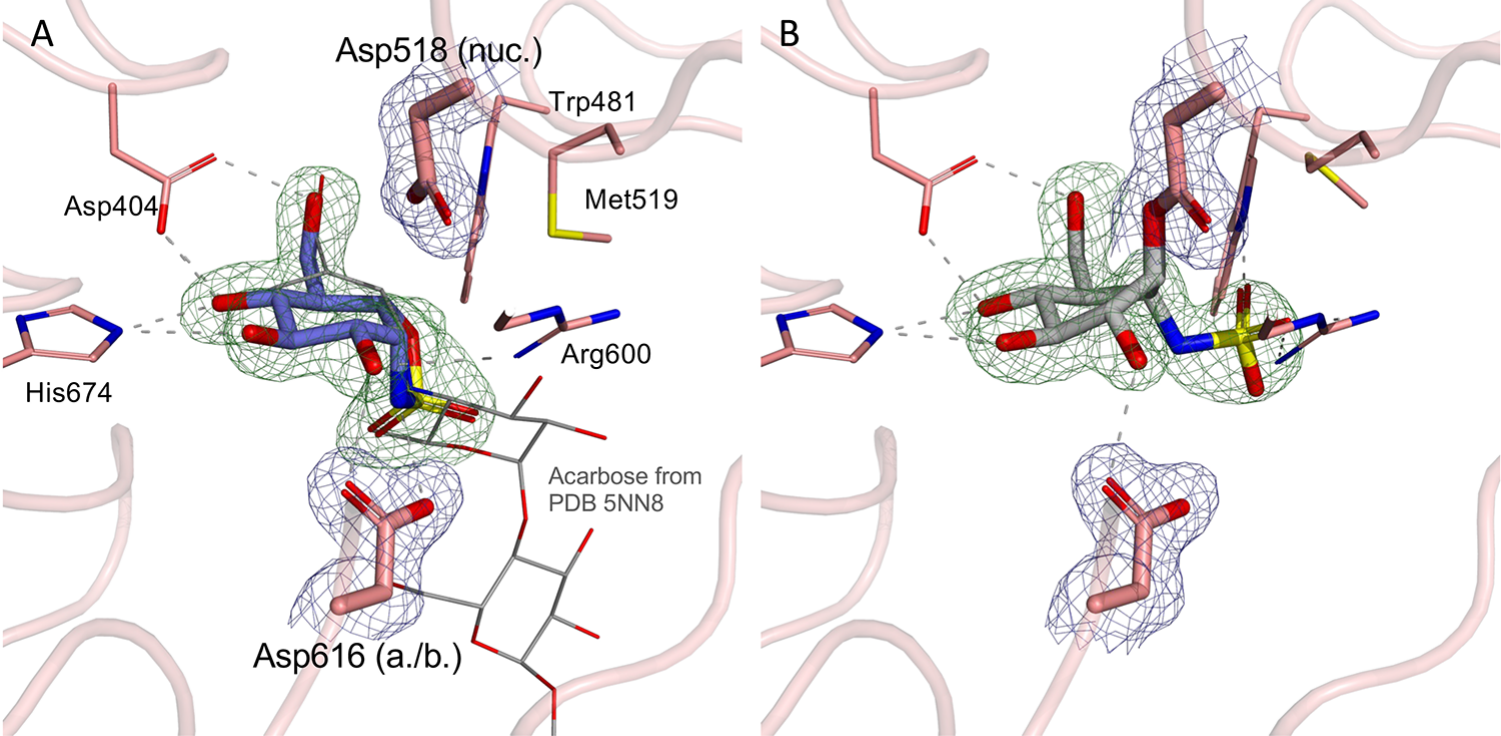
α-*Glc*-cyclosulfamidates **4** and **6** in complex with the proteolytically digested form of rhGAA. (A) α-*Glc-*cyclosulfamidate **4** forms a ^4^C_1_ nonreacted complex with rhGAA. (B) α-*Glc*-cyclosulfamidate **6** reacts covalently with the rhGAA nucleophile (Asp518) adopting a final ^1^S_3_ conformation.

Crystallographic studies with the proteolytically digested form of human recombinant rhGAA (Myozyme)^21^ (Figure 2) and the bacterial GH31 α-glucosidase/transglucosidase *Cj*Agd31B homologue from *Cellvibrio japonicus*(30) (Supporting information, Figure S3) further demonstrated the binding motifs of cyclosulfamidates **4** and **6**. The X-ray structure of human α-glucosidase rhGAA in complex with sulfamidate **4** revealed that it binds the enzyme active site adopting a ^4^C_1_ Michaelis-type conformation in a noncovalent manner (Figure 3A). The interactions of the cyclitol moiety of sulfamidate **4** with rhGAA are virtually identical to those observed in previous reported complex structures of rhGAA with the GAA inhibitors, acarbose, 1-deoxynojirimycin (DNJ), and *N*-hydroxyethyl-DNJ (NHE-DNJ),^21^ notably with Arg600, His674, Asp404, and the acid/base Asp616, strengthened by solvent-mediated interactions with Asp443, Asp616, Asp645, and Trp481 (Figure S4). The endocyclic nitrogen of the sulfamidate moiety establishes a tight hydrogen-bonding interaction with the acid/base Asp616, providing a structural rational for the lack of inhibition of the methylated analogue **5** due to steric hindrance. The endocyclic nitrogen and the sulfate moieties of sulfamidate **4** overlap spatially with the nitrogen and subsite +1 aglycone atomic features, respectively, of acarvosine reported previously^21^ (Figure S4), reinforcing that sulfamidate **4** is a true substrate Michaelis complex mimetic for GAA. Structural data for the rhGAA-sulfamidate **6** complex clearly revealed that the cyclosulfamidate with the oxygen attached to the pseudo-anomeric position acts as an electrophilic trap, yielding a covalent complex with the enzyme and adopting a final ^1^S_3_ conformation in the enzymatic pocket after covalent reaction with the catalytic nucleophile Asp518^31^ (Figure 3B). The interactions of the cyclitol moiety of sulfamidate **6** are essentially identical to those observed in the rhGAA-sulfamidate **4** complex, though the sulfamidate moiety engenders steric constraints on the active-site scaffold. The bulky sulfamidate, liberated from the cyclic restraint of the cyclophellitol framework, pushes outward Met519, Trp481, and the associated loop region (Figure S6). The energy cost of this structural perturbation is compensated for by several H-bonding interactions between the sulfamidate and rhGAA active-site residues, pleading for stabilization of the abortive complex.

### *In Vitro*, *In Cellulo*, and *In Vivo* Stabilization of rhGAA

**Figure 4:**
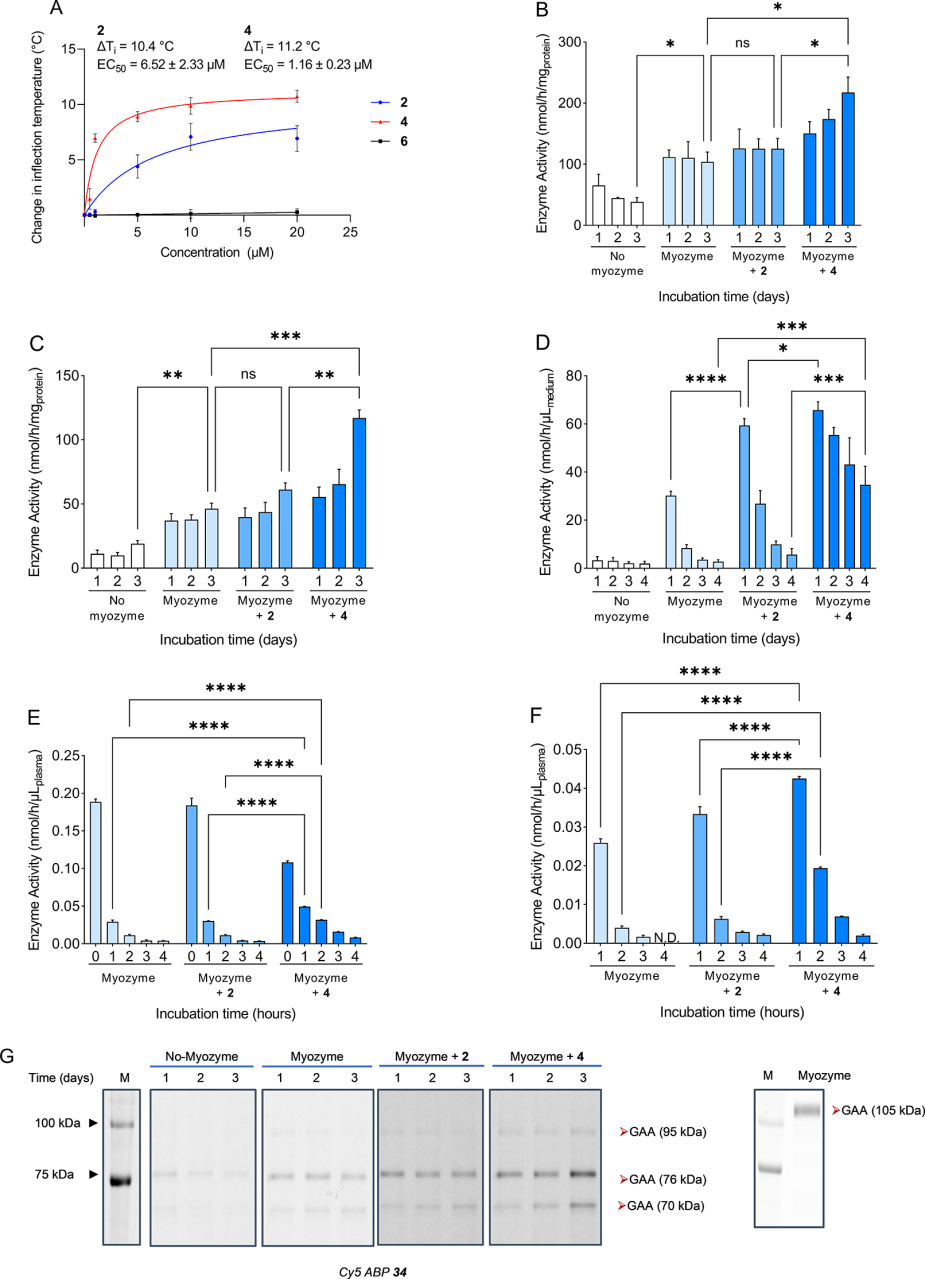
GAA activity in Pompe disease fibroblasts. (A) Effect of **2**, **4**, and **6** on the thermostability of rhGAA. The graph shows heat-induced denaturation profiles of rhGAA in complex with **2** (blue) **4** (red) and **6** (black) (B,C). GAA activity in lysates of cultured Pompe disease fibroblasts treated with **2** or **4** in combination with or without Myozyme. Fibroblasts from adult Pompe disease patient and incubated for 1–3 days with Myozyme (alglucosidase alpha, 10 nM) in combination with or without 20 μM NB-DNJ **2** or cyclosulfamidate **4**. Experiments were performed in biological triplicates and technical triplicates; graphs B and C were obtained at two different cell passage numbers. Data are depicted as mean ± SD and analyzed using a two-way ANOVA with Tukey’s multiple comparison test. *p < 0.05. (D–F) Myozyme (10 nM) was incubated in (D) cell culture medium, (E) plasma from healthy individuals, or (F) plasma from an adult Pompe patient (genotype: p.L355P; p.R672W) under ERT (last infusion 11 days before sampling) for 1–4 days (in medium) or 1–4 h (in plasma) in combination with or without 20 μM NB-DNJ **2** or cyclosulfamidate **4**. Experiment was performed in biological triplicates, *N* = 2 technical replicates. Data are depicted as mean ± SD and analyzed using a two-way ANOVA with Tukey’s multiple comparison test. *p < 0.05. (G) GAA activity in lysates of cultured Pompe disease fibroblasts from (B) visualized with an activity-based probe (ABP) **34**. Lysates (3 μg per well) were first incubated with ABP **35** (Bodipy green fluorescence) to block GBA activity and GAA (three isoforms) was then visualized by incubation with ABP **34** (Cy5 fluorescence).

We next studied the ability of cyclosulfamidate **4** to stabilize α-glucosidases *in vitro* and *in situ*. Thermostability assays (TSAs) showed that α-*glc*-cyclosulfamidate **4** stabilizes rhGAA with a maximum shift in inflection temperature (ΔTi_max_) of 11.2 °C and a half maximal effective concentration (EC_50_) of 1.16 μM, whereas α-*glc*-cyclosulfamidate **6** does not prevent enzyme unfolding at increasing temperature (Figures 4A and S7). Similar assays with *Cj*Agd31B using a Sypro Orange dye revealed that α-*glc*-cyclosulfamidate **4** stabilizes the bacterial analogue *in vitro* as well with a ΔTm_max_ of 5.2 °C and an EC_50_ of 134 μM (Figure S6). Prompted by these positive results, we also investigated the capacity of cyclosulfamidate **4** to stabilize rhGAA (10 nM final concentration) in cell medium and in fibroblasts from Pompe adult patients with almost no α-glucosidase activity. Two different cell passages of Pompe fibroblasts were incubated with either NB-DNJ **2** (at 20 μM) or cyclosulfamidate **4** (at 20 μM) in the presence or absence of rhGAA Myozyme (10 nM) for 1–3 days, and activity was measured every 24 h without refreshing the media (Figure 4B,C). A two-fold increase of rhGAA activity is observed 3 days after the treatment of fibroblasts with rhGAA and **4** compared to fibroblasts treated only with rhGAA, indicating that sulfamidate **4** is able to stabilize the enzyme in cells. This increase in rhGAA activity is also observed after longer incubations (from day 1 to day 3), suggesting that cyclosulfamidate **4** may stabilize the enzyme in the medium and more enzyme is available for cell internalization and transport toward the lysosomes. Indeed, rhGAA showed complete degradation in cell medium over 4 days, which is reduced to 50% degradation by the addition of cyclosulfamidate **4** (20 μM), a significant improvement compared to the 5% remaining with NB-DNJ **2** (Figure 4D). We also investigated whether stabilization also occurs in the plasma of healthy individuals (Figure 4E) and two different Pompe patients: an adult Pompe patient with genotype: p.L355P; p. R672W under ERT (last infusion 11 days before sampling) (Figure 4F) and an adult Pompe patient with genotype: c.-32-13 T > G; p.N403K under no treatment (Figure S8). In contrast to the cell medium, degradation of rhGAA in plasma occurs much faster, with more than 85% being degraded after just 1 h. Addition of cyclosulfamidate **4** (20 μM) slows such degradation in plasma from healthy and Pompe patients with similar efficacy: more than 3-fold increase in rhGAA activity is observed after 2 h in plasma from an adult Pompe patient (Figure 4F). Of note, in this experiment, we observed more than doubled stabilization for cyclosulfamidate **4** when compared to that for NB-DNJ **2**.

The transport and processing of endocytosed lysosomal rhGAA in cultured human skin fibroblasts are well documented, and differences in apparent molecular mass between the exogenous rhGAA (105 KDa) and corresponding lysosomal forms (70 and 76 KDa) are due to differences in glycosylation.^32,33^ Notably, we can detect by cABPP an increase of rhGAA’s 70 and 76 kDa isoforms over time, which indicate that cyclosulfamidate **4** may assist in the transport of rhGAA to lysosomes in Pompe fibroblasts as well (Figure 4G). The rhGAA stabilization as revealed by the enhanced turnover of 4-MU glucoside (Figure 4B,C) correlates well with the enhanced labeling intensity of rhGAA with α-glucosidase Cy5 ABP **34** (Figure 4G). Of note, the stabilization effect of cyclosulfamidate **4** is superior to the one observed with NB-DNJ **2** also by ABPP analysis.

**Figure 5:**
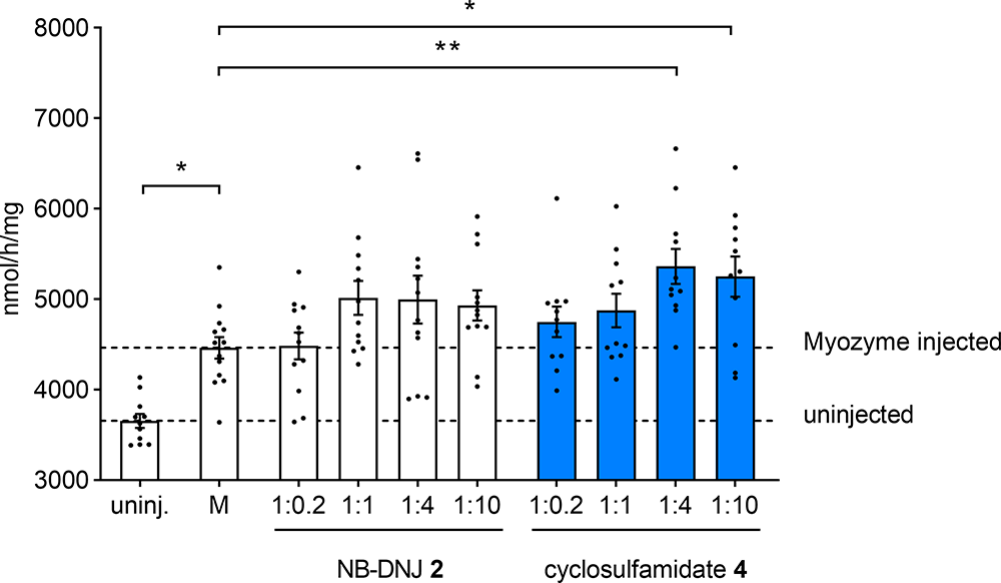
GAA activity in homogenates of 5 days postfertilization (dpf) zebrafish injected with Myozyme alone or in combination with **2** or **4**. Myozyme (34 pmol in 1 nL) or Myozyme combined with NBD-DNJ **2** or sulfamidate **4** (molar ratios of 1:0.2, 1:1, 1:4, and 1:10 Myozyme:stabilizer) was injected into the sinus venous/duct of Cuvier of 2 dpf wild-type zebrafish. GAA activity was measured in homogenates of 5 dpf zebrafish larvae. Injections were performed at three independent times, and at least three biological replicates were measured per injection/zebrafish. Uninjected zebrafish, *n* = 11; Myzoyme injected (M), *n* = 13; M + 6.8 pmol 2, *n* = 12; M + 34 pmol 2, *n* = 12; M + 136 pmol 2, *n* = 12; M + 340 pmol 2, *n* = 12; M + 6.8 pmol 4, *n* = 11; M + 34 pmol 4, *n* = 11; M + 136 pmol 4, *n* = 11; M + 340 pmol 4, *n* = 11. Data are depicted as mean ± SD and analyzed using a one-way ANOVA with Dunnett’s multiple comparison test with Myozyme alone as control column. *p < 0.05.

In order to analyze rhGAA stabilization *in vivo*, we developed an enzyme stabilization assay in an easily accessible *invivo* animal system. For this, wild-type zebrafish of 2 days postfertilization (2 dpf) were injected intravenously into the sinus venous/duct of Cuvier with Myozyme (34 pmol) or a combination of Myozyme (34 pmol) and either NB-DNJ **2** or cyclosulfamidate **4** at different molar ratios (1:0.2, 1:1, 1:4, or 1:10 Myozyme:chaperone). After 5 dpf, zebrafish embryos were homogenized, and α-glucosidase activity was measured using the 4-MU-d-glucopyranoside fluorogenic substrate. Zebrafish larvae injected with Myozyme showed a significant increase in GAA activity (7.5%) compared to nontreated controls. Zebrafish treated with Myozyme in combination with cyclosulfamidate **4** showed a significantly increased GAA activity (±25% at 1:4 ratio) at 5 dpf compared to the zebrafish injected with only Myozyme (Figure 5). This indicates that sulfamidate **4** is also able to stabilize recombinant enzyme *in vivo* and that this stabilization is slightly more efficient than the one observed with NB-DNJ **2**. High concentrations of reversible inhibitors **2** and **4** (1:10 ratio) showed a small decrease in GAA activity, indicating that a high concentration of ligand might inhibit Myozyme as well as endogenous GAA. Of note, this experiment is limited by the amount of volume (2 nL) and concentration of the rhGAA stock that can be injected in the 2 dpf zebrafish.

## Conclusions

In conclusion, 1,6-*epi-*cyclophellitol cyclosulfamidate **4** is a selective reversible α-glucosidase ligand that binds through ^4^C_1_ conformational Michaelis complex mimicry. Kinetic and crystallographic studies of rhGAA demonstrate that the position of the endocyclic nitrogen dictates the reactivity of these cyclic sulfamidates, and whereas cyclosulfamidate **6** reacts covalently, sulfamidate **4** binds in a competitive manner, providing a new chemical space for potential α-glucosidase stabilizers. By reversible occupancy of the enzyme active site, cyclosulfamidate **4** stabilizes rhGAA and prevents its degradation *in vitro* in cell medium and plasma from Pompe patients, *in situ* in Pompe fibroblasts, and *in vivo* zebrafish larvae, facilitating the enzyme transport toward the lysosome. We conclude that cyclosulfamidate **4** presents a superior stabilization and selectivity profile compared to NB-DNJ **2**, the benchmark compound in clinical combination ERT/PCT trials for the treatment of Pompe disease.

## Supporting Information Available

Experimental data and procedures, crystallographic data, and synthesis (PDF)
