# Supplementary material for: 1,6-epi-Cyclophellitol Cyclosulfamidate Is a Bona Fide Lysosomal α-Glucosidase Stabilizer for the Treatment of Pompe Disease
Source: J Am Chem Soc. 2022 Aug 2;144(32):14819–27. doi: 10.1021/jacs.2c05666 (PMC9389588; doi:10.1021/jacs.2c05666)
Supplement: Supplementary file 1 — ja2c05666_si_001.pdf [file ja2c05666_si_001.pdf]

Supporting Information for

1,6-*Epi*-Cyclophellitol Cyclosulfamidate Is a Bona Fide Lysosomal α-Glucosidase Stabilizer for the Treatment of Pompe Disease

Ken Kok[a], Chi-Lin Kuo[a], Rebecca E. Katzy[a], Lindsey T. Lelieveld[a], Liang Wu[b,c], Véronique Roig-Zamboni[d], Gijsbert A. van der Marel[e], Jeroen D. C. Codée[e], Gerlind Sulzenbacher[d], Gideon J. Davies[b], Herman S. Overkleeft[e], Johannes M. F. G. Aerts[a] and Marta Artola*[a]

[a]Department of Medical Biochemistry, Leiden Institute of Chemistry, Leiden University, Einsteinweg 55, 2333 CC Leiden, The Netherlands.

[b]Department of Chemistry, York Structural Biology Laboratory, University of York, Heslington, York YO10 5DD, United Kingdom.

[c]Current address: Structural Biology Department, The Rosalind Franklin Institute, Harwell Campus, Didcot, OX11 0FA, United Kingdom.

[d]Architecture et Fonction des Macromolécules Biologiques (AFMB), CNRS, Aix-Marseille University, Marseille, France.

[e]Department of Bio-organic Synthesis, Leiden Institute of Chemistry, Leiden University, Einsteinweg 55, 2333 CC Leiden, The Netherlands.

Corresponding author: m.e.artola@lic.leidenuniv.nl.

TABLE OF CONTENTS

1. Supporting Figures and Tables S3
2. Materials and Methods S9
   - 2.1. Biochemical and Biological Methods S9
     - 2.1.1. *In vitro* apparent IC_{50} measurements S9
     - 2.1.2. Competitive activity-based protein profile (cABPP) in mouse intestine homogenates S9
     - 2.1.3. Time-dependent inhibition of GAA S9
     - 2.1.4. Kinetic studies S10
     - 2.1.5. Thermo-stability assays (TSAs) S10
     - 2.1.6. *In vitro* stabilization of acid α-glucosidase (rhGAA, Myozyme®) in fibroblasts S10
     - 2.1.7. *In vivo* stabilization of rhGAA in plasma S11
     - 2.1.8. *In vivo* stabilization of rhGAA in zebrafish embryo S11
   - 2.2. Crystallographic data collection and refinement statistics S12
     - 2.2.1. Crystallographic data collection and refinement of recombinant human acid α-glucosidase (rhGAA, Myozyme®) S12
     - 2.2.2. Crystallographic data collection and refinement of bacterial α-glucosidase *Cj*Agd31B S12
3. Chemical Synthesis ` S13
   - 3.1. General Experimental Details S13
   - 3.2. Synthesis and Characterization Data of Compounds S14
     - 3.2.1. Synthesis and Characterization Data of **4** and **5** S14
     - 3.2.2. Synthesis and Characterization Data of **6** and **7** S17
     - 3.2.3. Synthesis and Characterization Data of **8** S20
4. NMR Spectra S23
5. References S52

1. Supporting Figures and Tables

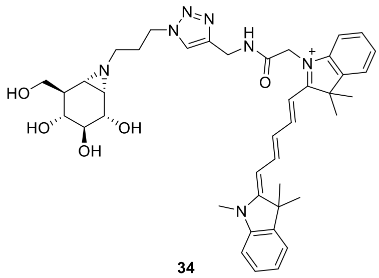

34

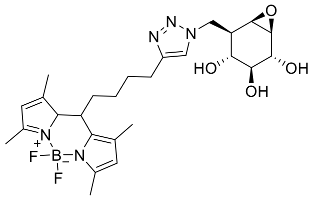

35

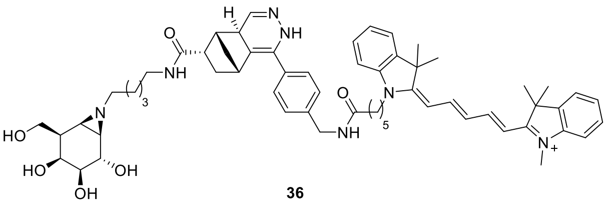

36

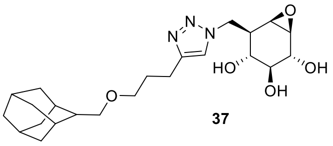

37

Figure S1. Structure of activity-based probes (ABPs) and inhibitors used in this study: α-glucosidase ABP **34** (JJB383), selective GBA β-glucosidase ABP **35** (MDW933), α-galactosidase ABP **36** (TB652) and inhibitor **37** (ME656).

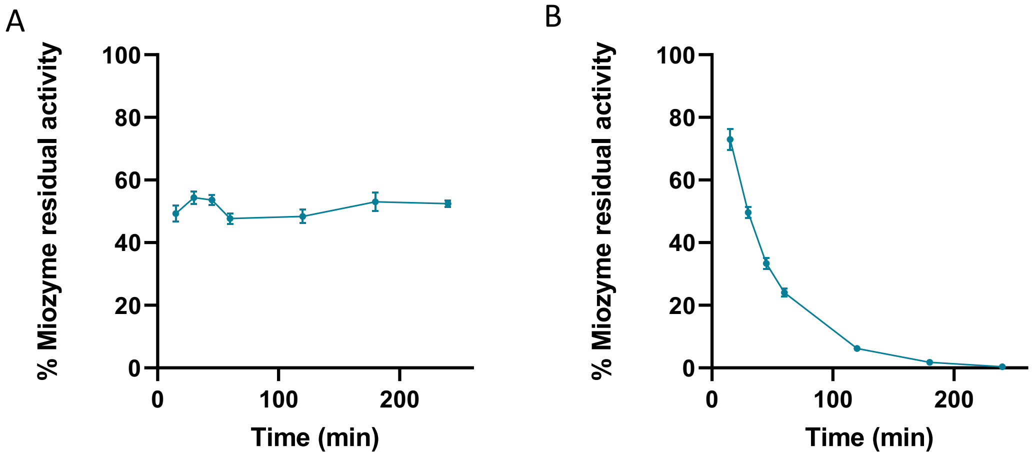

Figure S2. Time dependent inhibition of acid α-glucosidase (GAA) by cyclosulfamidates **4** and **6**. Residual activity of recombinant human acid α-glucosidase (GAA, myozyme) with different pre-incubation times (15, 30, 45, 60, 120, 180 and 240 min) in the presence of **4** and **6** at their *in vitro* apparent IC_{50} concentrations (66 μM and 5.1μM respectively). A: Time-dependent inhibition curve of **4**. B: Time dependent inhibition curve of **6**.

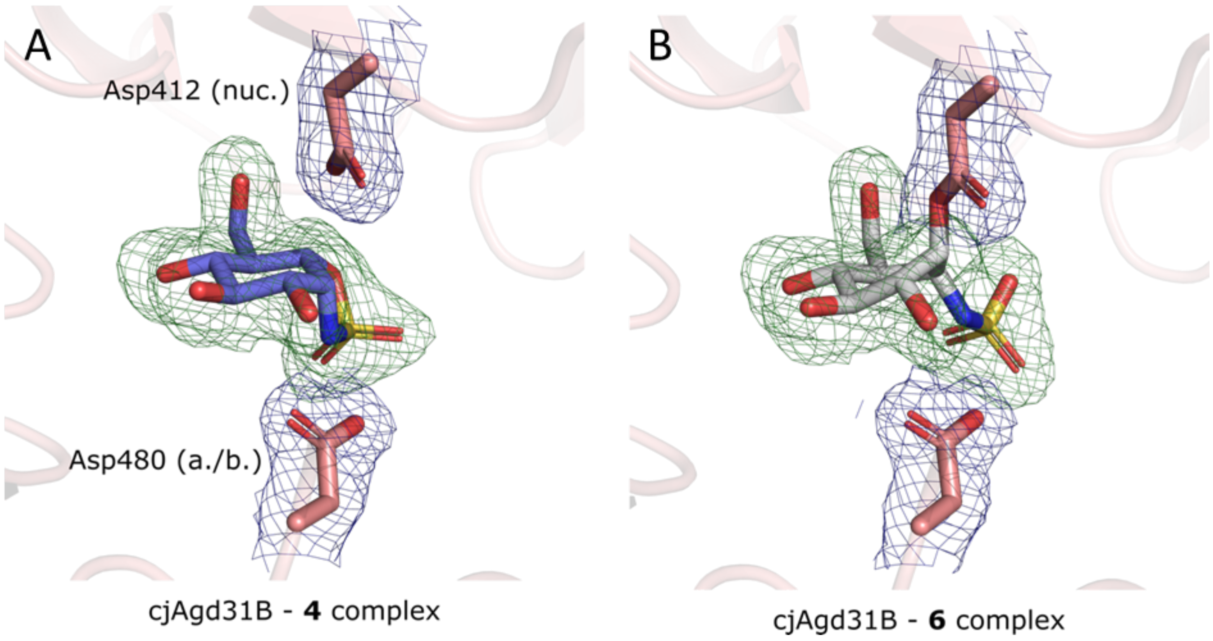

Figure S3. Bacterial α-glucosidase *Cj*Agd31B in complex with cyclosulfamidates **4** and **6**. A: *Cj*Agd31B forms a non-covalent complex with **4**, with the ligand adopting a $^{4}C_{1}$ conformation in the enzyme active site. B: Cyclosulfamidate **6** reacts with *Cj*Agd31B, producing a $^{1}S_{3}$ covalent complex linked to the enzyme's nucleophilic residue (Asp412).

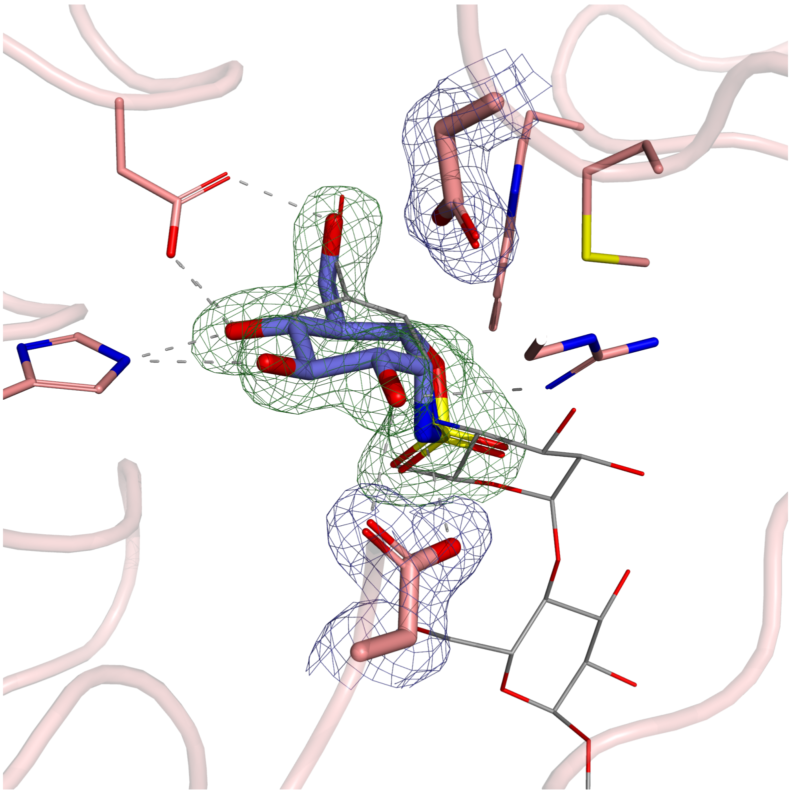

Figure S4. Recombinant human acid α-glucosidase (rhGAA) in complex with cyclosulfamidate **4**. The enzyme forms a non-covalent complex with **4**, with the ligand adopting a $^{4}C_{1}$ conformation in the enzyme's active site.

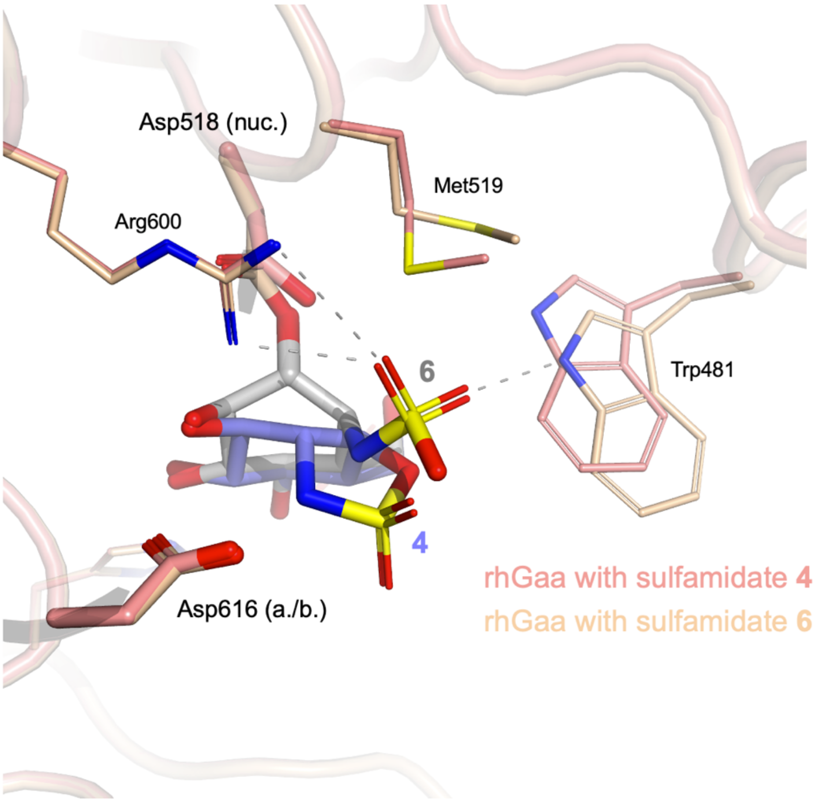

Figure S5. Overlay of recombinant human acid α-glucosidase (rhGAA) in complex with cyclosulfamidates **4** and **6**. The active site forms a non-covalent complex with **4**, with the ligand adopting a $^{4}C_{1}$ conformation. Cyclosulfamidate **6** reacts with rhGAA producing a $^{1}S_{3}$ covalent complex linked to the enzyme's nucleophilic residue (Asp518).

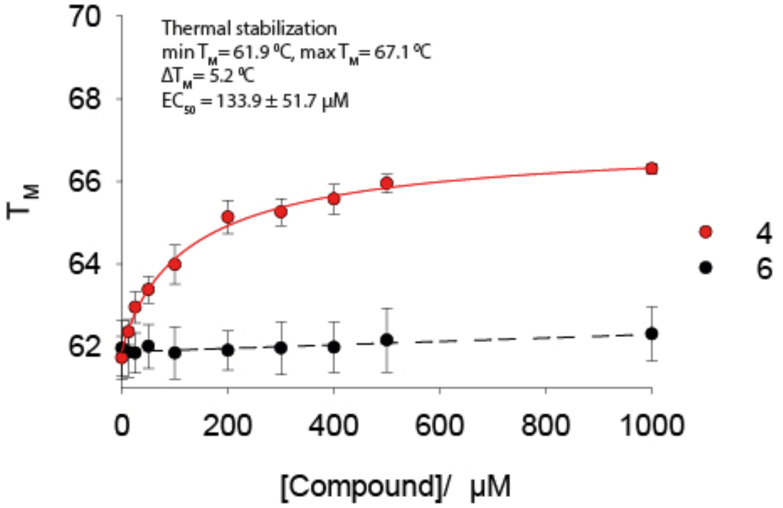

Figure S6 The effect of **4** and **6** on the thermostability of *Cj*Agd31B. Graph shows the heat-induced melting profiles of *Cj*Agd31B in complex with **4** (red) and **6** (black).

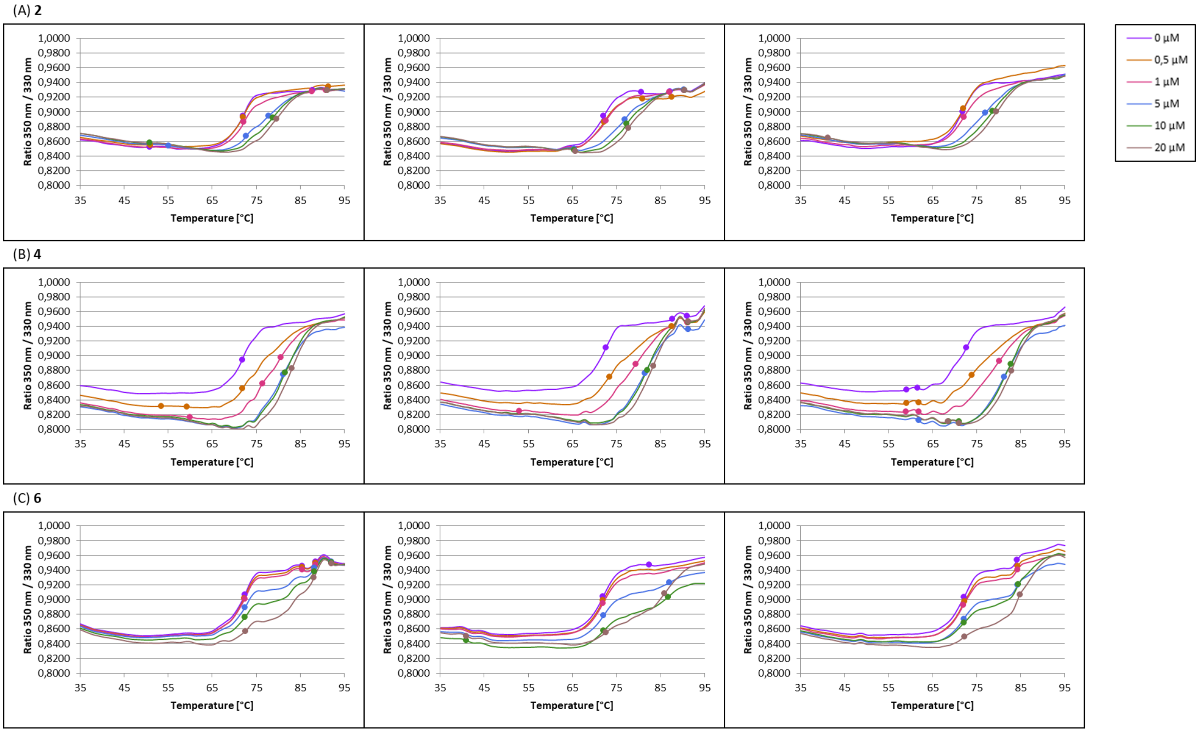

Figure S7. The unfolding curves measured by the Tycho NT-6 of Myozyme preincubated with 0 µM – 20 µM of compounds (A) **2**, (B) **4**, and (C) **6**.

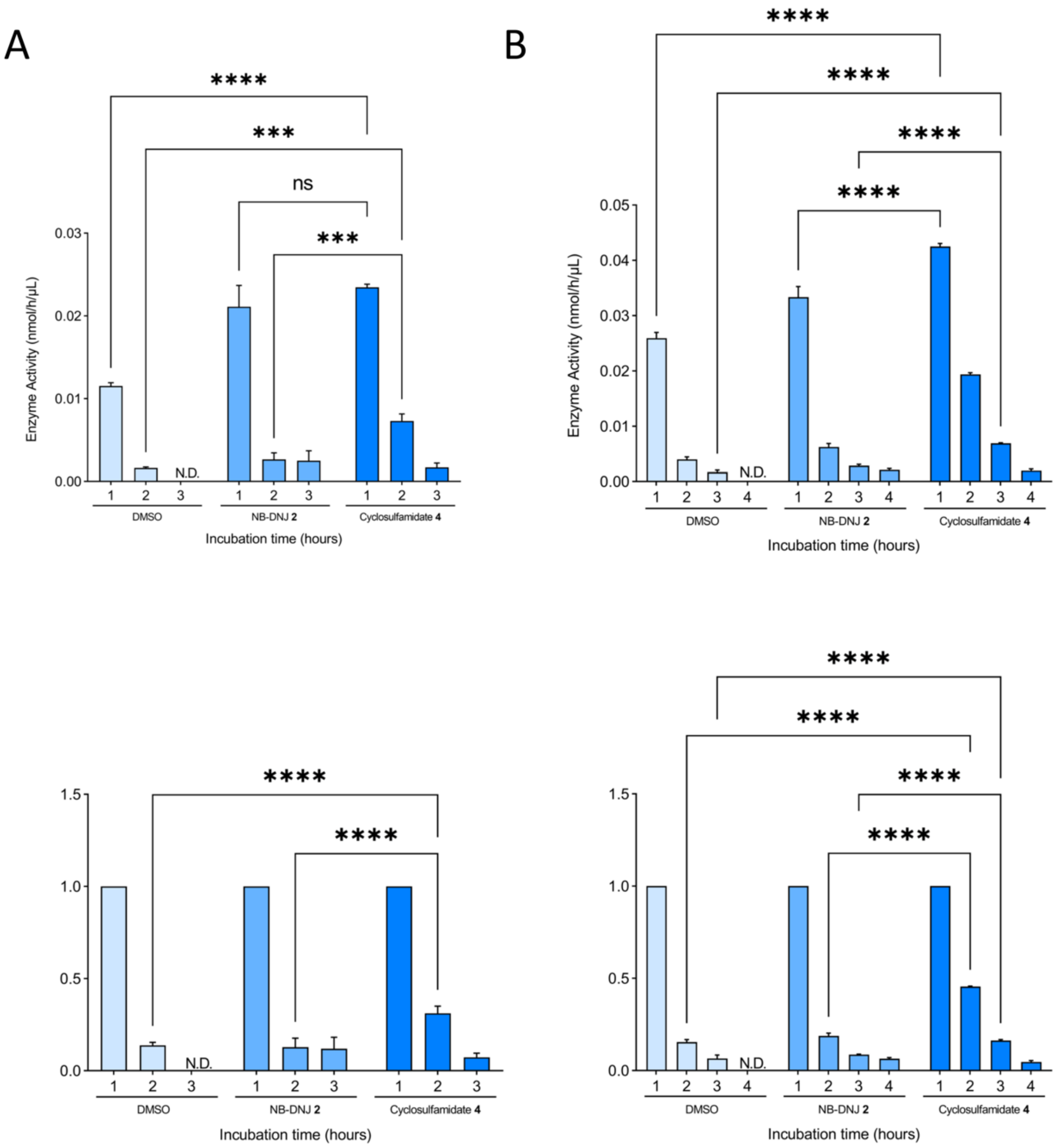

Figure S8. Myozyme (10 nM) was incubated in (A) plasma from adult Pompe patient (genotype: p.L355P; p. R672W) under ERT (last infusion 11 days before sampling) or (B) plasma from an adult Pompe patient with genotype: c.-32-13T>G; p.N403K under no treatment, for 1-4 days in combination with or with-out 20 µM NB-DNJ **2** or cyclosulfamidate **4**. Experiment was performed in biological triplicates, N = 2 technical replicates. Upper graphs show enzyme activity (nmol/h/µL) and bottom graphs show percentage enzyme activities normalized to enzyme activity after 1 h incubation. Data are depicted as mean ± SD and analyzed using a Two-Way ANOVA with Tuckey's multiple comparisons test. *$p < 0.05$.

Table S1. Data collection and refinement statistics

	*Cj*Agd31B-**4** complex	*Cj*Agd31B-**6** complex	rhGAA-**4** complex	rhGAA-**6** complex
PDB entry	7P4C	7P4D	7P2Z	7P32
**Data collection**				
Space group	P622	P622	$P2_12_12_1$	$P2_12_12_1$
Cell dimensions				
a, b, c (Å)	197.0, 197.0, 102.2	197.1, 197.1, 102.2	97.2, 102.5, 129.0	97.0, 102.6, 129.1
α, β, γ (°)	90, 90, 120	90, 90, 120	90, 90, 90	90, 90, 90
Resolution (Å)	102.16–1.86 (1.89–1.86)	98.54–1.85 (1.88–1.85)	47.66-1.85 (1.88-1.85)	47.68-1.82 (1.85-1.82)
CC (1/2)	0.999 (0.618)	0.999 (0.696)	0.998 (0.742)	0.999 (0.492)
R_{meas}	0.13 (2.90)	0.14 (2.74)	0.13 (1.68)	0.11 (1.79)
$I / \sigma I$	14.5 (1.3)	14.2 (1.4)	14.5 (2.1)	15.9 (1.5)
Completeness (%)	100 (99.5)	100 (99.3)	100 (99.9)	99.8 (96.2)
Redundancy	24.6 (24.1)	24.7 (25.2)	13.6 (14.0)	13.5 (11.7)
Wilson B (A^2)	35.28	32.35	20.85	23.36
**Refinement**				
Resolution (Å)	102.36–1.86	98.54–1.85	47.69-1.85	47.72-1.82
No. reflections	92720	94592	104870	110011
R_{work} / R_{free}	0.19/0.23	0.19/0.22	14.84/17.54	15.00/17.54
No. atoms				
Protein + N-glycans	6276	6274	6852	6811
Ligands/Ions	32/51	37/46	70/34	60/34
Water	431	485	778	680
B-factors				
Protein	43.5	41.1	29.76	30.29
Ligands/Ions	55.5/77.4	54.8/76.6	49.13/54.19	41.58/53.31
Water	42.8	41.8	39.32	39.39
R.m.s. deviations				
Bond lengths (Å)	0.010	0.010	0.009	0.009
Bond angles (°)	1.60	1.62	1.51	1.51

Values in parentheses are for highest-resolution shell.

2. Materials and Methods

2.1 Biochemical and Biological Methods

2.1.1 *In vitro* apparent IC_{50} experiments

To determine *in vitro* apparent IC_{50} values, 40 µL of enzyme-mixture was pre-incubated with 40 µL of inhibitor for 30 min in the following buffers: GAA in 150 mM McIlvaine buffer pH 4.0, 0.1% bovine serum albumin (BSA) (w/v) and 2% DMSO (v/v). GANAB in 150 mM McIlvaine buffer pH 7.0, 0.1% BSA (w/v) and 2% DMSO (v/v). GBA1 in 150 mM McIlvaine buffer pH 5.2, 0.2% Taurocholate (w/v), 0.1% Triton X-100 (v/v), 0.1% BSA (w/v), 2% DMSO (v/v). GBA2 in 150 mM McIlvaine pH 5.8, 0.1% BSA (w/v).

After preincubation, 25 µL of E+I mixture was added to a 96 wells plate and 100 µL substrate solution in the appropriate buffer was subsequently added. GAA activity was measured using final concentrations of 47 nM enzyme (Myozyme) and 3.0 mM 4-MU-α-D-glucopyranoside, for 30 minutes at 37 ℃. GANAB activity was measured using wt fibroblasts and 2.4 mM 4-MU-α-D-glucopyranoside, for 2 h at 37 ℃. GBA1 activity was measured using final concentrations of 0.7 nM enzyme (Cerezyme) and 3.0 mM 4-methylumbeliferone(4-MU)-β-D-glucopyranoside, for 30 min at 37 ℃. GBA2 residual activity was measured using cellular homogenates of HEK293T over-expressing GBA2 pre-incubated for 30 min with a GBA1 inhibitor (1 µM **37** (ME656), see Figure S1 for chemical structure), and further incubation with 3.0 mM 4-methylumbeliferone(4MU)-β-D-glucopyranoside for 1 h at 37 ℃.

Finally, all enzyme reactions were quenched with 1 M NaOH-Glycine (pH 10.3) and hydrolyzed 4-MU fluorescence measured with a LS55 fluorescence spectrophotometer (Perkin Elmer; λ_{EX} 366 nm, λ_{EM} 445 nm). Values plotted for [I] are those in the final reaction mixture, containing E + I + S. *In vitro* IC_{50} values were determined from technical triplicate.

2.1.2 Competitive activity-based protein profiling (cABPP) in mouse intestine homogenates

Duodenum from wild type mice were isolated according to guidelines approved by the ethical committee of Leiden University (DEC#13191). Homogenization was performed in KPi buffer (25 mM K_2HO_4/KH_2PO_4, 0.1% (v/v) Triton X-100, protease inhibitor cocktail (EDTA-free, Roche)) with silent crusher S equipped with Typ 7 F/S head (30,000 rpm, 3 cycles of 7 sec) on ice, at a ratio of 4 µL buffer per 1 mg tissue. Protein concentration was determined with BCA Protein Assay Kit (Thermo Fisher). For cABPP, duodenum homogenates were firstly equilibrated for 5 min on ice in 4 volumes of McIlvaine buffer at pH 4.0, 6.0, or 7.0. Samples (10 µL, 60 µg total protein) were then subjected to inhibitor incubation (**2** or **4**, various concentrations, 2.5 µL in 2.5% (v/v) DMSO/H_2O) for 30 min at 37 °C, before ABP labeling (2.5 µL) for 15 min at 37°C with JJB383 **34** (3 µM in 3 % (v/v) DMSO/McIlvaine buffer at pH 4.0 (SI + MGAM) or pH 7.0 (SI + GANAB)), MDW933 **35** (6 µM in 3 % (v/v) DMSO/McIlvaine buffer at pH 6.0) for LPH phlorizin hydrolase pocket, or TB652 **36** (6 µM in 3 % (v/v) DMSO/McIlvaine buffer at pH 6.0) for LPH lactase pocket (see Figure S1 for chemical structures). Samples were subsequently denatured and subjected to SDS-PAGE and fluorescent scanning according to previously described methods.[1]

2.1.3 Time-dependent inhibition of GAA

To study the type of inhibition 20 µL rhGAA (Myozyme, 47 nM in 150mM McIlvaine buffer pH 4.0, 0.1% BSA) was pre-incubated for, 15, 30, 45, 60, 120, 180, 240 minutes at 37℃ with 20 µL of inhibitors **4** (66 µM in 150 mM McIlvaine buffer pH 4.0, 0.1% BSA, 2% DMSO) or **6** (5.1 µM in 150 mM McIlvaine buffer pH 4.0, 0.1 % BSA, 2% DMSO). After preincubation, 25 µL of enzyme + inhibitor mixture was added to a 96 well plate and 100 µL substrate solution (3.0 mM 4-MU-α-D-glucopyranoside) in the previous buffer was added and incubated for 30 minutes at 37 ℃. Finally, enzyme reactions were quenched with 1M NaOH-Glycine (pH 10.3) and liberated 4-MU fluorescence was measured with a LS55 fluorescence spectrophotometer (Perkin Elmer; λ_{EX} 366 nm, λ_{EM} 445 nm). Time was plotted against residual enzyme activity (Figure S2). Time-dependent GAA inhibition assays were performed in technical triplicate.

2.1.4 Kinetic studies

Kinetic paramaters for inhibition of GAA were determined using a fluorogenic substrate assay involving the simultaneous exposure of enzyme substrates and inhibitors and various concentrations. During the enzymatic reaction, the concentration of enzyme (rhGAA) was 47 nM in 150mM McIlvaine buffer pH 4.0, 0.1% BSA and the concentration of substrate was 3 mM. For each inhibitor triplicate sets of 2 mL Eppendorf tubes were prepared. For irreversible inhibitor kinetics, 8.13 µL inhibitor at various concentrations (diluted in DMSO 200x reaction concentration) was added to an Eppendorf tube followed by 154.4 µL McIllvaine buffer (150 mM McIlvaine buffer pH 4.0) and 1300 µL substrate mixture (2.4 mM 4-MU-α-D-glucopyranoside, pH 4.0, 0.1% (w/v) BSA). The 2 mL eppendorf tubes were pre-heated for 10 min in a thermal shaker at 37℃. For each inhibitor concentration the enzyme was prepared by diluting with McIllvaine buffer in an Eppendorf tube to a volume of 1250 µL. A negative control (enzyme blanc) was prepared in a separate 2 mL Eppendorf tube by using denatured enzyme (rhGAA) which was pre-boiled (98℃ for 5 min). Both enzyme and blanc were pre-warmed on a thermoshaker at 37℃ for 10 min. Prior to the reaction a 96-black well plate was prepared with 200 µL stop buffer (1M NaOH-Glycine, pH 10.3) per well. The t=0 samples were prepared by filling the first columns with 12.5 µL enzyme (or enzyme blanc in the last column) and 112.6 of each of the substrate-inhibitor mix. To start the reaction 137.6 µL aliquots of pre-warmed enzyme to each of the pre-warmed 2 mL Eppendorf tubes containing the substrate-inhibitor mixture with an interval of 10 seconds. To ensure proper mixing of the separate components during addition of the enzyme aliquots the 2 mL Eppendorf tubes were continuously shaken in a thermoshaker at 37℃. At t=4 min, 6 min, 8 min, 10 min, duplicates aliquots of 125 µL from each of the reaction tubes were transferred to the 96-well plate that contained stop buffer with a 10 second interval between samples. The plate was shortly vortexed after which 4-MU fluorescence was measured immediately. When reversible inhibition was observed similar experimental conditions were used with relevant inhibitor concentration (100 µM) and a range of substrate (4-MU-α-D-glucopyranoside, pH 4.0, 0.1% (w/v) BSA) concentrations. The observed pseudo first order inactivation rate (k_{obs}) was calculated for each concentration of each inhibitor by fitting the data with the one-phase exponential association function: 4-MU fluorescence = A*(1-e^(k_{obs}*t)) using GraphPad Prism. The obtained k_{obs} values for each concentration of each inhibitor were then plotted against the inhibitor concentration, the resulting plots were plotted using a linear function which gives combined apparent inhibition parameter k_{inact}/K'_I as the slope. k_{inact}/K_I was derived from k_{inact}/K'_I by correcting for the presence of competing 4-MU-α-D-glucopyranoside substrate using the relationship: $K'_I = K_I(1+[S]/K_M)$. K_I values for reversible inhibition were determined by linear mixed-inhibition kinetics.

2.1.5 Thermo-stability assays (TSAs)

Thermofluor methods for TSA of bacterial α-Glucosidase *Cj*Agd31B

Thermal shift assay (TSA) reactions were assembled to a final volume of 25 µL in 50 mM Sodium Citrate pH 6.5 buffer, containing 2 µM *Cj*Agd31B, 2x Sypro Orange dye (Sigma, 5000x stock concentration), and 0–1000 µM inhibitor (Figure S4). All conditions were carried out in triplicate.

Reactions were run on a Stratagene Mx3005P qPCR instrument (Agilent), across a temperature range of 25–95°C, linearly incremented at a rate of 1°C per 30 seconds. Sypro Orange fluorescence was measured every 30 seconds, using wavelengths of λ_{ex} = 517 nm and λ_{em} = 585 nm.

Fluorescence measurements *vs* temperature for each TSA reaction were fitted to a 5-parameter sigmoidal function (y = T_{min} + (T_{max} - T_{min})/(1 + e(A – x)B)^C with melting temperature (T_M) determined as the midpoint of the plotted curves. Calculated T_M values were used to generate a plot of T_M *vs* inhibitor concentration for each inhibitor, which was subsequently fitted to a 4 parameter logistic function (y = T_{M_min} + (T_{M_max} - T_{M_min})/(1 + (x/EC_{50})^B) to give the EC_{50} and maximal ΔT_M of thermal stabilization for each inhibitor.

Stability of Myozyme determined by the Tycho NT.6

In a final volume of 30 µL, Myozyme (1 µM) was incubated with 0 µM – 20 µM of compound in 50 mM Sodium Citrate buffer pH 6.5. Samples were incubated at RT for two hours, after which each sample was measured in triplicate on the Tycho NT.6. Inflection points (defined as the maximum of the first derivative) as determined by the Tycho NT.6 were plotted against the compound concentration and the EC_{50} and maximum shift in inflection temperature (ΔT_i) were calculated using GraphPad Prism version 9.

2.1.6 *In vitro* stabilization of α-glucosidase A in fibroblasts

Adult Pompe fibroblasts, cultured in DMEM/F12 + 10 % FCS + P/S at 5 % CO_2, were seeded during split (1:4) in 24 well plates. Medium was refreshed weekly, and experiments were started two weeks after the 1:4 passage date. Recombinant human GAA (rhGAA, Myozyme) was diluted to 10 nM in DMEM/F12 and compounds in DMSO were added to final concentration of 20 µM, with a final DMSO concentration of 0.5 %. After incubation, cells were washed three times with PBS and scraped in 30 µL lysis buffer (20 mM KPi pH 6.5 supplemented with 0.1 % Triton X-100 and protease inhibitor, no EDTA). After 30 min incubation on ice, lysates were stored at -80 °C until use. To examine the stability of Myozyme under experimental conditions, samples of the used medium with Myozyme and compounds were also incubated at 37 °C, 5 % CO_2, and frozen at -80 °C after collection.

Protein concentrations were determined using the Pierce BCA kit, with lysates diluted 3x.

Specific GAA activity was determined using 3.0 mM 4-MU-α-D-glucopyranoside substrate in GAA buffer (150 mM McIlvaine buffer pH 4.0 with 0.1 % BSA) with 0.1 % DMSO. In triplicate, 2 µL lysate was diluted with 23 µL GSS buffer and incubated with 100 µL substrate for 4 hours at 37 °C. The reaction was quenched with 200 µL 1 M glycine-NaOH pH 10.3. Free 4-MU fluorescence was measured on an LS55 fluorescence spectrophotometer (Perkin Elmer; λ_{EX} 366 nm, λ_{EM} 445 nm).

Lysates were diluted with KPi buffer to 3 µg per well, keeping the total volume lower than 7 µL. McIlvaine buffer (150 mM, pH 4.0) was added to 12 µL. In sequence, the ABPs (200 nM ABP **34** followed by 500 nM ABP **33**) were added and each incubated for 30 min at 37 °C. After incubation the reaction was stopped by adding Laemmli sample buffer and incubation at 95 °C for 5 min. Samples were run on a 7.5 % SDS-PAGE gel and scanned using a Typhoon variable mode imager (GE Healthcare Bio-Science Corp., Piscataway, NJ).

2.1.7 *In vitro* stabilization of rhGAA in plasma

Recombinant human GAA (rhGAA, Myozyme) was diluted to 10 nM in plasma from healthy individuals (Sanquin, The Netherlands) or Plasma from adult Pompe patients kindly donated by the biobank from patients affected by ALS, neuromuscular and lysosomal diseases (University Hospital of Udine, Italy): patient 1 with genotype: p.L355P; p. R672W under ERT (last infusion 11 days before sampling) and patient 2 with genotype: c.-32-13T>G; p.N403K under no treatment. DMSO or compounds in DMSO were added to final concentration of 20 µM, with a final DMSO concentration of 0.5 %. Samples were taken every hour and kept on ice or frozen at -80 °C until α-glucosidase activity analysis. Specific GAA activity was determined as described above, with an incubation time of 2 hours.

2.1.8 *In vivo* stabilization of rhGAA in zebrafish embryo

Zebrafish husbandry and embryo injections.
Zebrafish (Strain AB/TL) were housed at Leiden University, The Netherlands, and maintained and handled in compliance with the directives of the local animal welfare committee (Instantie voor Dierwelzijn, IvD, Leiden) and guidelines specified by the EU animal Protection Directive 2010/63/EU. Wildtype embryos were acquired by natural spawning at the onset of the light period and embryos were raised in egg water (60 µg/L Instant Ocean Sera Marin™ aquarium salts (Sera; Heinsberg, Germany). Injection mixtures were prepared by combining Myozyme (40 µM) with DMSO, NBD-DNJ **2** or sulfamidate **4**, in different molar ratios, in 0.5% Phenol Red solution in DPBS (Sigma). Final mixtures contained 34 µM Myozyme without inhibitor or with 6.8 µM, 34 µM, 136 µM or 340 µM inhibitor for 1:0.2, 1:1, 1:4 or 1:10 molar ratio of Myozyme:inhibitor respectively, and 0.95% (v/v) DMSO. Two day old zebrafish (52-56 hours post-fertilization) were anesthetized in 0.01% tricaine and 1 nL of the Myozyme mixture (with or without inhibitor) was injected into the sinus venous/duct of Cuvier. After injection, the zebrafish larvae were washed twice with egg water and raised to 5 dpf at 28.5 °C. Pools of five 5 dpf zebrafish larvae were harvested, homogenized in 100 µL of KPi Buffer and 12.5 µL of this lysate was used to measure GAA activity as described above with 4-MU-α-glucoside as fluorogenic substrate (Figure 5). Injections were performed two independent times and at least three biological replicates were measured per injection.

2.2 Crystallographic data collection and refinement statistics

2.2.1 Crystallographic data collection and refinement of recombinant human acid α-glucosidase (rhGAA, Myozyme®)

The enzyme source were residual amounts of the infusions of Myozyme® (Sanofi Genzyme, Cambridge, MA) administrated for the treatment of Pompe patients at the Department of Translational Medical Sciences, Federico II University, Naples, Italy. Sample conditioning and crystallization were performed as described previously.[2] Complex crystals were obtained by adding small amounts of powder of cyclosulfamidates **4** and **6,** respectively, to the crystallization droplets, followed by incubation for ~1 h. Crystals were cryo-protected with reservoir solution supplemented with 15% (v/v) glycerol prior flash cooling in liquid N_2. Diffraction data were collected at beamline Proxima2 of the Synchrotron Soleil, Gif-sur-Yvette, France. Data were integrated and scaled with XDS[3] and the CCP4 software suite.[4] The structures were solved by difference Fourier synthesis with REFMAC5[5] using the native structure of rhGAA (PDB entry 5NN3) as starting model. Ligand coordinates were generated with jLigand[6] and the models were adjusted with subsequent rounds of Refmac5[5] and Coot[7], respectively. Indices for the R_{free} cross-validation data sets were taken over from PDB entry 5NN4. Model quality was assessed with internal modules of Coot[7] and with the Molprobity server.[8] Figures were generated with Pymol (The PyMOL Molecular Graphics System, Version 2.3.5, Schrödinger, LLC.)

2.2.2 Crystallographic data collection and refinement of bacterial α-glucosidase *Cj*Agd31B

*Cj*Agd31B expression, purification and crystallization was carried out as previously described.[9] Ligand complexes were obtained by soaking *Cj*Agd31B crystals in cryoprotectant solution (0.1 M HEPES pH 7.0, 2 M Li_2SO_4, 2% PEG400) supplemented with 5 mM ligand for 2 h, before harvesting and flash cooling in liquid N_2 for data collection.

Data were collected at beamline I04 of the Diamond light source, UK. Reflections were autoprocessed using the xia2 pipeline[10] of the CCP4 software suite (Table S1). Complexes were solved by directly refining against an unliganded *Cj*Agd31B structure (PDB accession 4B9Y) using REFMAC5[11], then further improved through subsequent rounds of manual model building and refinement using Coot[12] and REFMAC5 respectively. All ligand coordinates were built using jLigand.[6] Figures were generated using Pymol.

3 Chemical synthesis

3.1 General experimental details

All reagents were of experimental grade and were used without further purification unless stated otherwise. Dichloromethane (DCM) and tetrahydrofuran (THF) were stored over 3 Å molecular sieves and *N,N*-dimethylformamide (DMF) was stored over 4 Å molecular sieves, which were dried *in vacuo* before use. All reactions were performed under an N_2 atmosphere unless stated otherwise. Reactions were monitored by analytical thin layer chromatography (TLC) using Merck aluminum sheets pre-coated with silica gel 60 with detection by UV-absorption (254 nm) and by spraying with a solution of $(NH_4)_6Mo_7O_{24}{\cdot}H_2O$ (25 g/L) and $(NH_4)_4Ce(SO_4)_4{\cdot}H_2O$ (10 g/mL) in 10% sulfuric acid followed by charring at ~150 ℃ or by spraying with an aqueous solution of $KMnO_4$ (7%) and K_2CO_3 (2%) followed by charring at ~150 ℃. Column chromatography was performed manually using either Baker or Screening Device silica gel 60 (0.04-0.063 mm) or a Biotage Isolera™ flash purification system using silica gel cartridges (Screening Device SilicaSep HP, particle size 15-40 μm, 60A) in the indicated solvents. ^{1}H-NMR and ^{13}C-NMR spectra were recorded on Bruker AV-500 (500/126 MHz), and Bruker AV-400 (400/101 MHz) spectrometer in the given solvent. Chemical shifts are given in ppm (δ) relative to the chloroform, methanol or dimethylsulfoxide residual solvent peak or tetramethylsilane (TMS) as internal standard. All given ^{13}C-NMR spectra are proton decoupled. The following abbreviations are used to describe peak patterns when appropriate: s (singlet), d (doublet), t (triplet), q (quartet), m (multiplet), Ar (aromatic), C_q (quarternary carbon). 2D NMR experiments (HSQC, COSY) were carried out to assign protons and carbons of the new structures and numbering and assignation follows the general numbering shown in Figure S9. High-resolution mass spectra (HRMS) of compounds were recorded with an LTQ Orbitrap (Thermo Finnigan) equipped with an electronspray ion source in positive mode (source voltage 3.5 kV, sheath gas flow 10 mL/min, capillary temperature 250 ℃) with resolution R = 60000 at m/z (400 mass range m/z = 150 – 2000) and dioctyl phthalate (m/z = 391.28428) as a lock mass. The high-resolution mass spectrometer was calibrated prior to measurements with a calibration mixture (Thermo Finnigan). Optical rotations were measured on a Anton Paar MCP automatic polarimeter (Sodium D-line, λ = 589 nm). LC-MS analysis was performed on a LCQ Advantage Max (Thermo Finnigan) ion-trap spectrometer (ESI+) coupled to a Surveyor HPLC system (Thermo Finnigan) equipped with a C18 column (Gemini, 4.6 mm x 50 mm, 3 μM particle size, Phenomenex) equipped with buffers A: H_2O, B: acetonitrile (MeCN) or an Agilent technologies 1260 infinity LC-MS with a 6120 Quadrupole MS system equipped with buffers A: H_2O, B: acetonitrile (MeCN) and C: 100 mM NH_4OAc. IR spectra were recorded on a Shimadzu FTIR-8300 and are reported in cm^{-1}. α-Glucosidase ABP JJB 383 **34**[13], selective GBA β-glucosidase ABP MDW 933 **35**[14], α-galactosidase ABP TB652 **36**[15] and inhibitor ME656 **37**[16] were synthesized following previously described procedures.

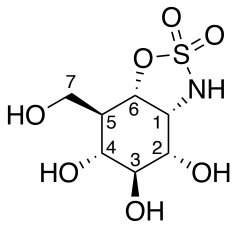

4

Figure S9 General numbering.

3.2 Synthesis and characterization data

3.2.1 Synthesis and characterization data of 4 and 5

(1*R*,2*S*,3*S*,4*S*,5*R*,6*S*)-2-azido-3,4,5-tris(benzyloxy)-6-((benzyloxy)methyl)cyclohexan-1-ol (10) and (1*R*,2*S*,3*R*,4*R*,5*S*,6*S*)-2-azido-4,5,6-tris(benzyloxy)-3-((benzyloxy)methyl)cyclohexan-1-ol (11).
Epoxide **9**[17] (0.57 g, 1.06 mmol) was dissolved in anhydrous DMF (22.3 mL) and NaN_3 (1.38 g, 21.2 mmol, 20 eq) was added to the mixture. The reaction was stirred overnight at 80 °C. The reaction mixture was allowed to cool down to rt, diluted with H_2O and the aqueous phase was extracted with Et_2O (2x). The combined organic layers were washed with H_2O and brine, dried over $MgSO_4$, filtered and concentrated *in vacuo*. The crude reaction mixture was purified by silica gel chromatography (Pentane/EtOAc 90:10 → 70:30) to obtain **10** (0.23 g, 0.4 mmol, 38%) and **19** (0.21 g, 0.36 mmol, 34%).

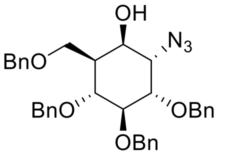

Azide (10): ^{1}H NMR (500 MHz, $CDCl_3$) δ 7.39 – 7.35 (m, 2H, CH_{Ar}), 7.35 – 7.24 (m, 16H, CH_{Ar}), 7.21 – 7.18 (m, 2H, CH_{Ar}), 4.95 (d, J = 10.8 Hz, 1H, CH*H*Ph), 4.93 (d, J = 10.8 Hz, 1H, CH*H*Ph), 4.82 (d, J = 10.7 Hz, 1H, CH*H*Ph), 4.77 (d, J = 11.5 Hz, 1H, CH*H*Ph), 4.73 (d, J = 11.5 Hz, 1H, CH*H*Ph), 4.50 (d, J = 10.9 Hz, 1H, CH*H*Ph), 4.46 (d, J = 11.7 Hz, 1H, CH*H*Ph), 4.39 (d, J = 11.7 Hz, 1H, CH*H*Ph), 4.11 – 4.03 (m, 4H, CH*H*Ph, C-1, C-2, C-7), 3.97 – 3.91 (m, 2H, CH-4, CH-6), 3.85 (t, J = 9.3 Hz, 1H, CH-3), 3.67 (dd, J = 9.2, 2.4 Hz, 1H, CH-7), 1.91 (dq, J = 11.3, 2.5 Hz, 1H, CH-5), 1.57 (s, 1H, OH). ^{13}C NMR (126 MHz, $CDCl_3$) δ 138.9, 138.7, 138.2, 137.1 (4C_q), 128.8, 128.6, 128.5, 128.5, 128.3, 128.1, 128.0, 127.99, 127.9, 127.9, 127.8, 127.7 (12CH_{Ar}), 84.2 (C-3), 80.3 (C-2), 76.6 (C-4), 75.9, 75.7, 73.9, 73.5 (4CH_2Ph), 72.8 (C-1), 70.2 (C-7), 62.7 (C-6), 41.2 (C-5). HRMS: calcd. for $[C_{37}H_{35}N_3NaO_5]^+$ 602.26254; found 602.26228, calcd. for $[C_{37}H_{39}N_4O_5]^+$ 597.30715; found 597.30691.

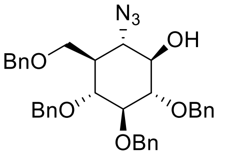

Azide (19): ^{1}H NMR (400 MHz, $CDCl_3$) δ 7.39 – 7.24 (m, 18H, CH_{Ar}), 7.19 (dd, J = 7.4, 2.0 Hz, 2H, CH_{Ar}), 4.96 (d, J = 11.3 Hz, 1H, CH*H*Ph), 4.87 (d, J = 11.1 Hz, 3H, CH*H*Ph), 4.73 (d, J = 11.2 Hz, 1H, CH*H*Ph), 4.54 (d, J = 10.7 Hz, 1H, CH*H*Ph), 4.49 (d, J = 11.8 Hz, 1H, CH*H*Ph), 4.43 (d, J = 11.7 Hz, 1H, CH*H*Ph), 3.82 – 3.73 (m, 1H, CH*H*Ph), 3.73 – 3.68 (m, 1H, CH*H*Ph), 3.66 (dd, J = 9.2, 2.4 Hz, 1H, CH-7), 3.61 – 3.51 (m, 2H, CH-4, CH-7), 3.48 (d, J = 10.8 Hz, 1H, CH-1), 3.37 (t, J = 9.2 Hz, 1H, CH-3), 2.54 (d, J = 2.1 Hz, 1H, CH-6)), 1.56 (s, 1H, OH), 1.49 (tt, J = 11.1, 2.3 Hz, 1H, CH-5). ^{13}C NMR (101 MHz, $CDCl_3$) δ 138.5, 138.4, 138.3, 138.2 (4C_q), 128.8, 128.6, 128.59, 128.5, 128.2, 128.1, 128.0, 127.97, 127.92, 127.83 (10CH_{Ar}), 85.63 (C-3), 83.09 (C-2), 77.82 (C-4), 76.08 (C-1), 75.85, 75.76, 75.68, 73.28 (4CH_2Ph), 64.80 (C-7), 61.45 (C-6), 44.76 (C-5). HRMS: calcd. for $[C_{37}H_{35}N_3NaO_5]^+$ 602.26254; found 602.26238, calcd. for $[C_{37}H_{39}N_4O_5]^+$ 597.30715; found 597.30703.

(1*R*,2*S*,3*R*,4*R*,5*S*,6*S*)-2-Amino-4,5,6-tris(benzyloxy)-3-((benzyloxy)methyl)cyclohexan-1-ol (11)

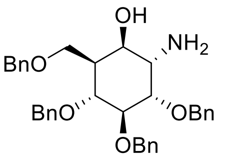

Azide **10** (0.23 g, 0.4 mmol) was dissolved in anhydrous THF (10 mL) and the solution was purged with N_2. PtO_2 (36 mg, 0.16 mmol, 0.4 eq) was added and the reaction mixture was purged again with N_2. The reaction was then stirred under H_2 atmosphere, for 4 h at rt. The reaction was flushed with N_2, filtered over a celite plug and concentrated *in vacuo*. The crude mixture was purified by silica gel column chromatography (Pentane/Acetone 100:1 → 40:60) to afford pure **11** (0.22 g, 0.39 mmol, 98 %). ^{1}H NMR (400 MHz, $CDCl_3$) δ 7.40 – 7.19 (m, 20H, CH Ar), 4.95 (d, J = 10.2 Hz, 2H, CH*H*Ph), 4.83 (d, J = 10.8 Hz, 1H, CH*H*Ph), 4.73 (d, J = 11.5 Hz, 1H, CH*H*Ph), 4.69 (d, J = 11.5 Hz, 1H, CH*H*Ph), 4.55 (d, J = 11.0 Hz, 1H, CH*H*Ph), 4.51 (d, J = 11.7 Hz, 1H, CH*H*Ph), 4.44 (d, J = 11.7 Hz, 1H, CH*H*Ph), 4.13 (dd, J = 9.1, 3.1 Hz, 1H, CH-7a), 4.05 (dd, J = 3.5, 2.1 Hz, 1H, CH-2), 4.04 – 3.90 (m, 3H, CH-1, CH-4, CH-6), 3.73 (dd, J = 9.1, 2.4 Hz, 1H, CH-7b), 3.50 (t, J = 3.6 Hz, 1H, CH-3), 2.30 – 2.23 (m, 1H, CH-5). ^{13}C NMR (101 MHz, $CDCl_3$) δ 139.2, 139.0, 138.9, 137.4 (4C_q Ar), 128.7, 128.5, 128.5, 128.5, 128.4, 128.1, 128.0, 127.9, 127.9, 127.8, 127.7, 127.6, 127.5 (20CH Ar), 83.8 (C-1/C-6), 80.7 (C-1/C-6), 77.2 (C-4), 75.6, 75.5 (2CH_2Ph), 75.4 (C-2), 73.8, 72.7 (2CH_2Ph), 70.7 (C-7), 52.7 (C-3), 40.9 (C-5). HRMS: calcd. for $[C_{35}H_{40}NO_5]^+$ 554.29010; found 554.29054.

***Tert*-butyl ((1*S*,2*S*,3*S*,4*R*,5*S*,6*R*)-2,3,4-tris(benzyloxy)-5-((benzyloxy)methyl)-6-hydroxycyclohexyl) carbamate (12).**

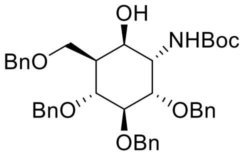

Amino alcohol **11** (120 mg, 0.21 mmol) was dissolved in anhydrous DCM (1.05 mL), and Et_3N (0.15 mL, 1.06 mmol, 5 eq) and Boc_2O (58 µL, 0.25 mmol, 1.2 eq) were added at 0 ℃. The reaction was stirred overnight at rt. The reaction was quenched with sat. aq. NH_4Cl. The aqueous layer was extracted with DCM (3x). The combined organic layers were dried over MgSO4, filtered and concentrated *in vacuo*. The crude product was purified by silica gel column chromatography (Pentane/EtOAc 9:1 → 7:3) to obtain **12** (113 mg, 0.17 mmol, 82%) as a colorless oil. ^1H NMR (400 MHz, $CDCl_3$) δ 7.39 – 7.28 (m, 18H, CH Ar), 7.24 – 7.21 (m, 2H, CH Ar), 4.99 (d, *J* = 10.8 Hz, 1H, CH*H*Ph), 4.95 (d, *J* = 10.8 Hz, 1H, CH*H*Ph), 4.83 – 4.76 (d, 1H, CH*H*Ph)), 4.70 (d, *J* = 11.1 Hz, 1H, CH*H*Ph)), 4.59 (d, *J* = 11.1 Hz, 1H, CH*H*Ph), 4.52 (d, *J* = 11.0 Hz, 1H, CH*H*Ph), 4.49 – 4.44 (m, 3H, 2CH*H*Ph, CH-6), 4.18 (m, 2H, CH-1, CH-2), 4.09 (dd, *J* = 9.1, 3.2 Hz, 1H, CH-7a), 4.04 (dd, *J* = 11.4, 8.9 Hz, 1H, CH-4), 3.80 (s, 1H, OH), 3.75 (dd, *J* = 9.1, 2.5 Hz, 1H, CH-7b), 3.59 (t, *J* = 9.1 Hz, 1H, CH-3), 1.92 (dd, *J* = 11.4, 2.5 Hz, 1H, CH-5), 1.47 (s, 9H, $(CH_3)_3$). ^{13}C NMR (101 MHz, $CDCl_3$) δ 156.0 (C=O), 138.9, 138.6, 138.1, 137.2 (4C_q Ar), 128.6, 128.4, 128.4, 128.4, 128.1, 128.1, 127.9, 127.8, 127.8, 127.8, 127.7, 127.5 (20CH Ar), 84.3, (C-3) 78.1 (C-1), 76.4 (C-4), 75.6, 75.5, 73.7 (3CH_2Ph), 72.1 (C-6), 71.9 (CH_2Ph), 70.1 (C-7), 51.8 (C-2), 41.2 (C-5), 28.4 ($(CH_3)_3$).). HRMS: calcd. for $[C_{40}H_{48}NO_7]^+$ 654.34253; found 654.34242 .

(1*R*,2*R*,3*R*,4*S*,5*S*,6*R*)-3,4,5-Tris(benzyloxy)-2-((benzyloxy)methyl)-6-((*tert*butoxycarbonyl) amino)cyclohexyl methanesulfonate (13).

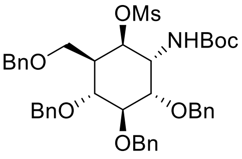

Intermediate **12** (146 mg, 0.22 mmol) was dissolved in anhydrous $CHCl_3$ (2.20 mL) and Et_3N (0.16 mL, 1.11 mmol, 5 eq), Me-imidazole (0.18 mL, 2.23 mmol, 10 eq) and MsCl (86 µL, 1.11 mmol, 5 eq) were added to the solution at 0 ℃. The reaction was stirred overnight at rt. The reaction mixture was diluted with EtOAc and the organic layer was washed with 1M HCl (3x), H_2O and brine. The washed organic layer was dried over $MgSO_4$, filtered and concentrated *in vacuo*. The crude product was purified by silica gel column chromatography (Pentane/EtoAc 9:1 → 7:3) to obtain **13** (146 mg, 0.19 mmol, 91%) as a yellow oil. ^1H NMR (500 MHz, $CDCl_3$) δ 7.34 – 7.24 (m, 18H, CH Ar), 7.18 – 7.14 (m, 2H ,CH Ar), 5.39 (m, 1H, CH-6), 4.91 (d, *J* = 10.8 Hz, 1H, CH*H*Ph), 4.85 – 4.81 (m, 2H, CH*H*Ph, NH), 4.74 (d, *J* = 10.8 Hz, 1H, CH*H*Ph), 4.64 (d, *J* = 11.3 Hz, 1H, CH*H*Ph), 4.59 (d, *J* = 11.3 Hz, 1H, CH*H*Ph), 4.46 (d, *J* = 12.7 Hz, 3H CH*H*Ph), 4.33 (s, 1H, CH-1), 4.00 (dd, *J* = 9.4, 4.7 Hz, 1H, CH-2), 3.78 (dd, *J* = 9.4, 4.3 Hz, 1H, CH-7a), 3.63 (t, *J* = 8.9 Hz, 1H, CH-3), 3.56 (t, *J* = 9.5 Hz, 1H, CH-4), 3.50 (s, 1H, CH-7b), 2.91 (s, 3H, CH_3), 2.42 (t, *J* = 8.4 Hz, 1H, CH-5), 1.44 (s, 9H, $(CH_3)_3$). ^{13}C NMR (126 MHz, $CDCl_3$) δ 155.7 (C=O), 138.6, 138.2, 137.9, 137.6 (4C_q Ar), 128.7, 128.6, 128.6, 128.4, 128.2, 128.0, 127.9, 127.9, 127.8 (CH Ar), 83.0 (C-3), 77.7 (C-6), 77.2 (C-4), 77.1 (C-2), 75.8, 75.4, 73.1, 72.4 (4CH_2Ph), 66.4 (C-7), 50.8 (C-1), 40.5 (C-5), 37.6 (CH_3), 28.4 ($(CH_3)_3$). HRMS: calcd. for $[C_{41}H_{49}NNaO_9S]^+$ 754.30202; found 754.30105, calcd. for $[C_{41}H_{53}N_2O_9S]^+$ 749.34663; found 749.34611 .

(3a*S*,4*S*,5*S*,6*R*,7*S*,7a*S*)-4,5,6-Tris(benzyloxy)-7-((benzyloxy)methyl)hexahydrobenzo[*d*]oxazol-2(3*H*)-one (14).

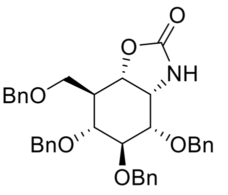

Intermediate **13** (140 mg, 0.19 mmol) was dissolved in anhydrous DMF (8.7 mL) and the reaction mixture was stirred for 24 h at 120 ℃. The reaction was then cooled to rt and diluted with H_2O. The aqueous phase was extracted with EtOAc (3x) and the combined organic layers were washed with H_2O (2x) and brine, dried over $MgSO_4$, filtered and concentrated *in vacuo*. The crude product was purified by silica gel column chromatography (Pent/EtOAc 100:1 → 50:50) to obtain **14** (70 mg, 0.12 mmol, 64%) as an orange oil. ^1H NMR (500 MHz, $CDCl_3$) δ 7.40 – 7.27 (m, 18H, CH Ar), 7.26 – 7.22 (m, 2H, CH Ar), 5.35 (s, 1H, NH), 4.83 (d, 1H, CH*H*Ph), 4.80 (dd, *J* = 7.1, 2.1 Hz, 1H, CH-6), 4.78 (d, *J* = 1.2 Hz, 3H, CH*H*Ph), 4.61 (d, *J* = 11.9 Hz, 1H, CH*H*Ph), 4.57 (d, *J* = 10.9 Hz, 1H, CH*H*Ph), 4.50 (d, *J* = 11.7 Hz, 1H, CH*H*Ph), 4.47 (d, *J* = 11.7 Hz, 1H, CH*H*Ph), 4.08 (dd, *J* = 7.4, 4.3 Hz, 1H, CH-1), 3.89 (dd, *J* = 9.3, 2.4 Hz, 1H, CH-7a), 3.86 (dd, *J* = 8.2, 7.3 Hz, 1H, CH-3), 3.65 (dd, *J* = 9.3, 2.2 Hz, 1H, CH-7b), 3.60 (dd, *J* = 7.3, 4.3 Hz, 1H, CH-2), 3.56 (dd, *J* = 11.6, 8.2 Hz, 1H, CH-4), 2.24 – 2.17 (m, 1H, CH-5). ^{13}C NMR (126 MHz, $CDCl_3$) δ 158.9 (C=O), 138.4, 138.3, 138.1, 137.7 (4C_q Ar), 128.8, 128.6, 128.6, 128.6, 128.3, 128.1, 128.0, 128.0, 127.9, 127.8 (20CH Ar), 82.5 (C-3), 77.4 (C-2), 76.2 (C-4), 75.1, 74.8 (2CH_2Ph), 74.2 (C-6), 73.6, 73.5 (2CH_2Ph), 65.5 (C-7), 54.5 (C-1), 44.6 (C-5). HRMS: calcd. for $[C_{36}H_{41}N_2O_6]^+$ 597.29591; found 597.29570, calcd. for $[C_{36}H_{37}NNaO_6]^+$ 602.25131; found 602.25088.

(1*S*,2*S*,3*S*,4*S*,5*R*,6*S*)-2-Amino-3,4,5-tris(benzyloxy)-6-((benzyloxy)methyl)cyclohexan-1-ol (15).

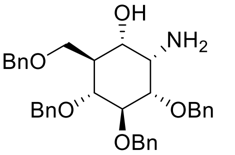

Cyclic carbamate **14** (70 mg, 120 µmol) was dissolved in EtOH (7.2 mL) and NaOH (1M, 1.8 mL, 1.8 mmol, 15 eq) was added to the solution. The reaction was stirred at 70 ℃ for 3 h and was subsequently stirred overnight at rt. The reaction mixture was concentrated and the crude residue was diluted with H_2O. The aqueous phase was extracted with EtOAc (3x) and the combined organic layers were washed with H_2O and brine, dried over $MgSO_4$, filtered and concentrated *in vacuo*. The crude product was purified by silica gel column chromatography (DCM/MeOH 100:1→ 80:20) to obtain **15** (64 mg, 115 µmol, 96%) as a colorless oil ^{1}H NMR (400 MHz, $CDCl_3$) δ 7.38 – 7.23 (m, 18H, CH Ar), 7.22 – 7.18 (m, 2H, CH Ar), 4.93 (d, *J* = 10.7 Hz, 1H, CH*H*Ph), 4.87 (d, *J* = 10.9 Hz, 1H, CH*H*Ph)), 4.79 (d, *J* = 10.8 Hz, 1H, CH*H*Ph), 4.67 (s, 2H, CH*H*Ph)), 4.50 (d, *J* = 11.6 Hz, 2H, CH*H*Ph), 4.45 (d, *J* = 12.0 Hz, 2H, CH-6), 3.98 (t, *J* = 9.3 Hz, 1H, CH-3), 3.85 (dd, *J* = 9.0, 2.8 Hz, 1H, CH-7a), 3.69 – 3.60 (m, 2H, CH-6, CH-7b), 3.52 (t, *J* = 3.7 Hz, 1H, CH-1), 3.43 (dd, *J* = 9.6, 3.7 Hz, 1H, CH-2), 3.35 (dd, *J* = 11.0, 9.1 Hz, 1H, CH-4), 2.32 – 2.05 (m, 4H, CH-5, NH_2, OH). ^{13}C NMR (101 MHz, $CDCl_3$) δ 139.0, 138.7, 138.5, 138.1 (4C_q Ar), 128.6, 128.6, 128.5, 128.5, 128.1, 128.0, 127.9, 127.8, 127.7, 127.6 (20CH Ar), 82.8 (C-3), 80.7 (C-2), 75.7, 75.2, 73.4, 72.4 (4CH_2Ph), 69.9 (C-6), 68.7 (C-7), 52.8 (C-1), 42.9 (C-5). HRMS: calcd. for $[C_{35}H_{40}NO_5]^+$ 554.29010; found 554.28970.

***Tert*-butyl (3a*R*,4*S*,5*S*,6*R*,7*R*,7a*S*)-4,5,6-tris(benzyloxy)-7-((benzyloxy)methyl)hexahydro-3*H*-benzo[*d*][1,2,3]oxathiazole-3-carboxylate 2,2-dioxide (16).** The amino alcohol **15** (63 mg, 0.12 mmol) was

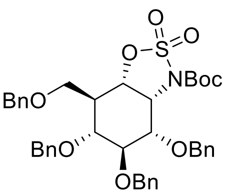

dissolved in anhydrous DCM (2.22 mL). Et_3N (84 µL, 0.6 mmol, 5 eq) and Boc_2O (32µL, 0.14 mmol, 1.2 eq) were added to the solution at 0 ℃ and the reaction was stirred overnight at rt. The reaction was quenched with sat. aq. NH_4Cl and the aqueous phase was extracted with DCM (3x). The combined organic layers were dried over $MgSO_4$, filtered and concentrated *in vacuo*. The Boc protected intermediate was dissolved in anhydrous DCM (2.22 mL) and Et_3N (0.18 mL, 1.26 mmol, 10.5 eq), imidazole (45 mg, 0.66 mmol, 5.5 eq) and $SOCl_2$ (88 µL, 1.2 mmol, 10 eq) were added at 0 ℃. The reaction was stirred at this temperature for 20 min. H_2O was added to the reaction mixture and the aqueous phase was extracted with DCM (3x). The combined organic layers were dried over $MgSO_4$, filtered and concentrated *in vacuo*. The crude mixture of sulfites was dissolved in a 1:1:1 mixture of H_2O (0.6 mL), EtOAc (0.6 mL) and MeCN (0.6 mL), and $NaIO_4$ (64 mg, 0.30 mmol, 2.5 eq) and $RuCl_3{\cdot}H_2O$ (6.2 mg, 30 µmol, 0.25 eq) were added at 0 ℃ and the reaction was stirred at this temperature for 1 h. The reaction was quenched with sat. aq. $Na_2S_2O_3$ and the aqueous phase was extracted with EtOAc (3x). The combined organic layers were washed with brine, dried over $MgSO_4$, filtered and concentrated *in vacuo*. The crude product was purified by silica gel column chromatography (Pentane/EtOAc 100:1 → 90:10) to obtain **16** (66 mg, 92 µmol, 77% over 3 steps) as a colorless oil. ^{1}H NMR (500 MHz, $CDCl_3$) δ 7.40 – 7.23 (m, 18H, CH Ar), 7.18 – 7.15 (m, 2H, CH Ar), 5.24 (t, *J* = 8.4, 7.3, 0.9 Hz, 1H, CH-6), 4.65 (d, *J* = 12.2 Hz, 1H, CH*H*Ph), 4.61 (d, *J* = 11.1 Hz, 1H, CH*H*Ph), 4.58 – 4.50 (m, 3H, CH*H*Ph), 4.48 – 4.42 (m, 2H, CH*H*Ph), 4.33 – 4.27 (m, 2H, CH*H*Ph, CH-1), 4.10 (s, 1H, CH-2), 3.87 (dd, *J* = 9.4, 2.4 Hz, 1H, CH-7a), 3.82 (dd, *J* = 5.6, 1.4 Hz, 1H, CH-3), 3.64 – 3.58 (m, 2H, CH-4, CH-7b), 2.73 (ddt, *J* = 13.0, 8.8, 2.3 Hz, 1H, CH-5), 1.51 (s, 9H, $(CH_3)_3$). ^{13}C NMR (126 MHz, $CDCl_3$) δ 148.7 (C=O), 138.2, 138.0, 137.4, 137.4 (4C_q Ar), 128.7, 128.7, 128.6, 128.5, 128.5, 128.3, 128.2, 128.1, 127.9, 127.9, 127.8 (20CH Ar), 82.6 (C-3), 76.2 (C-4) , 76.1 (C-6) , 74.0 (CH_2Ph), 73.6 (C-2), 73.5, 73.0, 71.6 (3CH_2Ph), 64.9 (C-7), 56.8 (C-1), 41.1 (C-5), 28.1 ($(CH_3)_3$). HRMS: calcd. for $[C_{40}H_{45}NO_9SNa]^+$ 738.27072; found 738.27009, calcd. for $[C_{40}H_{49}N_2O_9S]^+$ 733.31533; found 733.31478.

(3a*R*,4*S*,5*S*,6*R*,7*R*,7a*S*)-4,5,6-Tris(benzyloxy)-7-((benzyloxy)methyl)hexahydro-3*H*-benzo[*d*][1,2,3]oxathiazole 2,2-dioxide (17).

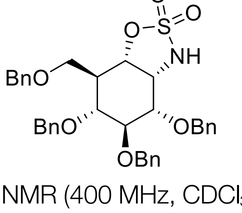

Cyclosulfamidate **16** (60 mg, 84 µmol) was dissolved in anhydrous DCM (4 mL) and TFA (0.38 mL) was added to the reaction mixture. The reaction was stirred overnight at rt. The reaction mixture was diluted with H_2O and the aqueous phase was extracted with DCM (3x). The combined organic layers were dried over $MgSO_4$, filtered and concentrated *in vacuo*. The crude product was purified by silica gel column chromatography (Pentane/EtOAc 95:5 → 80:20) to obtain **17** (32 mg, 52 µmol, 62%) as a colorless oil. ^{1}H NMR (400 MHz, $CDCl_3$) δ 7.36 – 7.23 (m, 18H, CH Ar), 7.21 (m, 2H, CH Ar), 4.96 (dd, *J* = 10.9, 6.5 Hz, 1H, CH-6), 4.86 (d, *J* = 7.1 Hz, 1H, NH), 4.67 (m, *J* = 23.4, 11.7 Hz, 4H, CH*H*Ph), 4.52 (d, *J* = 11.5 Hz, 1H, CH*H*Ph), 4.47 (d, *J* = 11.8 Hz, 1H, CH*H*Ph), 4.43 (d, *J* = 11.1 Hz, 1H, CH*H*Ph), 4.38 (d, *J* = 11.8 Hz, 1H, CH*H*Ph), 4.28 (td, *J* = 6.8, 4.7 Hz, 1H, CH-1), 3.92 (t, 1H, CH-3), 3.80 (dd, *J* = 6.5, 4.8 Hz, 1H, CH-2), 3.77 (dd, *J* = 9.4, 2.1 Hz, 1H, CH-7a), 3.65 (dd, *J* = 10.8, 5.4 Hz, 1H, CH-4), 3.59 (dd, *J* = 9.5, 2.2 Hz, 1H, CH-7b), 2.46 (tt, *J* = 10.8, 2.0 Hz, 1H, CH-5). ^{13}C NMR (101 MHz, $CDCl_3$) δ 138.1, 137.8, 136.8 (4C_q Ar), 128.9, 128.7, 128.6, 128.6, 128.3, 128.2, 128.0, 127.9, 127.9 (20CH Ar), 79.7 (C-6), 78.7 (C-3), 76.0 (C-4), 74.8 (C-2), 74.1, 73.9, 73.3,

73.3 ($4CH_2Ph$), 64.6 (C-7), 55.7 (C-1), 43.1 (C-5). HRMS: calcd. for $[C_{40}H_{45}NO_9SNa]^+$ 638.27072; found 638.27000, calcd. for $[C_{40}H_{49}N_2O_9S]^+$ 633.31533; found 633.31472.

(3a*R*,4*S*,5*S*,6*R*,7*R*,7a*S*)-4,5,6-Tris(benzyloxy)-7-((benzyloxy)methyl)-3-methylhexahydro-3*H*-benzo[*d*][1,2,3]oxathiazole 2,2-dioxide (18).

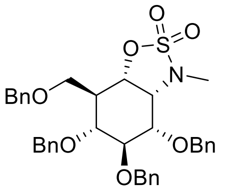

K_2CO_3 (4.1 mg, 30 µmol 1.2 eq), TBAI (1 mg 0.1 eq) and iodomethane (3.12 µL, 49 µmol, 2 eq) were added to a solution of **17** (15 mg, 24 µmol) in anhydrous DMF (1 mL) and the reaction was stirred at rt for 5 h. The reaction mixture was diluted with EtOAc and H_2O and the aqueous phase was extracted with EtOAc (3x). The combined organic layers were washed with water and brine, dried over $MgSO_4$, filtered and concentrated *in vacuo*. The crude product was purified by silica gel column chromatography (Pent/EtOAc 100:1 → 85:15) to afford **18** (10 mg, 16 µmol, 65%) as a purple oil. ^{1}H NMR (500 MHz, $CDCl_3$) δ 7.37 – 7.25 (m, 18H, CH Ar), 7.21 – 7.18 (m, 2H, CH Ar), 4.88 (dd, J = 10.0, 5.7 Hz, 1H, CH-6), 4.84 (d, J = 11.0 Hz, 1H, CH*H*Ph), 4.76 (d, J = 12.2 Hz, 3H, CH*H*Ph), 4.66 (d, J = 11.7 Hz, 1H, CH*H*Ph), 4.51 (d, J = 11.0 Hz, 1H, CH*H*Ph), 4.46 (d, J = 11.7 Hz, 1H, CH*H*Ph), 4.40 (d, J = 11.7 Hz, 1H, CH*H*Ph), 3.95 (t, J = 8.3 Hz, 1H, CH-3), 3.85 – 3.81 (m, 2H, CH-1, CH-7a), 3.61 – 3.54 (m, 3H, CH-2, CH-4, CH-7b), 2.90 (s, 3H, C*H*$_3$), 2.56 (ddt, J = 11.8, 9.9, 2.0 Hz, 1H, CH-5). ^{13}C NMR (126 MHz, $CDCl_3$) δ 138.4, 138.2, 138.0, 137.4 (4C$_q$ Ar), 128.8, 128.6, 128.6, 128.3, 128.1, 128.1, 128.0, 128.0, 127.9, 127.8 (20CH Ar), 81.9 (C-3), 77.6 (C-2/C-4), 77.3 (C-6), 76.4 (C-2/C-4), 75.2, 75.1, 74.3, 73.5 (4CH_2Ph), 64.5 (C-7), 61.3 (C-1), 44.1 (C-5), 33.4 (CH_3). HRMS: calcd. for $[C_{36}H_{39}NO_7SNa]^+$ 652.23394; found 652.23382.

(3Ar,4*S*,5*S*,6*R*,7*R*,7a*S*)-4,5,6-Trihydroxy-7-(hydroxymethyl)hexahydro-3*H*-benzo[*d*][1,2,3]oxathiazole 2,2-dioxide (4).

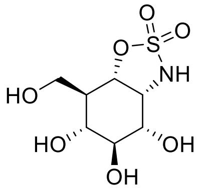

Perbenzylated **17** (21 mg, 31 µmol) was dissolved in MeOH (1.16 mL), purged with N_2 and Pd/C (10 wt %, 13 mg, 12 µmol, 0.4 eq) was added and the reaction mixture was purged again with N_2. The reaction was then stirred overnight under H_2 atmosphere at rt. The reaction was flushed with N_2, filtered over a celite plug and concentrated *in vacuo*. The crude product was purified by silica gel column chromatography (DCM/MeOH 100:1 → 80:20) to obtain **4** (8.23 mg, 31 µmol, 99%) as a clear oil. ^{1}H NMR (400 MHz, MeOD) δ 4.93 – 4.90 (1H, CH-6), 4.37 (t, J = 4.8 Hz, 1H, CH-1), 4.01 (dd, J = 11.2, 2.3 Hz, 1H, CH-7a), 3.70 – 3.62 (m, 2H, CH-3, CH-7b), 3.59 (dd, J = 9.5, 4.3 Hz, 1H, CH-2), 3.36 – 3.33 (m, 1H, CH-4), 2.17 – 2.09 (m, 1H, CH-5). ^{13}C NMR (101 MHz, MeOD) δ 81.7 (C-6), 74.9 (C-3), 71.0 (C-2), 69.3 (C-4), 61.0 (C-1), 57.6 (C-7), 46.6 (C-5). HRMS: calcd. for $[C_7H_{14}N_1O_7S]^+$ 256,04855; found 256,04812.

(3a*R*,4*S*,5*S*,6*R*,7*R*,7a*S*)-4,5,6-Trihydroxy-7-(hydroxymethyl)-3-methylhexahydro-3*H*-benzo[*d*][1,2,3]oxathiazole 2,2-dioxide (5).

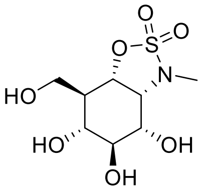

Perbenzylated **18** (10 mg, 16 µmol) was dissolved in a 4:1 mixture of MeOH (1 mL) and DCM (0.25 mL), purged with N_2 and Pd/C (10 wt %, 6.8 mg, 6.4 µmol, 0.4 eq) was added and the reaction mixture was purged again with N_2. The reaction was stirred overnight under H_2 atmosphere at rt. The reaction mixture was flushed with N_2, filtered over a celite plug and concentrated *in vacuo*. The crude product was purified by silica gel column chromatography (DCM/MeOH 100:1 → 80:20) to obtain **5** (2.3 mg, 8.8 µmol, 55%) as a white solid. ^{1}H NMR (500 MHz, MeOD) δ 4.89 (dd, J = 10.2, 5.3 Hz, 1H, CH-6), 3.98 (dd, J = 11.2, 2.3 Hz, 1H, CH-7a), 3.93 (dd, J = 5.3, 3.4 Hz, 1H, CH-1), 3.67 (dd, J = 8.5, 2.7 Hz, 1H, CH-7b), 3.66 – 3.63 (m, 1H, CH-3), 3.59 (dd, J = 9.8, 3.4 Hz, 1H, CH-2), 3.36 (dd, J = 11.2, 8.7 Hz, 1H, CH-4), 2.93 (s, 3H, CH_3), 2.19 (tt, J = 10.3, 2.5 Hz, 1H, CH-5). ^{13}C NMR (126 MHz, MeOD) δ 79.4 (C-6), 74.7 (C-3), 72.1 (C-2), 69.7 (C-4), 64.8 (C-1), 57.5 (C-7), 47.5 (C-5), 34.5 (CH_3). HRMS: calcd. for $[C_8H_{16}NO_7S]^+$ 270.06420; found 270.06408.

3.2.2 Synthesis and characterization data of 6 and 7

(1*R*,2*S*,3*S*,4*S*,5*R*,6*S*)-2-amino-3,4,5-tris(benzyloxy)-6-((benzyloxy)methyl)cyclohexan-1-ol (20)

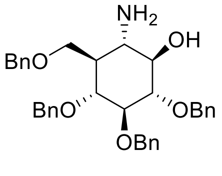

Azide **19** (0.21 g, 0.36 mmol) was dissolved in anhydrous THF (10 mL) and the solution was purged with N_2. PtO_2 (33 mg, 0.14 mmol, 0.4 eq) was added and the reaction mixture was purged again with N_2. The reaction was then stirred under H_2 atmosphere, for 4 h at rt. The reaction was flushed with N_2, filtered over a celite plug and concentrated *in vacuo*. The crude mixture was purified by silica gel column chromatography (Pentane/Acetone 100:1 → 40:60) to afford pure **20** (0.19 g, 0.35 mmol, 97 %). ^{1}H NMR (400 MHz, $CDCl_3$) δ 7.36 – 7.24 (m, 18H, CH Ar), 7.22 – 7.19 (m, 2H, CH Ar), 4.94 (d, J = 11.2 Hz, 1H, CH*H*Ph), 4.91 – 4.86 (m, 3H, CH*H*Ph), 4.79 (d, J = 11.3 Hz, 1H, CH*H*Ph), 4.53 (d, J = 10.8 Hz, 1H, CH*H*Ph), 4.45 (d, J = 12.0 Hz, 1H, CH*H*Ph), 4.41 (d, J = 12.0 Hz, 1H, CH*H*Ph), 3.76 (dd, J = 9.3, 2.4 Hz, 1H, CH-7a), 3.68 – 3.62 (m, 2H, CH-6, CH-7b), 3.57 (t, J = 9.2 Hz, 1H, CH-1), 3.38 (t, J = 9.2 Hz, 1H, CH-2), 3.24 (t, J = 9.5 Hz, 1H, CH-3), 2.79 (t, J = 10.4 Hz, 1H, CH-4), 2.13 (s, 3H, OH, NH_2), 1.41 (tt, J = 10.9, 2.5 Hz, 1H, CH-5). ^{13}C NMR (101 MHz, $CDCl_3$) δ 140.4, 140.3, 140.2, 139.9 (4C$_q$ Ar), 130.3, 130.1, 130.1, 129.7, 129.6, 129.5, 129.4, 129.4, 129.3 (20CH

Ar), 87.8 (C-1), 85.5 (C-2), 80.3 (C-6), 78.3 (C-3), 77.3, 77.2, 77.1, 74.7 (4CH_2Ph), 66.6 (C-7), 52.6 (C-4), 48.40 (C-5). HRMS: calcd. for $[C_{35}H_{40}NO_5]^+$ 554.29010; found 554.28976.

***Tert*-butyl ((1*S*,2*R*,3*R*,4*S*,5*S*,6*R*)-3,4,5-tris(benzyloxy)-2-((benzyloxy)methyl)-6 hydroxycyclohexyl) carbamate (21).**

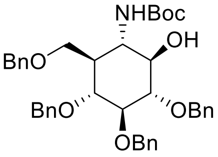

Amino alcohol **20** (74 mg, 0.13 mmol) was dissolved in anhydrous DCM (2.6 mL) and Et_3N (93 µL, 0.7 mmol, 5 eq) and Boc_2O (37 µL, 0.16 mmol, 1.2 eq) were added at 0 °C. The reaction was stirred overnight at rt. Then, the reaction was quenched with sat. aq. NH_4Cl and the aqueous phase was extracted with DCM (3x). The combined organic layers were dried over $MgSO_4$, filtered and concentrated *in vacuo*. The crude product was purified by silica gel column chromatography (Pentane/EtOAc 100:1 → 6:4) to obtain **21** (66 mg, 0.1 mmol, 78%) as a colorless oil. ^{1}H NMR (500 MHz, DMSO-d_6) δ 7.36 – 7.22 (m, 20H, CH Ar), 5.33 (dd, *J* = 11.9, 4.9 Hz, 1H, CH*H*Ph), 4.67 (d, *J* = 11.9 Hz, 1H, CH*H*Ph), 4.62 (d, *J* = 12.1 Hz, 1H, CH*H*Ph), 4.56 (td, *J* = 12.6, 11.9, 2.6 Hz, 4H, CH*H*Ph), 4.44 (d, *J* = 1.1 Hz, 2H, CH*H*Ph), 4.41 – 4.38 (m, 1H, OH), 3.99 (dd, *J* = 10.6, 2.9 Hz, 1H, CH-4), 3.95 (dd, *J* = 5.5, 2.9 Hz, 1H, CH-3), 3.92 (t, *J* = 3.3 Hz, 1H, CH-7b), 3.82 (dd, *J* = 11.9, 2.6 Hz, 1H, CH-6), 3.78 (dd, *J* = 10.7, 6.6 Hz, 1H, CH-7a), 3.15 (q, *J* = 3.7, 2.5 Hz, 1H, CH-5), 1.46 (s, 9H, $(CH_3)_3$). ^{13}C NMR (126 MHz, DMSO) δ 128.8, 128.7, 128.7, 128.5, 128.1, 128.00, 127.9, 127.9, 127.9, 127.8, 127.7 (CH Ar), 83.78 (C-3), 81.79 (*C*$(CH_3)_3$), 77.0, 75.6, 73.5, 73.0, 72.9, 72.6 (4CH_2Ph), 71.2 (C-6), 65.4 (C-7), 57.7 (C-1), 41.8 (C-5), 28.4 ($(CH_3)_3$). HRMS: calcd. for $[C_{40}H_{47}NO_7Na]^+$ 676.32447; found 654.32409.

(1*R*,2*R*,3*S*,4*R*,5*R*,6*S*)-2,3,4-Tris(benzyloxy)-5-((benzyloxy)methyl)-6-((*tert*-butoxycarbonyl)amino) cyclohexyl ethanesulfonate (22).

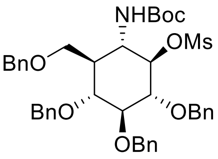

Intermediate **21** (40 mg, 61 µmol) was dissolved in anhydrous DCM (0.6 mL) and Et_3N (42 µL, 0.3 mmol, 5 eq), Me-imidazole (50 µL, 0.61 mmol, 10 eq) and MsCl (23 µL, 0.30 mmol, 5 eq) were added to the reaction at 0 °C. The reaction was stirred at rt overnight. The reaction was diluted with EtOAc and washed with 1M HCl (3x), H_2O and brine. The organic layer was dried over $MgSO_4$, filtered and concentrated *in vacuo*. The crude product was purified by silica gel column chromatography (Pentane/EtOAc 100:1 → 7:3) to obtain **22** (35,5 mg, 49 µmol, 80%) as a yellow oil and **32** as a white solid side product (4,5 mg, 8 µmol, 13%). ^{1}H NMR (400 MHz, $CDCl_3$) *δ* 7.41 – 7.25 (m, 18H, CH Ar), 7.24 – 7.20 (m, 2H, CH Ar), 4.97 (d, *J* = 10.9 Hz, 1H, CH*H*Ph), 4.94 – 4.87 (m, 3H, CH*H*Ph), 4.81 (d, *J* = 10.9 Hz, 1H, CH*H*Ph), 4.68 (d, *J* = 9.4 Hz, 1H, CH*H*Ph), 4.61 (d, *J* = 10.7 Hz, 2H, CH*H*Ph, CH-1), 4.51 (d, *J* = 11.5 Hz, 1H, CH*H*Ph), 4.41 (d, *J* = 11.5 Hz, 1H, CH*H*Ph), 3.93 (dd, 1H, CH-6), 3.81 (dd, *J* = 9.3, 2.6 Hz, 1H, CH-7a), 3.76 (ddd, *J* = 10.3, 6.5, 2.7 Hz, 1H, CH-4), 3.67 – 3.60 (m, 2H, CH-3, CH-7b), 3.55 (dd, *J* = 9.4, 2.1 Hz, 1H, CH-2), 2.89 (s, 3H, CH_3), 1.88 – 1.76 (m, 1H, CH-5), 1.48 (s, 9H, $(CH_3)_3$). ^{13}C NMR (101 MHz, $CDCl_3$) *δ* 155.2 (C=O), 138.3, 138.2, 138.1, 137.8 (4C_q Ar), 128.5, 128.5, 128.4, 128.0, 127.9, 127.8, 127.7, 127.7, 127.7 (20CH Ar), 85.6 (C-2/C-3), 83.8 (C-1), 80.8 (C-2/C-3), 77.8 (C-4), 75.7, 75.6, 75.5, 73.5 (4CH_2Ph), 64.9 (C-7), 50.0 (C-6), 44.2 (C-5), 38.8 (CH_3), 28.4 ($(CH_3)_3$). HRMS: calcd. for $[C_{41}H_{49}NNaO_9S]^+$ 754.30202; found 754.30184, calcd. for $[C_{41}H_{53}N_2O_9S]^+$ 749.34663; found 749.34652.

(3a*S*,4*R*,5*R*,6*S*,7*S*,7a*R*)-5,6,7-tris(benzyloxy)-4-((benzyloxy)methyl)hexahydrobenzo[d]oxazol-2(3*H*)-one (32).

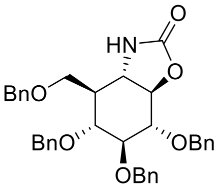

^{1}H NMR (400 MHz, $CDCl_3$) δ 7.39 – 7.22 (m, 18H, CH_{Ar}), 7.20 – 7.15 (m, 2H, CH_{Ar}), 5.20 (s, 1H, NH), 4.95 (dd, *J* = 11.1, 5.3 Hz, 2H, 2x CH*H*Ph), 4.81 (d, *J* = 11.0 Hz, 1H, CH*H*Ph), 4.74 (d, *J* = 10.8 Hz, 1H, CH*H*Ph), 4.70 (d, *J* = 11.4 Hz, 1H, CH*H*Ph), 4.45 (d, *J* = 11.0 Hz, 1H, CH*H*Ph), 4.40 (s, 2H, 2x CH*H*Ph), 4.10 (dd, *J* = 11.7, 9.8 Hz, 1H, C-3), 3.86 (dd, *J* = 9.9, 7.6 Hz, 1H, CH-1), 3.72 (t, *J* = 8.0 Hz, 1H, CH-2), 3.58 (dd, *J* = 9.5, 3.8 Hz, 2H, CH-7), 3.49 (dd, *J* = 9.5, 8.4 Hz, 1H, CH-4), 3.44 (dd, *J* = 9.5, 6.6 Hz, 1H, CH-7), 3.40 – 3.33 (m, 1H, CH-6), 2.01 (tdd, *J* = 13.2, 6.6, 3.8 Hz, 1H, CH-5). ^{13}C NMR (101 MHz, $CDCl_3$) δ 160.0 ($C_{q\ carbamate}$), 138.2, 137.9, 137.9, 137.5 (4C_q), 128.8, 128.6, 128.58, 128.5, 128.3, 128.2, 128.1, 128.08, 128.0, 127.97, 127.9 (11CH_{Ar}), 85.9 (C-2), 83.6 (C-3), 80.1 (C-1), 79.6 (C-4), 76.0, 75.4, 73.5 (4CH_2Ph), 68.7 (C-7), 56.1 (C-6), 43.5 (C-5). HRMS: calcd. for $[C_{36}H_{41}N_2O_6]^+$ 597.29591; found 597.29572, calcd. for $[C_{36}H_{37}NNaO_6]^+$ 602.25131; found 602.25126.

3a*S*,4*R*,5*R*,6*S*,7*S*,7a*S*)-5,6,7-Tris(benzyloxy)-4-((benzyloxy)methyl)hexahydrobenzo[*d*]oxazol-2(3*H*)-one (23).

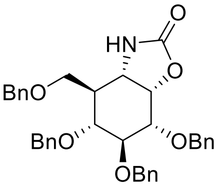

Intermediate **22** (89 mg, 0.12 mmol) was dissolved in anhydrous DMF (5.6 mL). The reaction mixture was stirred for 24 h at 120 °C. The reaction was cooled to rt and diluted with H_2O. The aqueous phase was extracted with EtOAc (3x) and the combined organic layers were washed with H_2O (2x) and brine, dried over $MgSO_4$, filtered and concentrated *in vacuo*. The crude product was purified by silica gel column chromatography (Pent/EtOAc 100:1 → 50:50) to obtain **23** (60 mg, 0.11 mmol, 85%) as a white solid. ^{1}H NMR (400 MHz, $CDCl_3$) *δ* 7.41 – 7.24 (m, 16H, CH Ar), 7.24 – 7.20 (m, 2H CH Ar), 7.20

– 7.14 (m, 2H, CH Ar), 5.67 (s, 1H, NH), 4.79 (d, *J* = 12.0 Hz, 1H, CH*H*Ph), 4.71 (dd, *J* = 8.7, 3.7 Hz, 1H, CH-1), 4.64 (d, *J* = 12.1 Hz, 1H, CH*H*Ph), 4.60 (d, *J* = 11.5 Hz, 1H, CH*H*Ph), 4.48 (dd, *J* = 11.6, 4.4 Hz, 2H, CH*H*Ph), 4.43 – 4.34 (m, 3H, CH*H*Ph), 3.87 (t, *J* = 3.7 Hz, 1H, CH-2), 3.82 – 3.75 (m, 3H, CH-3, CH-6, CH-7a), 3.29 (t, *J* = 9.0 Hz, 1H, CH-4), 3.24 (dd, *J* = 12.0, 5.5 Hz, 1H, CH-7b), 2.61 (dtd, *J* = 12.8, 9.1, 3.8 Hz, 1H, CH-5). ^{13}C NMR (101 MHz, $CDCl_3$) δ 158.7 (C=O), 138.0, 137.8, 137.7, 137.6 (4C$_q$ Ar), 128.7, 128.7, 128.6, 128.5, 128.3, 128.2, 128.1, 128.1, 128.0, 127.9 (20CH Ar), 81.9 (C-3/C-6), 78.3 (C-4), 75.7 (C-2), 74.8 (C-1), 73.7, 73.0, 72.6 (3CH_2Ph), 71.5 (C-7), 54.4 (C-3/C-6), 42.5 (C-5). HRMS: calcd. for $[C_{36}H_{41}N_2O_6]^+$ 597.29591; found 597.29572, calcd. for $[C_{36}H_{37}NNaO_6]^+$ 602.25131; found 602.25110.

(1*S*,2*S*,3*R*,4*R*,5*S*,6*S*)-2-Amino-4,5,6-tris(benzyloxy)-3-((benzyloxy)methyl)cyclohexan-1-ol (24).

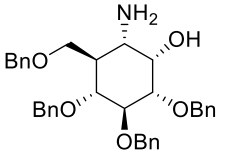

Cyclic carbamate **23** (60 mg, 0.11 mmol) was dissolved in EtOH (6.6 mL) and NaOH (1M, 1.65 mL, 1.65 mmol, 15 eq) was added to the solution. The reaction mixture was stirred at 70 ℃ for 3 h and was subsequently stirred overnight at rt. After 24 h extra NaOH (1M, 1.65 mL, 1.65 mmol, 15 eq) was added and the reaction was again heated to 70 ℃ and stirred for 3 h followed by overnight stirring at rt.. The reaction mixture was concentrated and the crude residue was diluted with H_2O. The aqueous phase was extracted with EtOAc (3x) and the combined organic layers were washed with H_2O and brine, dried over $MgSO_4$, filtered and concentrated *in vacuo*. The crude product was purified by silica gel column chromatography (DCM/MeOH 100:1 → 85:15) to obtain **24** (50 mg, 90 µmol, 82%) as a colorless oil. ^{1}H NMR (500 MHz, $CDCl_3$) δ 7.37 – 7.24 (m, 18H, CH Ar), 7.22 – 7.18 (m, 2H, CH Ar), 4.91 (d, *J* = 10.8 Hz, 1H, CH*H*Ph), 4.88 (d, *J* = 10.9 Hz, 1H, CH*H*Ph), 4.81 (d, *J* = 10.8 Hz, 1H, CH*H*Ph), 4.71 (d, 1H, CH*H*Ph), 4.70 (d, 1H, CH*H*Ph), 4.48 (d, *J* = 10.9 Hz, 1H, CH*H*Ph), 4.43 (s, 2H, CH*H*Ph), 4.12 (t, *J* = 2.7 Hz, 1H, CH-1), 3.90 (t, *J* = 9.4 Hz, 1H, CH-3), 3.75 (dd, *J* = 9.5, 3.2 Hz, 1H, CH-7a), 3.67 (dd, *J* = 9.5, 2.6 Hz, 1H, CH-7b), 3.49 (dd, *J* = 10.8, 9.3 Hz, 1H, CH-4), 3.43 (dd, *J* = 9.5, 2.7 Hz, 1H, CH-2), 2.92 (dd, *J* = 10.9, 2.5 Hz, 1H, CH-6), 2.64 (s, 3H, NH_2, OH), 1.97 (tt, *J* = 11.0, 2.9 Hz, 1H, CH-5). ^{13}C NMR (126 MHz, $CDCl_3$) δ 139.0, 138.7, 138.3, 138.1 (4C$_q$ Ar), 128.6, 128.6, 128.5, 128.5, 128.1, 128.1, 128.0, 127.9, 127.7, 127.6 (20CH Ar), 83.3 (C-3), 81.3 (C-2), 78.7 (C-4), 75.8, 75.5, 73.2, 72.6 (4CH_2Ph), 70.9 (C-1), 66.3 (C-7), 49.9 (C-6), 43.4 (C-5). HRMS: calcd. for $[C_{35}H_{40}NO_5]^+$ 554.29010; found 554.28970. \

***Tert*-butyl (3a*S*,4*R*,5*R*,6*S*,7*R*,7a*S*)-5,6,7-tris(benzyloxy)-4-((benzyloxy)methyl)hexahydro-3*H*-benzo[*d*][1,2,3]oxathiazole-3-carboxylate 2,2-dioxide (25).**

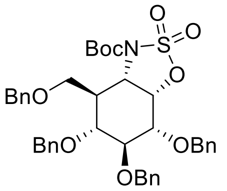

The amino alcohol **24** (50 mg, 90 µmol) was dissolved in anhydrous DCM (1.67 mL). Et_3N (63 µL, 0.45 mmol, 5 eq) and Boc_2O (25 µL, 0.11 mmol, 1.2 eq) were added at 0 ℃ and the reaction was stirred overnight at rt. The reaction was then quenched with sat. aq. NH_4Cl and the aqueous phase was extracted with DCM (3x). The combined organic layers were dried over $MgSO_4$, filtered and concentrated *in vacuo*. The Boc proctected intermediate was dissolved in anhydrous DCM (1.67 mL) and Et_3N (132 µL, 0.95 mmol, 10.5 eq), imidazole (34 mg, 0.50 mmol, 5.5 eq) and $SOCl_2$ (66µL, 0.9 mmol, 10 eq) were added at 0 ℃ and the reaction was stirred at rt for 20 min. H_2O was added to the reaction mixture and the aqueous phase was extracted with DCM (3x). The combined organic layers were dried over $MgSO_4$, filtered and concentrated *in vacuo*. The crude mixture of sulfites was dissolved in a 1:1:1 mixture of H_2O (1.3 mL), EtOAc (1.3 mL) and MeCN (1.3 mL), and $NaIO_4$ (48 mg, 0.23 mmol, 2.5 eq) and $RuCl_3{\cdot}H_2O$ (4.7 mg, 23 µmol, 0.25 eq) were added at 0 ℃ and the reaction was stirred at this temperature for 1h. The reaction was quenched with sat. aq. $Na_2S_2O_3$ and the aqueous phase was extracted with EtOAc (3x). The combined organic layers were washed with brine, dried over $MgSO_4$, filtered and concentrated *in vacuo*. The crude product was purified by silica gel column chromatography (Pentane/EtOAc 100:1 → 90:10) to obtain **25** (21 mg, 30 µmol, 33% over 3 steps) as a colorless oil. ^{1}H NMR (400 MHz, $CDCl_3$) δ 7.37 – 7.23 (m, 18H, CH Ar), 7.19 – 7.14 (m, 2H, CH Ar), 4.99 (t, *J* = 3.6 Hz, 1H, CH-1), 4.92 (d, *J* = 10.7 Hz, 1H, CH*H*Ph), 4.91 – 4.84 (m, 2H, CH*H*Ph), 4.79 (d, *J* = 11.9 Hz, 1H, CH*H*Ph), 4.74 (d, *J* = 11.9 Hz, 1H, CH*H*Ph), 4.66 (dd, *J* = 10.4, 3.9 Hz, 1H, CH-6), δ 4.52 (d, *J* = 11.3 Hz, 1H, CH*H*Ph), 4.35 (d, *J* = 11.3 Hz, 1H, CH*H*Ph), 3.94 (t, 1H, CH-3), 3.91 – 3.88 (m, 1H, CH-7a), 3.63 (dd, *J* = 7.0, 2.9 Hz, 1H, CH-4), 3.59 (dd, *J* = 1.8 Hz 1H, CH-2), 3.34 (dd, *J* = 9.5, 1.9 Hz, 1H, CH-7b), 2.22 (tt, 1H, CH-5), 1.55 (s, 9H $(CH_3)_3$). ^{13}C NMR (101 MHz, $CDCl_3$) δ 149.0 (C=O), 138.5, 138.4, 138.2, 137.2 (4C$_q$ Ar), 128.8, 128.6, 128.5, 128.5, 128.4, 128.2, 128.2, 128.0, 127.9, 127.8 (20CH Ar), 85.7 (CH_2Ph), 81.9 (C-3), 80.7 (C-1), 76.8 (C-2/C-4) 76.5 (C-2/C-4), 76.1, 75.8, 73.6 (3CH_2Ph), 64.7 (C-7), 56.1 (C-6), 45.4 (C-5), 28.0 ($(CH_3)_3$). HRMS: calcd. for $[C_{40}H_{45}NO_9SNa]^+$ 738.27072; found 738.27000, calcd. for $[C_{40}H_{49}N_2O_9S]^+$ 733.31533; found 733.31472.

(3a*S*,4*R*,5*R*,6*S*,7*R*,7a*S*)-5,6,7-Tris(benzyloxy)-4-((benzyloxy)methyl)hexahydro-3*H* benzo[*d*][1,2,3]oxathiazole 2,2-dioxide (26).

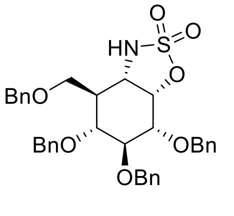

Cyclosulfamidate **25** (20 mg, 30 μmol) was dissolved in anhydrous DCM (1.2 mL) and TFA (0.11 mL) was added to the reaction mixture. The reaction was stirred overnight at rt. Then, the reaction mixture was diluted with H_2O and the aqueous phase was extracted with DCM (3x). The combined organic layers were dried over $MgSO_4$, filtered and concentrated *in vacuo*. The crude product was purified by silica gel column chromatography (Pent/EtOAc 95:5 → 70:30) to obtain **26** (13 mg, 21 μmol, 65%) as a colorless oil. ^{1}H NMR (400 MHz, $CDCl_3$) δ 7.40 – 7.22 (m, 18H, CH Ar), 7.21 – 7.14 (m, 2H, CH Ar), 4.85 (dd, *J* = 5.6, 3.1 Hz, 1H, CH-1), 4.77 (d, *J* = 11.0 Hz, 1H, CH*H*Ph), 4.73 (d, *J* = 11.7 Hz, 1H, CH*H*Ph), 4.71 – 4.60 (m, 3H, CH*H*Ph), 4.59 (d, *J* = 11.1 Hz, 1H, CH*H*Ph), 4.48 (dd, *J* = 20.9, 9.7 Hz, 3H, CH*H*Ph), 4.07 (ddd, *J* = 10.0, 8.2, 5.5 Hz, 1H, CH-6), 3.88 (dd, *J* = 7.6, 5.5 Hz, 1H, CH-3), 3.77 (dd, *J* = 5.6, 3.0 Hz, 1H, CH-2), 3.73 (dd, *J* = 9.3, 3.9 Hz, 1H, CH-7a), 3.61 (dd, *J* = 9.2, 2.5 Hz, 1H, CH-7b), 3.52 (dd, *J* = 11.8, 7.6 Hz, 1H, CH-4), 2.33 (tt, *J* = 12.2, 3.2 Hz, 1H, CH-5), 1.56 (s, 1H, NH). ^{13}C NMR (101 MHz, $CDCl_3$) δ 138.2, 137.8, 137.7, 137.1, (4C_q Ar) 128.8, 128.7, 128.7, 128.6, 128.5, 128.5, 128.1, 128.1, 128.1, 128.0, 128.0, 127.9 (20CH Ar), 82.4 (C-1), 82.1 (C-3), 77.2 (C-2), 76.9 (C-4), 74.9, 74.1, 73.7, 73.5 (4CH_2Ph), 66.6 (C-7), 55.8 (C-6), 42.8 (C-5). HRMS: calcd. for $[C_{35}H_{37}NO_7SNa]^+$ 638.21829; found 638.21792, calcd. for $[C_{35}H_{41}N_2O_7S]^+$ 633.26290; found 633.26263.

(3a*S*,4*R*,5*R*,6*S*,7*R*,7a*S*)-5,6,7-tris(benzyloxy)-4-((benzyloxy)methyl)-3-methylhexahydro-*3H*-benzo[*d*][1,2,3]oxathiazole 2,2-dioxide (27).

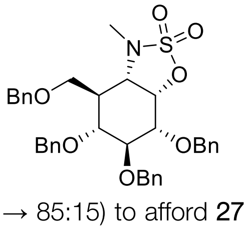

K_2CO_3 (5.64 mg, 41 μmol 1.2 eq), TBAI (1.26 mg 0.1 eq) and iodomethane (4.25 μL, 68 μmol, 2 eq) were added to a solution of **26** (21 mg, 34 μmol) in anhydrous DMF (1 mL) and the reaction was stirred at rt for 5 h. The reaction mixture was diluted with EtOAc and H_2O and the aqueous phase was extracted with EtOAc (3x). The combined organic layers were washed with water and brine, dried over $MgSO_4$, filtered and concentrated *in vacuo*. The crude product was purified by silica gel column chromatography (Pent/EtOAc 100:1 → 85:15) to afford **27** (17 mg, 27 μmol, 79%) as a colorless oil. ^{1}H NMR (500 MHz, $CDCl_3$) δ 7.38 – 7.24 (m, 18H, CH_{Ar}), 7.24 – 7.21 (m, 2H, CH_{Ar}), 4.92 – 4.85 (m, 3H, 2x CH*H*Ph, C-1), 4.81 (d, *J* = 10.8 Hz, 1H, CH*H*Ph), 4.76 (s, 2H, 2x CH*H*Ph), 4.52 (d, *J* = 11.0 Hz, 1H, CH*H*Ph), 4.44 (d, *J* = 11.7 Hz, 1H, CH*H*Ph), 4.31 (d, *J* = 11.8 Hz, 1H, CH*H*Ph), 3.91 (t, *J* = 9.3 Hz, 1H, C-3), 3.83 (dd, *J* = 9.4, 2.2 Hz, 1H, C-7), 3.59 (dd, *J* = 9.5, 3.7 Hz, 1H, C-2), 3.58 – 3.51 (m, 2H, C-6, C-7), 3.48 (dd, *J* = 11.4, 9.1 Hz, 1H, C-4), 2.70 (s, 3H, CH_3), 2.28 (tt, *J* = 11.1, 2.2 Hz, 1H, C-5). ^{13}C NMR (126 MHz, $CDCl_3$) δ 138.5, 138.4, 137.8, 137.5 (4C_q), 128.7, 128.7, 128.56, 128.3, 128.2, 128.2, 128.15, 128.1, 127.89, 127.85, 127.8 (11 CH_{Ar}), 82.1 (C-3), 76.9 (C-2), 76.6 (C-4), 75.9, 75.4, 73.4, 73.3 (4CH_2Ph), 64.5 (C-7), 62.1 (C-6), 44.7 (C-5), 36.8 (CH_3).). HRMS: calcd. for $[C_{36}H_{39}NO_7SNa]^+$ 652.23394; found 652.23376.

(3a*S*,4*R*,5*R*,6*S*,7*R*,7a*S*)-5,6,7-Trihydroxy-4-(hydroxymethyl)hexahydro-3*H*-benzo[*d*][1,2,3]oxathiazole 2,2-dioxide (6).

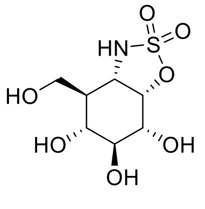

Perbenzylated **26** (13 mg, 21 μmol) was dissolved in MeOH (1 mL) and the solution was purged with N_2. Pd/C (10 wt %, 16 mg, 15 μmol, 0.7 eq) was added , the solution was purged with N_2 and the reaction was stirred overnight under H_2 atmosphere at rt. The reaction was flushed with N_2, filtered over a celite plug and concentrated *in vacuo*. The crude product was purified by silica gel column chromatography (DCM/MeOH 100:1 → 80:20) to obtain **6** (4.66 mg, 18.27 μmol, 87%) as a white solid. ^{1}H NMR (500 MHz, MeOD) δ 4.95 (t, 1H, CH-1), 3.98 (dd, *J* = 11.0, 2.6 Hz, 1H, CH-7a), 3.87 (dd, *J* = 11.1, 4.0 Hz, 1H, CH-6), 3.67 (dd, *J* = 11.0, 2.9 Hz, 1H, CH-7b), 3.61 – 3.55 (m, 2H, CH-2, CH-3), 3.39 – 3.35 (m, 1H, CH-4), 1.96 (tt, *J* = 11.2, 8.4, 2.7 Hz, 1H, CH-5). ^{13}C NMR (126 MHz, MeOD) δ 88.5 (C-1), 74.9 (C-2/C-3), 71.7 (C-2/C-3), 70.3 (C-4), 58.3 (C-7), 56.5 (C-6), 46.2 (C-5). HRMS: calcd. for $[C_7H_{14}N_1O_7S]^+$ 256,04855; found 256,04820.

(3a*S*,4*R*,5*R*,6*S*,7*R*,7a*S*)-5,6,7-trihydroxy-4-(hydroxymethyl)-3-methylhexahydro-3*H*-benzo[*d*][1,2,3]oxathiazole 2,2-dioxide (7).

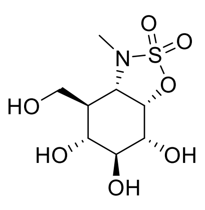

Perbenzylated **27** (17 mg, 27 μmol) was dissolved in a MeOH (1.2 mL), purged with N_2 and Pd/C (10 wt %, 17 mg, 16 μmol, 0.6 eq) was added and the reaction mixture was purged again with N_2. The reaction was stirred overnight under H_2 atmosphere at rt. The reaction mixture was flushed with N_2, filtered over a celite plug and concentrated *in vacuo*. The crude product was purified by silica gel column chromatography (DCM/MeOH 100:1 → 80:20) to obtain **7** (6.1 mg, 24 μmol, 89%) as a white solid. ^{1}H NMR (500 MHz, MeOD) δ 5.05 – 5.03 (m, 1H, CH-1), 4.04 (dd, *J* = 11.1, 2.3 Hz, 1H, CH-7B), 3.72 (dd, *J* = 10.9, 4.0 Hz, 1H, CH6), 3.67 (dd, *J* = 11.1, 2.8 Hz, 1H, CH-7A), 3.61 – 3.52 (m, 2H, CH-2, CH-3), 3.37 – 3.33 (m, 1H, CH-4), 2.94

(s, 3H, CH_3), 1.96 (tt, J = 11.1, 2.6 Hz, 1H, CH-5). ^{13}C NMR (126 MHz, MeOD) δ 83.3 (C-1), 73.6 (C2/3), 70.0 (C2/3), 68.6 (C-4), 62.3 (C-6), 56.4 (C-7), 46.2 (C-5), 36.1 (CH_3). HRMS: calcd. for $[C_8H_{16}NO_7S]^+$ 270.06420; found 270.06377.

3.2.3 Synthesis and characterization data of 8

(1*R*,2*R*,3*S*,4*S*,5*R*,6*R*)-2-azido-3,4,5-tris(benzyloxy)-6-((benzyloxy)methyl)cyclohexyl methanesulfonate (33).

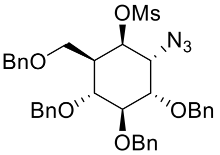

Azide **10** (50 mg, 86 µmol) was dissolved in anhydrous DCM (1 mL) and Et_3N (60 µL, 0.43 mmol, 5 eq), Me-Imidazole (69 µL, 0.86 mmol, 10 eq) and MsCl (33 µL, 0.43 mmol, 5 eq) were added to the solution at 0 ℃. The reaction was stirred for 3 h at rt. The reaction mixture was diluted with EtOAc and the organic phase was washed with 1M HCl (3x), H_2O and brine, dried over $MgSO_4$, filtered and concentrated *in vacuo*. The crude product was purified by silica gel column chromatography (Pentane/EtOAc 100:1→75:25) to obtain **31** (49 mg, 75 µmol, 87%) as a colorless oil. ^1H NMR (500 MHz, $CDCl_3$) δ 7.38 – 7.25 (m, 18H, CH Ar), 7.19 – 7.16 (m, 2H, CH Ar), 4.95 (d, J = 10.7 Hz, 1H), 4.88 – 4.84 (m, 2H, CH*H*Ph, CH-6), 4.80 (d, J = 10.7 Hz, 1H, CH*H*Ph), 4.76 (d, J = 11.5 Hz, 1H, CH*H*Ph), 4.72 (d, J = 11.5 Hz, 1H, CH*H*Ph), 4.47 – 4.42 (m, 2H, CH*H*Ph), 4.40 (d, J = 11.8 Hz, 1H, CH*H*Ph), 4.23 (t, J = 3.7 Hz, 1H, CH-1), 3.98 (dd, J = 9.6, 3.4 Hz, 1H, CH-2), 3.90 (t, J = 9.3 Hz, 1H, CH-3), 3.72 (dd, J = 9.5, 4.3 Hz, 1H, CH-7B), 3.42 (dd, J = 11.4, 8.8 Hz, 1H, CH-4), 3.34 (t, J = 9.9 Hz, 1H, CH-7A), 2.83 (s, 3H, CH_3), 2.39 (dddd, J = 11.3, 10.3, 4.3, 2.6 Hz, 1H, CH-5). ^{13}C NMR (126 MHz, $CDCl_3$) δ 138.6, 138.01, 137.6, 137.6 ($4C_q$ Ar), 128.7, 128.6, 128.6, 128.5, 128.3, 128.2, 128.1, 128.0, 128.0, 128.0, 128.0, 127.8 (20CH Ar), 83.2 (C-3), 79.2 (C-2), 77.7 (C-4), 77.1 (C-6), 76.0, 75.5, 73.6, 73.3 ($4CH_2Ph$), 66.3 (C-7), 60.9 (C-1), 40.4 (C-5), 37.6 (CH_3). HRMS: calcd. for $[C_{36}H_{39}N_3O_7SNa]^+$ 680.24009; found 680.23969, calcd. for $[C_{36}H_{43}N_4O_7S]^+$ 675.28470; found 675.28427.

(1*R*,2*S*,3*R*,4*R*,5*S*,6*R*)-2-azido-4,5,6-tris(benzyloxy)-3-((benzyloxy)methyl)cyclohexyl methanesulfonate (28).

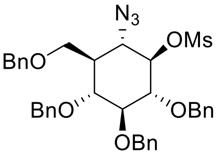

Azide **19** (0.11 g, 0.19 mmol) was dissolved in anhydrous DCM (1.73 mL) and Et_3N (0.13 mL, 0.95 mmol, 5 eq), methyl imidazole (0.15 mL, 1.9 mmol, 10 eq) and MsCl (74 µL, 0.95 mmol, 5 eq) were added to the solution at 0 ℃. The reaction was stirred for 3 h at rt. The reaction mixture was diluted with EtOAc and the organic phase was washed with 1M HCl (3x), H_2O and brine, dried over $MgSO_4$, filtered and concentrated *in vacuo*. The crude product was purified by silica gel column chromatography (Pentane/EtOAc 100:1→75:25) to obtain **28** (0.11 g, 0.17 mmol, 88%) as a white solid. ^1H NMR (500 MHz, $CDCl_3$) δ 7.37 – 7.22 (m, 18H, CH Ar), 7.18 – 7.15 (m, 2H, CH Ar), 4.90 – 4.82 (m, 5H, CH*H*Ph), 4.52 – 4.46 (m, 3H, CH*H*Ph, CH-1), 4.40 (d, J = 11.5 Hz, 1H, CH*H*Ph), 3.84 (dd, J = 9.4, 1.8 Hz, 1H, CH-7B), 3.75 – 3.70 (m, 1H, CH-6), 3.70 – 3.65 (m, 2H, CH-3, CH-7A), 3.59 – 3.53 (m, 2H, CH-2, CH-4), 2.99 (s, 3H, CH_3), 1.59 (tt, J = 11.3, 2.2 Hz, 1H, CH-5). ^{13}C NMR (126 MHz, $CDCl_3$) δ 138.1, 138.0, 137.7, 137.6 ($4C_q$ Ar), 128.6, 128.6, 128.6, 128.5, 128.1, 128.1, 128.0, 128.0, 127.9, 127.8, 127.7 (CH Ar), 85.5 (C-2/C-4), 83.8 (C-1), 80.5 (C-2/C-4), 77.1 (C-3), 75.9, 75.8, 75.8, 73.4 ($4CH_2Ph$), 64.6 (C-7), 60.5 (C-6), 44.6 (C-5), 39.2 (CH_3). HRMS: calcd. for $[C_{36}H_{39}N_3O_7SNa]^+$ 680.24009; found 680.23974, calcd. for $[C_{36}H_{43}N_4O_7S]^+$ 675.28470; found 675.28437.

((((1*R*,2*S*,3*S*,4*S*,5*S*,6*R*)-4,5-diazido-6-((benzyloxy)methyl)cyclohexane-1,2,3-triyl)tris(oxy))tris(methylene))tribenzene (29).

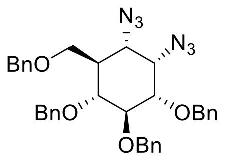

Mesylate **28** (0.1 g, 0.15 mmol) was dissolved in anhydrous DMF (4 mL) and NaN_3 (0.1 g, 1.5 mmol, 10 eq) was added and the reaction was stirred for 18 h at 100 ℃. The reaction mixture was cooled to rt and EtOAc and H_2O were added to the reaction mixture. The aqueous layers was extracted with EtOAc (2x). The combined organic layers were washed with H_2O and brine, dried over $MgSO_4$, filtered and concentrated *in vacuo*. The crude product was purified by silica gel column chromatography (Pentane/EtOAc 100:1→70:30) to obtain **29**[18] (67 mg, 0.11 mmol, 71%) as a white solid. ^1H NMR (500 MHz, $CDCl_3$) δ 7.38 – 7.24 (m, 18H, CH Ar), 7.20 – 7.16 (m, 2H, CH Ar), 4.93 (d, J = 10.6 Hz, 1H, CH*H*Ph)), 4.88 (d, J = 10.8 Hz, 1H, CH*H*Ph), 4.80 (d, J = 10.7 Hz, 1H, CH*H*Ph), 4.75 (d, J = 11.7 Hz, 1H, CH*H*Ph), 4.71 (d, J = 11.7 Hz, 1H, CH*H*Ph), 4.48 (d, J = 7.4 Hz, 1H, CH*H*Ph), 4.45 (d, J = 6.6 Hz, 1H, CH*H*Ph), 4.34 (d, J = 11.5 Hz, 1H, CH*H*Ph), 4.06 (t, J = 3.1 Hz, 1H, CH-1), 3.87 (t, J = 9.4 Hz, 1H, CH-3), 3.83 (dd, J = 9.5, 1.8 Hz, 1H, CH-7B), 3.56 (ddd, J = 9.2, 6.6, 4.0 Hz, 2H, CH-4, CH-7A), 3.53 – 3.49 (m, 2H, CH-2, CH-6), 2.03 (tt, J = 11.1, 2.2 Hz, 1H, CH-5). ^{13}C NMR (126 MHz, $CDCl_3$) δ 138.6, 138.4, 138.0, 137.6 ($4C_q$ Ar), 128.7, 128.5, 128.5, 128.2, 128.1, 128.1, 128.0, 128.0, 127.8, 127.8 (20CH Ar), 83.1 (C-3), 80.3 (C-2), 77.6 (C-4), 76.0, 75.7, 73.3, 73.2 ($4CH_2Ph$), 65.0 (C-7), 63.6 (C-1), 57.6 (C-6), 42.5 (C-5). HRMS: calcd. for $[C_{36}H_{35}N_6O_4Na]^+$ 627.26902; found 627.26879, calcd. for $[C_{36}H_{39}N_7O_4]^+$ 622.31363; found 622.31346.

(1*S*,2*S*,3*S*,4*S*,5*R*,6*R*)-3,4,5-tris(benzyloxy)-6-((benzyloxy)methyl)cyclohexane-1,2-diamine (30). Diazo-

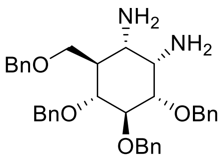

compound **29** (80 mg, 0.13 mmol) was dissolved in anhydrous THF (4.6 mL) and the solution was purged with N_2. PtO_2 (12 mg, 52 µmol, 0.4 eq) was added and again the reaction mixture was purged with N_2. The reaction mixture was stirred under H_2 atmosphere for 3 h at rt. The reaction was flushed with N_2, filtered over a celite plug and concentrated *in vacuo* to obtain **30** (66 mg, 0.12 mmol, 92%) as a colorless oil. ^{1}H NMR (500 MHz, $CDCl_3$) δ 7.37 – 7.21 (m, 20H, CH Ar), 4.93 (d, *J* = 10.7 Hz, 1H, CH*H*Ph), 4.90 (d, *J* = 10.9 Hz, 1H, CH*H*Ph), 4.80 (d, *J* = 10.7 Hz, 1H, CH*H*Ph), 4.69 (d, *J* = 11.7 Hz, 1H, CH*H*Ph), 4.65 (d, *J* = 11.7 Hz, 1H, CH*H*Ph), 4.50 (d, *J* = 10.9 Hz, 1H, CH*H*Ph), 4.44 (d, *J* = 12.0 Hz, 1H, CH*H*Ph), 4.40 (d, *J* = 12.0 Hz, 1H, CH*H*Ph), 3.92 (t, *J* = 9.3 Hz, 1H, CH-3), 3.78 (dd, *J* = 9.5, 2.6 Hz, 1H, CH-7B), 3.62 (dd, *J* = 9.4, 2.6 Hz, 1H, CH-7A), 3.53 (dd, *J* = 10.9, 9.2 Hz, 1H, CH-4), 3.46 (dd, *J* = 9.5, 3.8 Hz, 1H, CH-2), 3.43 (t, *J* = 3.5 Hz, 1H, CH-1), 2.85 (dd, *J* = 11.0, 3.1 Hz, 1H, CH-6), 1.93 (tt, *J* = 11.0, 2.6 Hz, 1H, CH-5), 1.35 (s, 4H, $2NH_2$). ^{13}C NMR (126 MHz, $CDCl_3$) δ 139.2, 139.0, 138.6, 138.6 (4C_q Ar), 128.5, 128.5, 128.4, 128.1, 128.0, 127.8, 127.8, 127.6, 127.6 (20CH Ar), 83.2 (C-4), 81.8 (C-2), 79.1 (C-3), 75.7, 75.3, 73.1, 72.1 (4CH_2Ph), 66.0 (C-7) , 53.7 (C-1), 49.4 (C-6), 43.8 (C-5). HRMS: calcd. for $[C_{35}H_{41}N_2O_4]^+$ 553.30608; found 553.30560.

(3a*S*,4*S*,5*S*,6*R*,7*R*,7a*S*)-4,5,6-tris(benzyloxy)-7-((benzyloxy)methyl)octahydrobenzo[*c*][1,2,5]thiadiazole 2,2-dioxide (31).

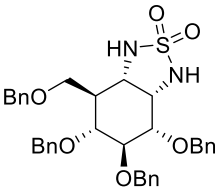

Diamine **30** (51 mg, 92 µmol) was dissolved in anhydrous pyridine (10 mL) and sulfamide (18 mg, 1.85 mmol, 20 eq) was added to the reaction solution. The reaction was refluxed for 6 h. After completion the reaction mixture was concentrated *in vacuo* and coevaporated with toluene (3x). The crude product was dissolved in DCM, H_2O was added and the aqueous layer was extracted with DCM (3x). The combined organic layers were dried over $MgSO_4$, filtered and concentrated *in vacuo*. The crude product was purified by silica gel column chromatography (Pentane/EtOAc 95:5→70:30) to obtain **31** (51 mg, 84 µmol, 91%) as a colorless oil. ^{1}H NMR (500 MHz, $CDCl_3$) δ 7.36 – 7.24 (m, 18H, CH Ar), 7.21 – 7.17 (m, 2H, CH Ar), 4.85 (d, *J* = 5.1 Hz, 1H, (NH), 4.77 (d, *J* = 11.1 Hz, 1H, CH*H*Ph), 4.74 – 4.68 (m, 3H, CH*H*Ph), 4.64 – 4.59 (m, 2H, CH*H*Ph, NH), 4.48 – 4.42 (m, 2H, CH*H*Ph), 4.40 (d, *J* = 11.9 Hz, 1H, CH*H*Ph), 4.14 (q, *J* = 5.2 Hz, 1H, CH-1), 3.90 (t, *J* = 7.3 Hz, 1H, CH-3), 3.76 (dt, *J* = 10.7, 5.4 Hz, 1H, CH-6), 3.69 – 3.65 (m, 2H, CH-2, C-7B), 3.60 (dd, *J* = 9.3, 5.0 Hz, 1H, CH-7A), 3.41 (dd, *J* = 11.3, 7.5 Hz, 1H, CH-4), 2.40 (tdd, *J* = 11.0, 5.0, 2.7 Hz, 1H, CH-5). ^{13}C NMR (126 MHz, $CDCl_3$) δ 138.2, 138.1, 137.8, 137.3 (4C_q Ar), 128.8, 128.7, 128.6, 128.6, 128.4, 128.2, 128.0, 128.0, 128.0, 127.9 (20CH Ar), 81.1 (C-3), 77.4 (C-4), 77.3 (C-2), 74.6, 74.6, 73.4, 73.3 (4CH_2Ph), 67.7 (C-7), 57.7 (C-1), 56.1 (C-6), 42.9 (C-5). HRMS: calcd. for $[C_{35}C_{39}N_2O_6S]^+$ 615.25233; found 615.25204.

(3a*S*,4*S*,5*S*,6*R*,7*R*,7a*S*)-4,5,6-trihydroxy-7-(hydroxymethyl)octahydrobenzo[*c*][1,2,5]thiadiazole 2,2-dioxide (8).
Cyclosulfamide **31** (30 mg, 49 µmol) was dissolved in a 4:1 mixture of Methanol (2 mL) and DCM (0.5 mL), purged

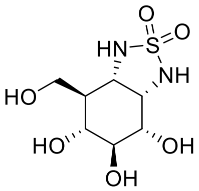

with N_2. Pd/C (10 wt%, 43 mg, 40 µmol, 0.8 eq) was added and the reaction mixture was purged again with N_2. The reaction was stirred for 18 h under H_2 atmosphere at rt. The reaction mixture was flushed with N_2, filtered over a celite plug and concentrated *in vacuo*. The crude product was purified by silica gel column chromatography (DCM/Methanol 100:1→70:30) to obtain **8** (12 mg, 47 µmol, 96%) as a white solid. ^{1}H NMR (600 MHz, MeOD) δ 4.15 (t, *J* = 4.6 Hz, 1H, CH-1), 3.94 (dd, *J* = 10.9, 3.2 Hz, 1H, CH-7B), 3.74 (dd, *J* = 10.9, 3.1 Hz, 1H, CH-7A), 3.67 (dd, *J* = 11.0, 4.8 Hz, 1H, CH-6), 3.64 (t, *J* = 9.4 Hz, 1H, CH-3), 3.54 (dd, *J* = 9.6, 4.5 Hz, 1H, CH-2), 3.34 – 3.29 (m, 2H, MeOD, CH-4), 2.00 (tt, *J* = 11.1, 3.1 Hz, 1H, CH-5). ^{13}C NMR (151 MHz, MeOD) δ 75.0 (C-3), 72.1 (C-2), 70.9 (C-4), 63.4 (C-1), 59.3 (C-7), 56.1 (C-6), 46.1 (C-5). HRMS: calcd. for $[C_7H_{15}N_2O_6S]^+$ 255.06453; found 255.06425.

4. NMR spectra

^{1}H-NMR and ^{13}C-NMR of **10** in $CDCl_3$

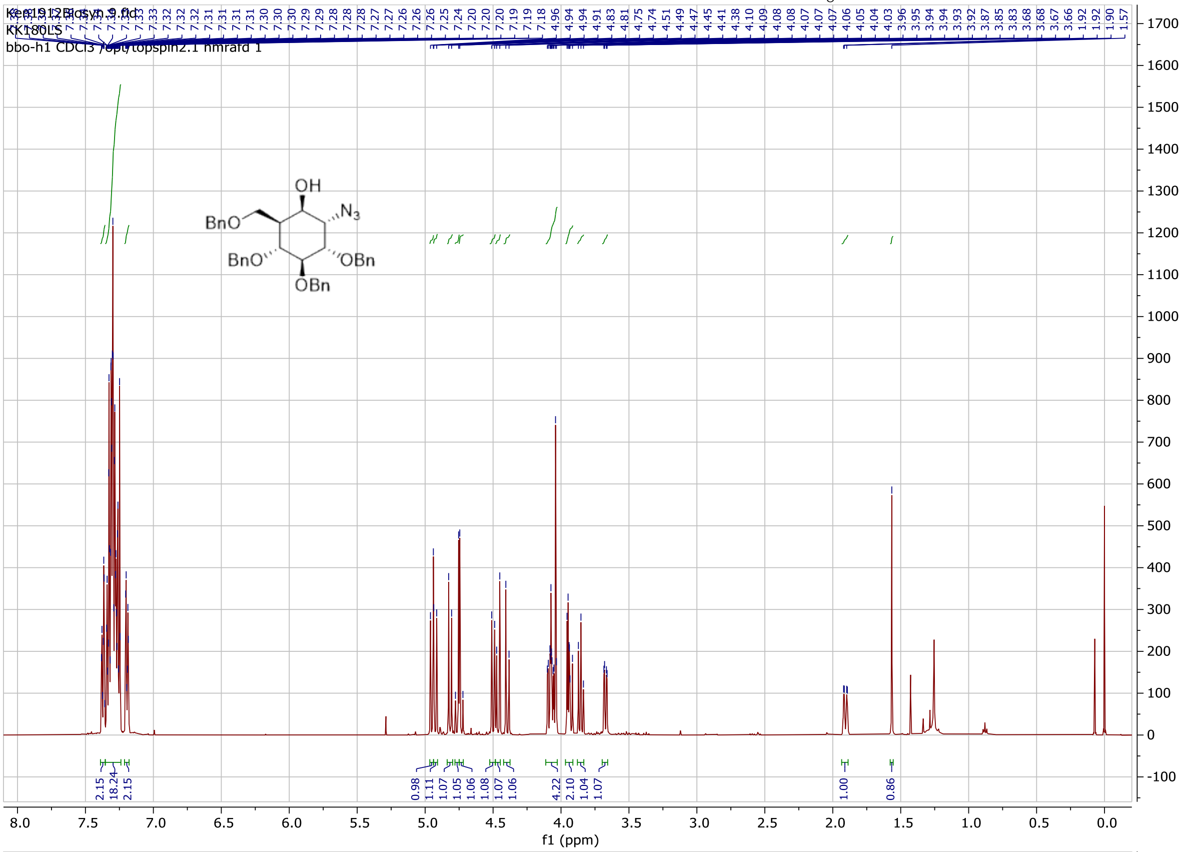

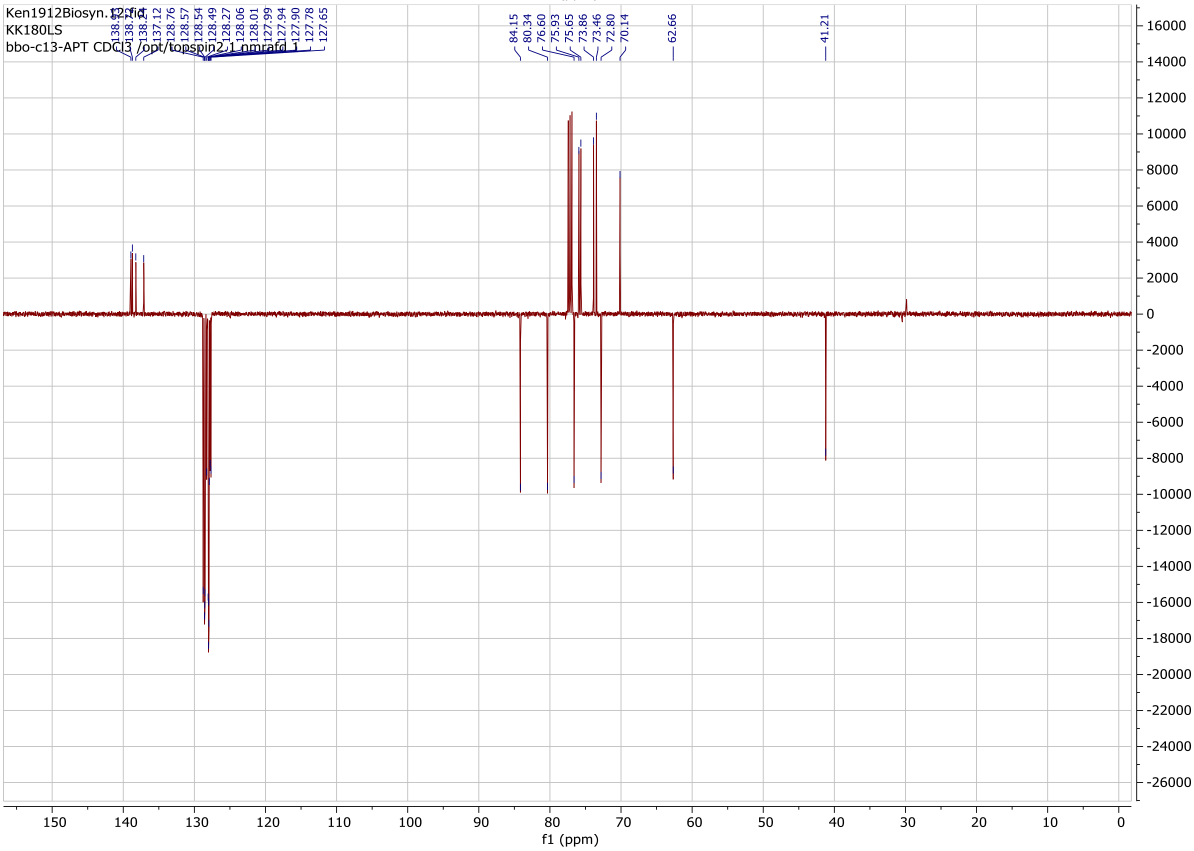

^{1}H-NMR and ^{13}C-NMR spectra of **11** in $CDCl_3$

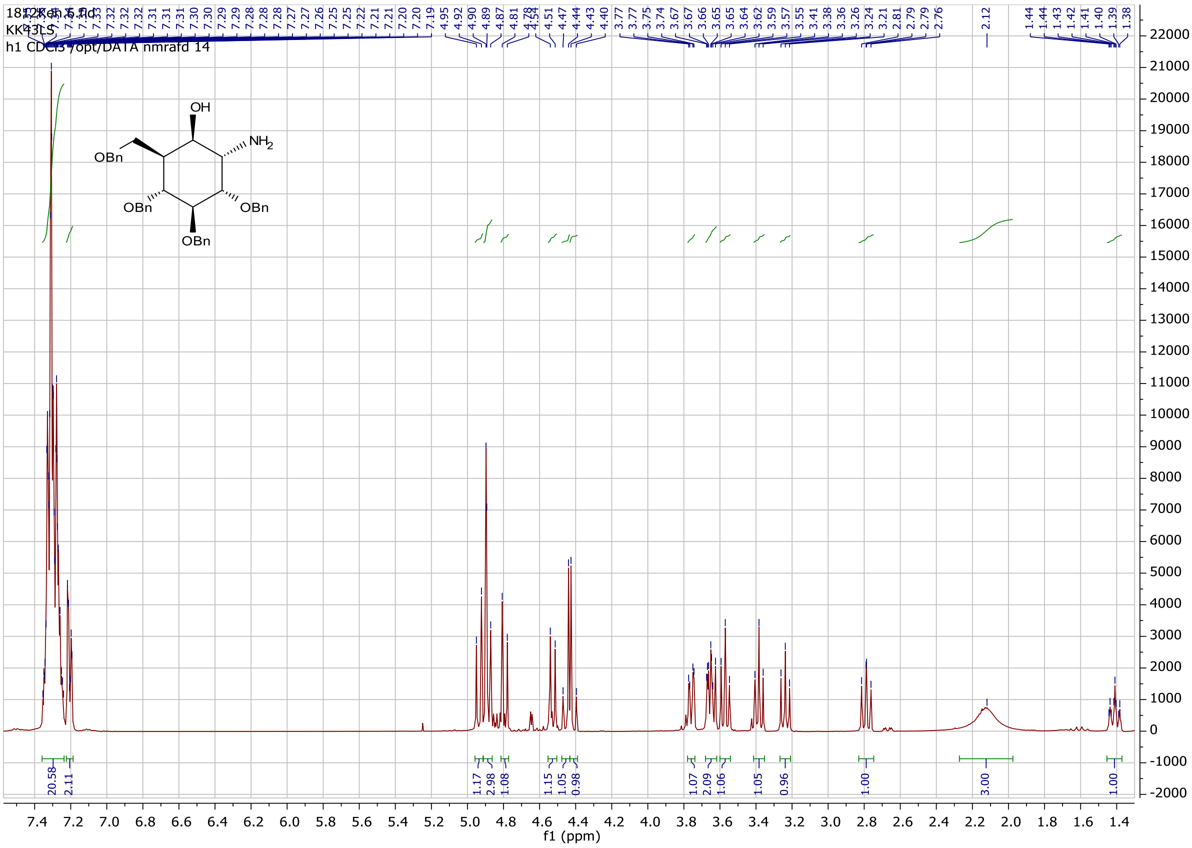

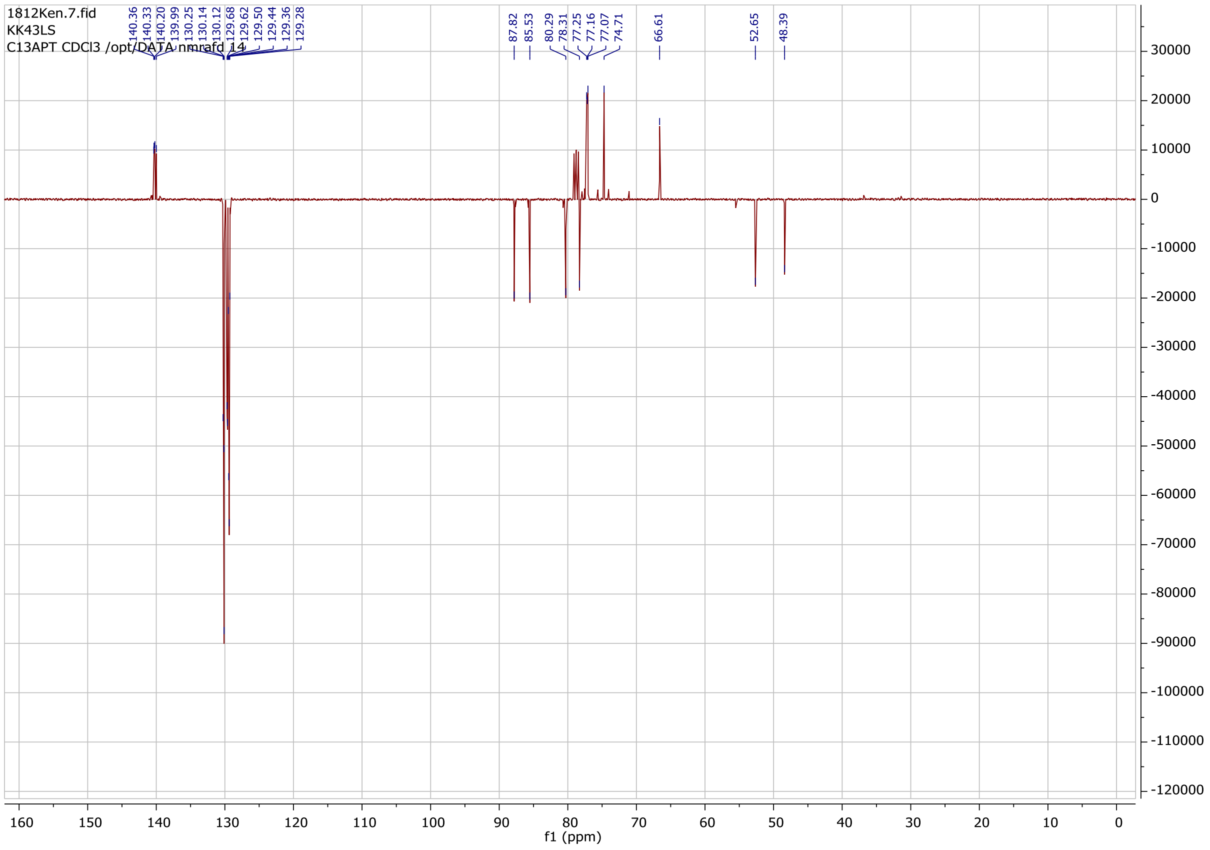

^{1}H-NMR and ^{13}C-NMR spectra of **12** in $CDCl_3$

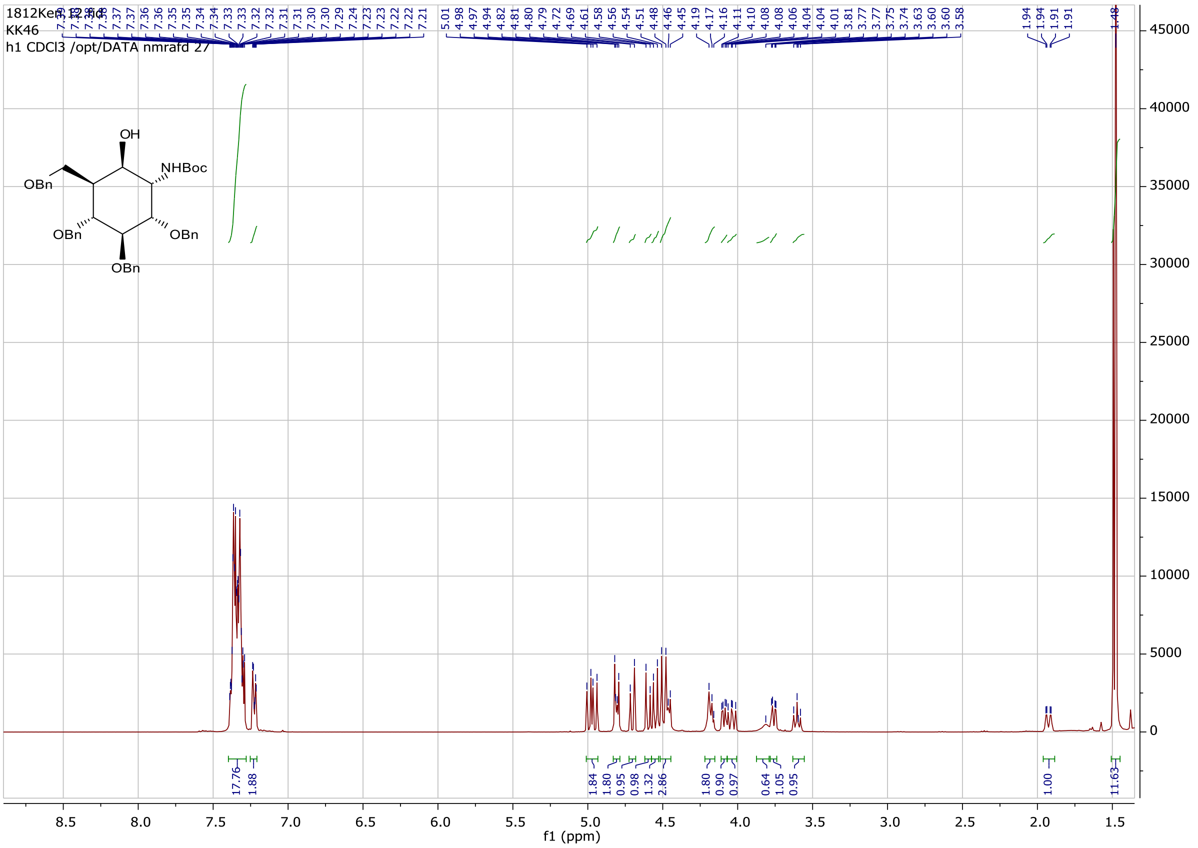

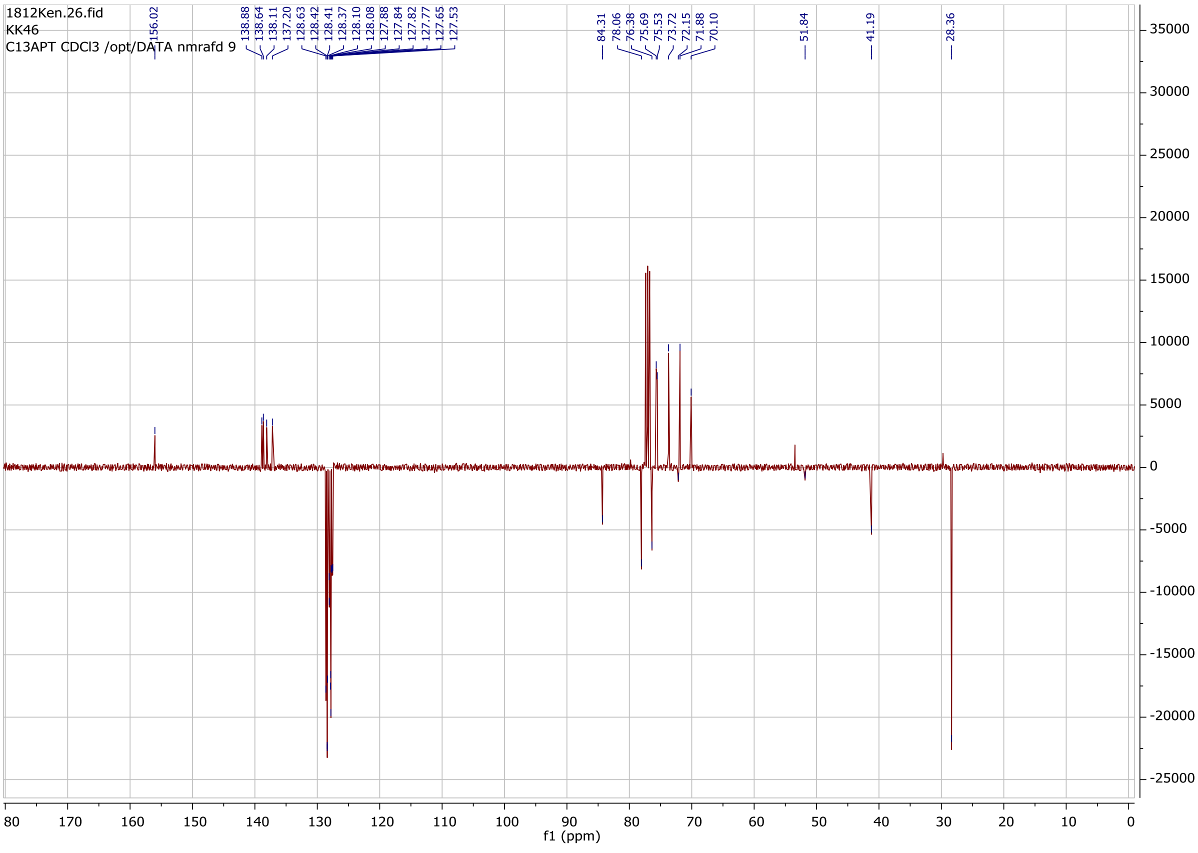

^{1}H-NMR and ^{13}C-NMR spectra of **13** in $CDCl_3$

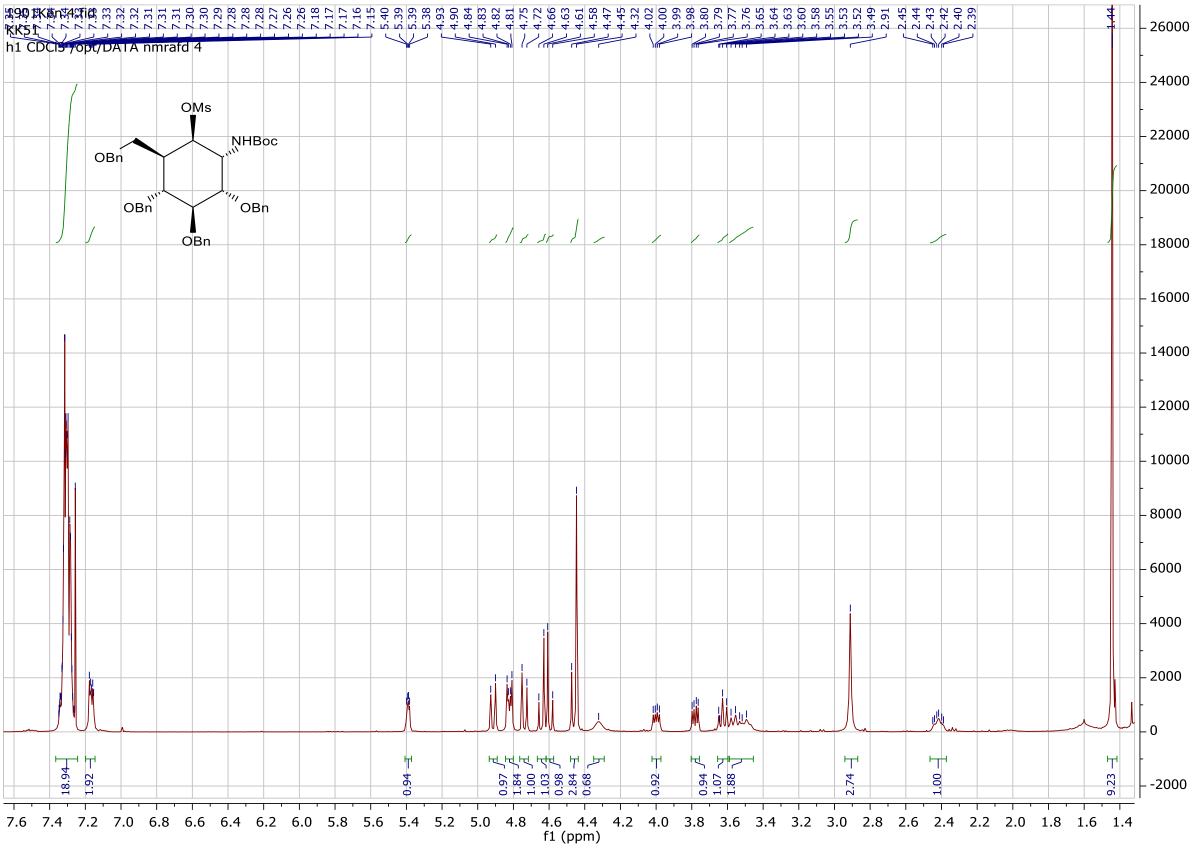

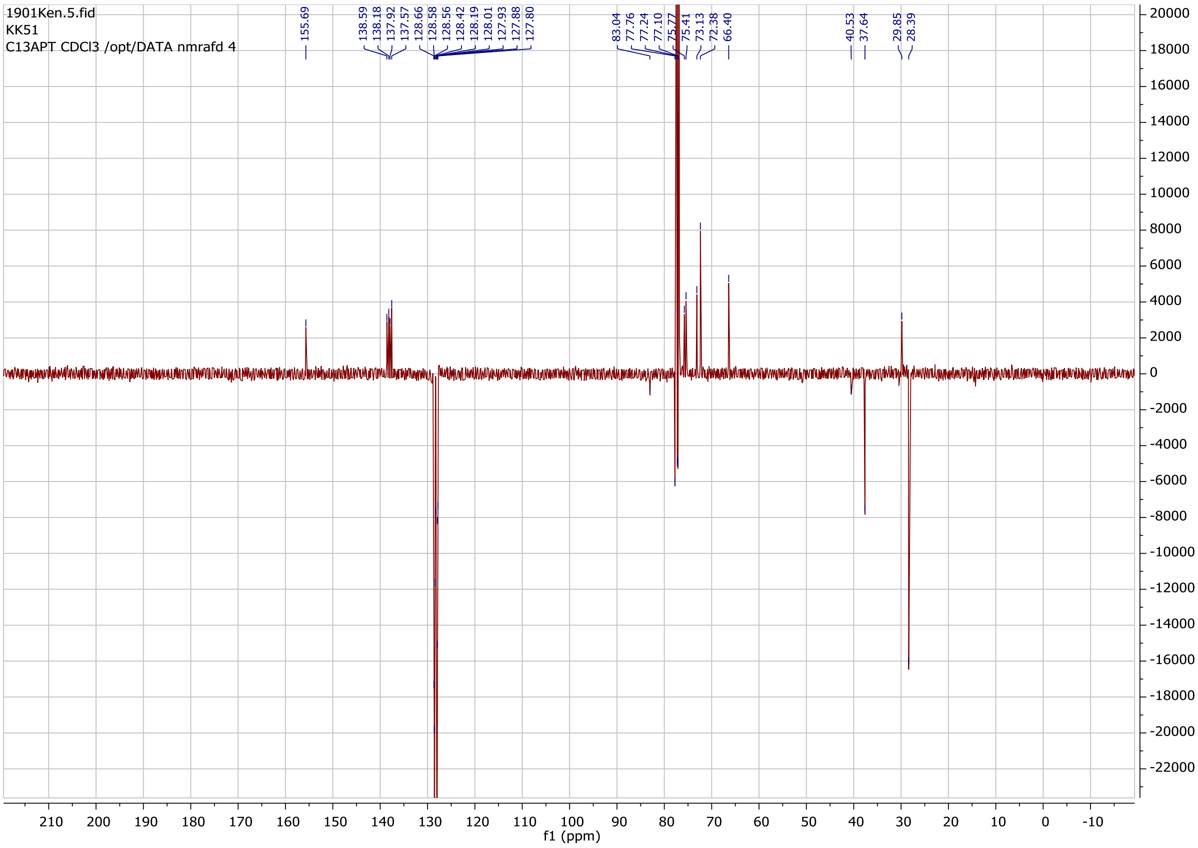

^{1}H-NMR and ^{13}C-NMR spectra of **14** in $CDCl_3$

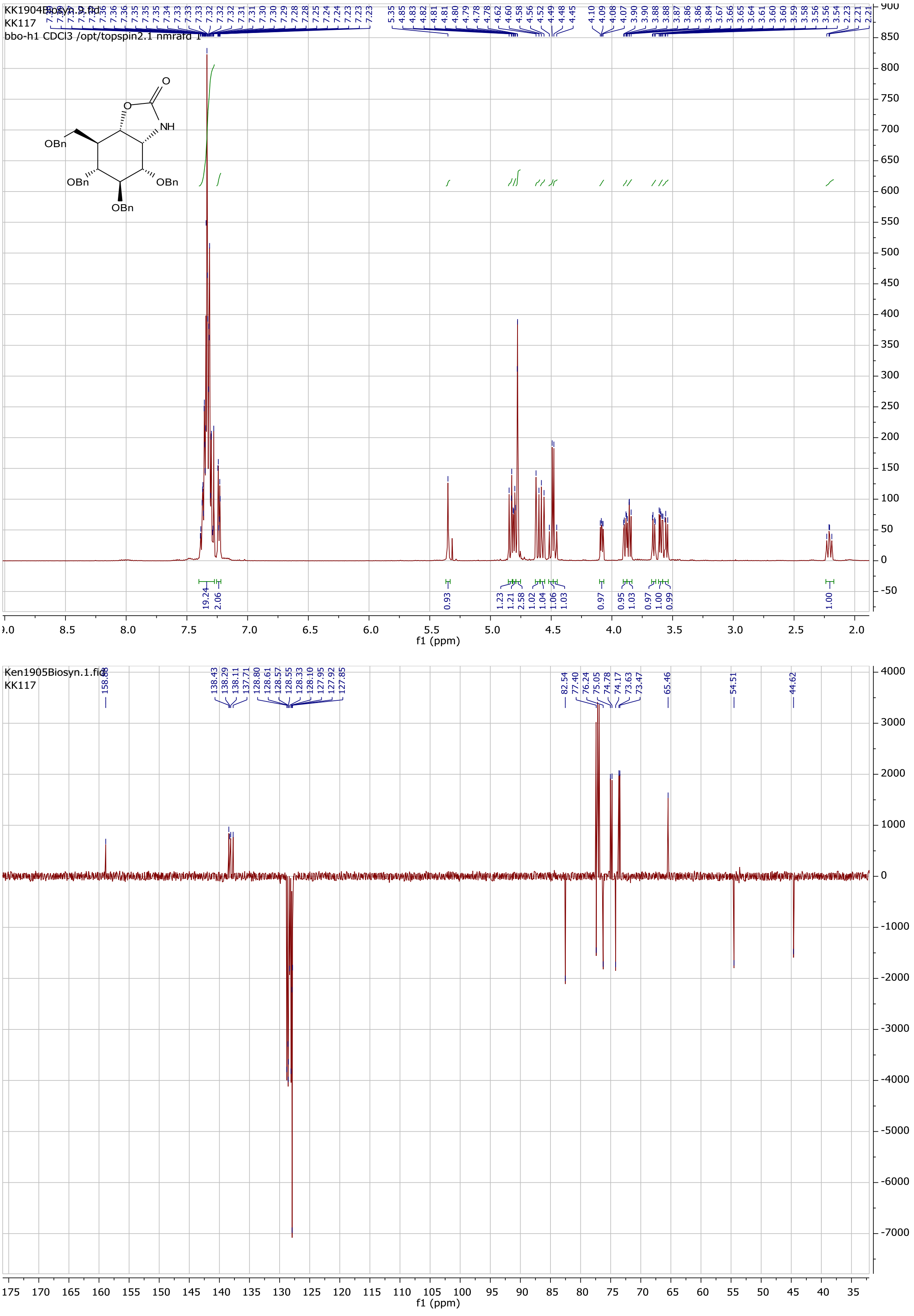

^{1}H-NMR and ^{13}C-NMR spectra of **15** in $CDCl_3$

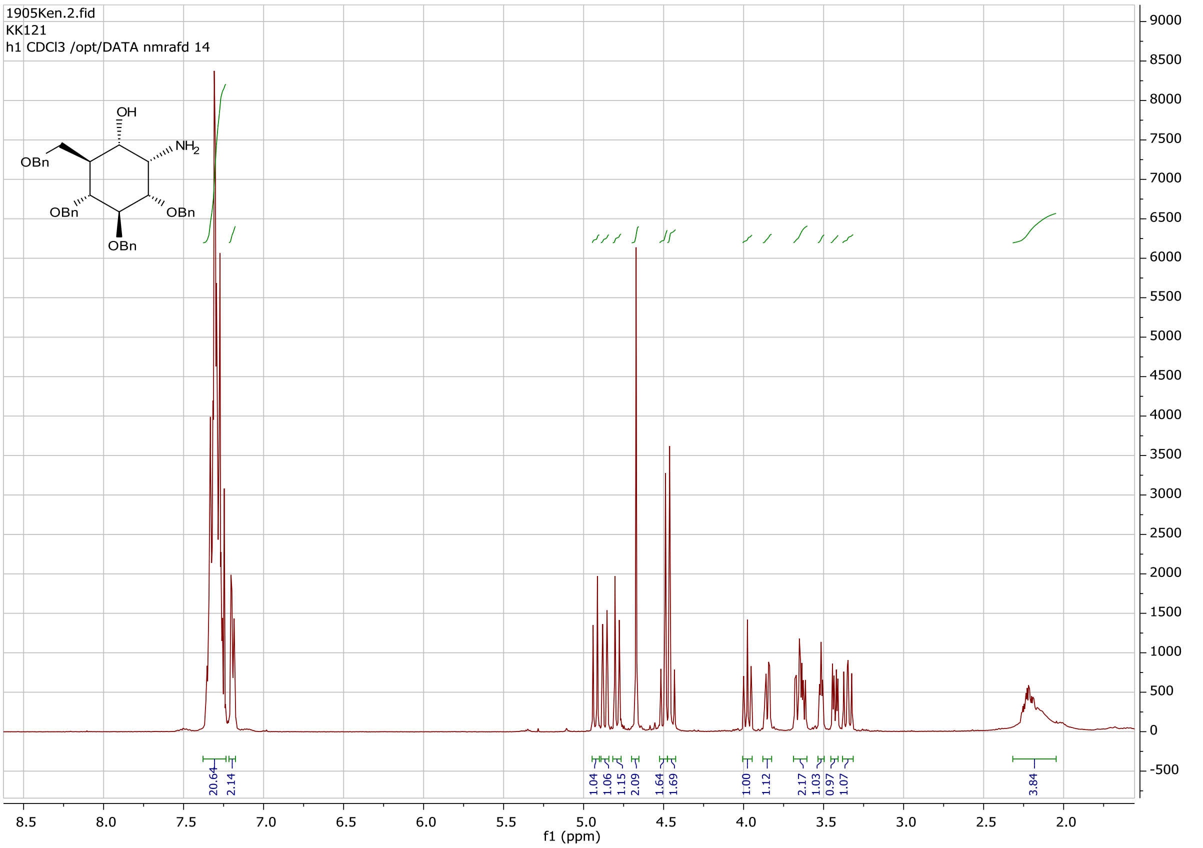

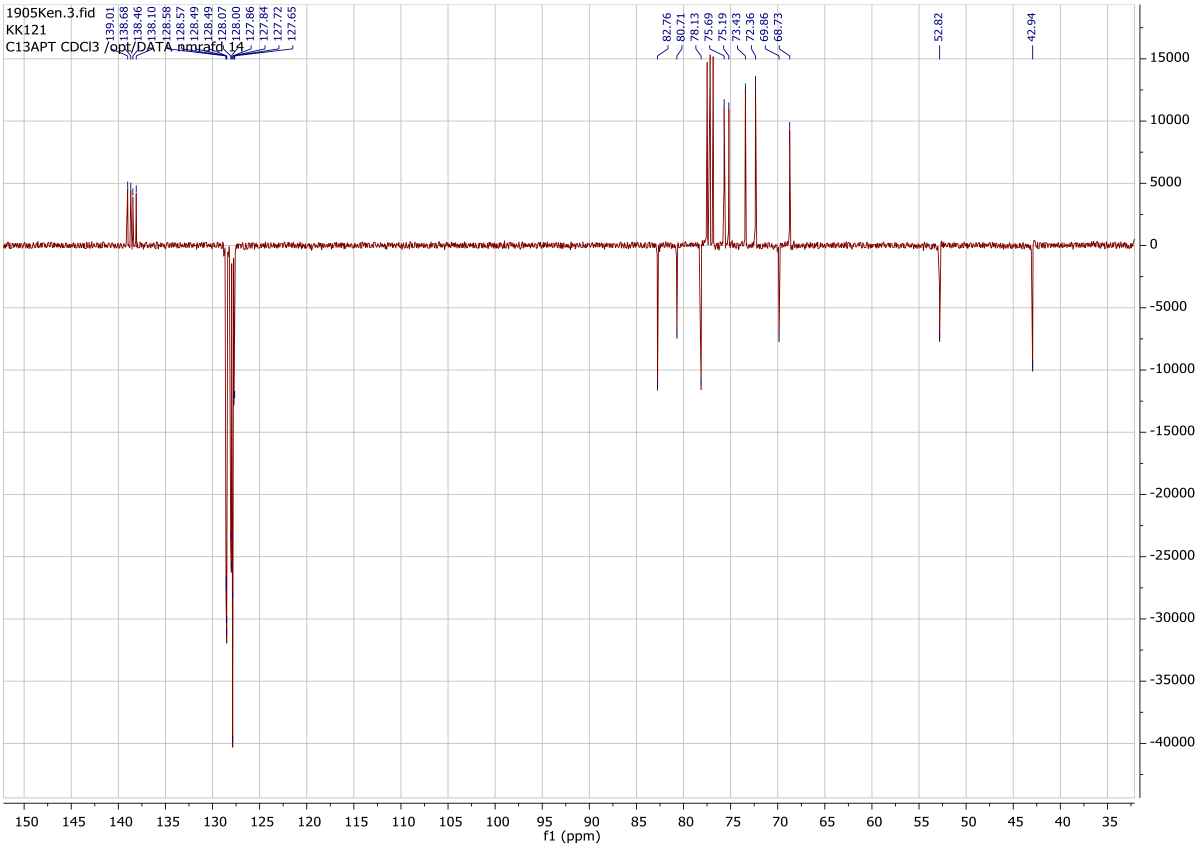

^{1}H-NMR and ^{13}C-NMR spectra of **16** in $CDCl_3$

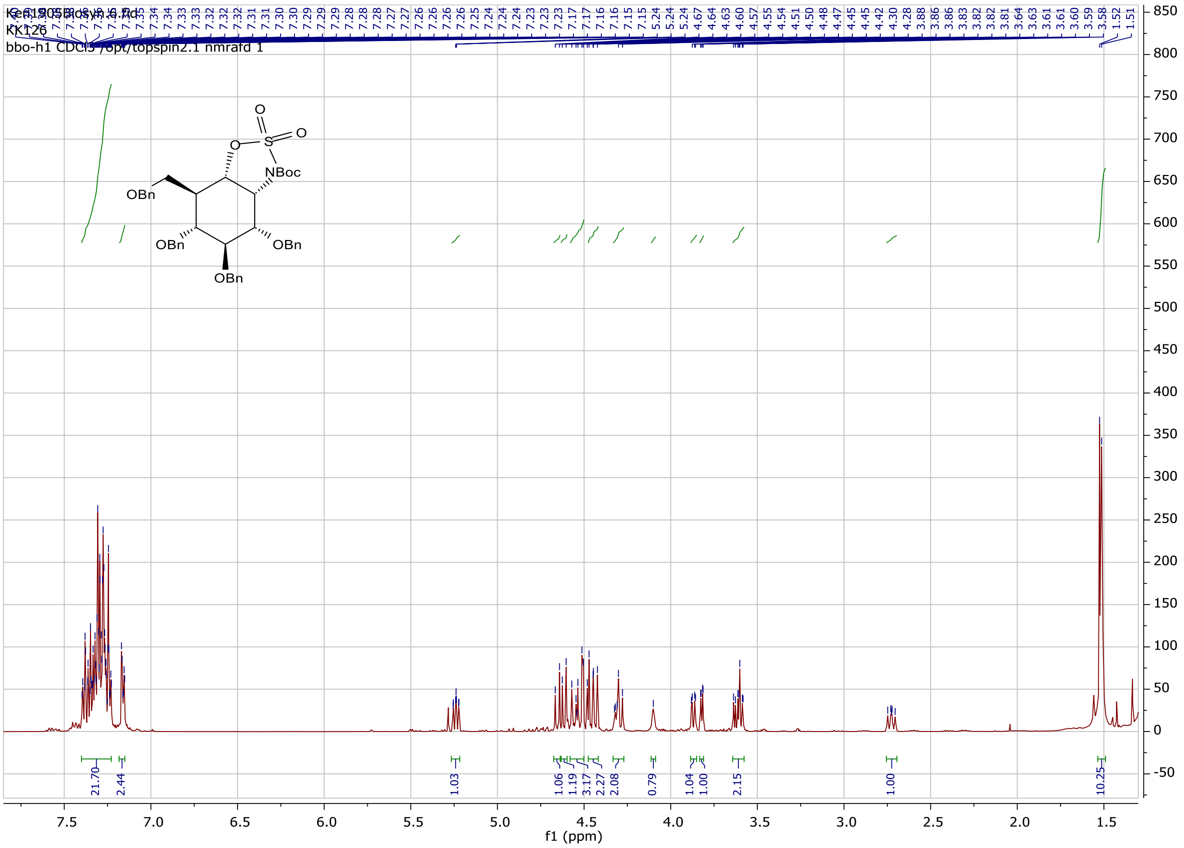

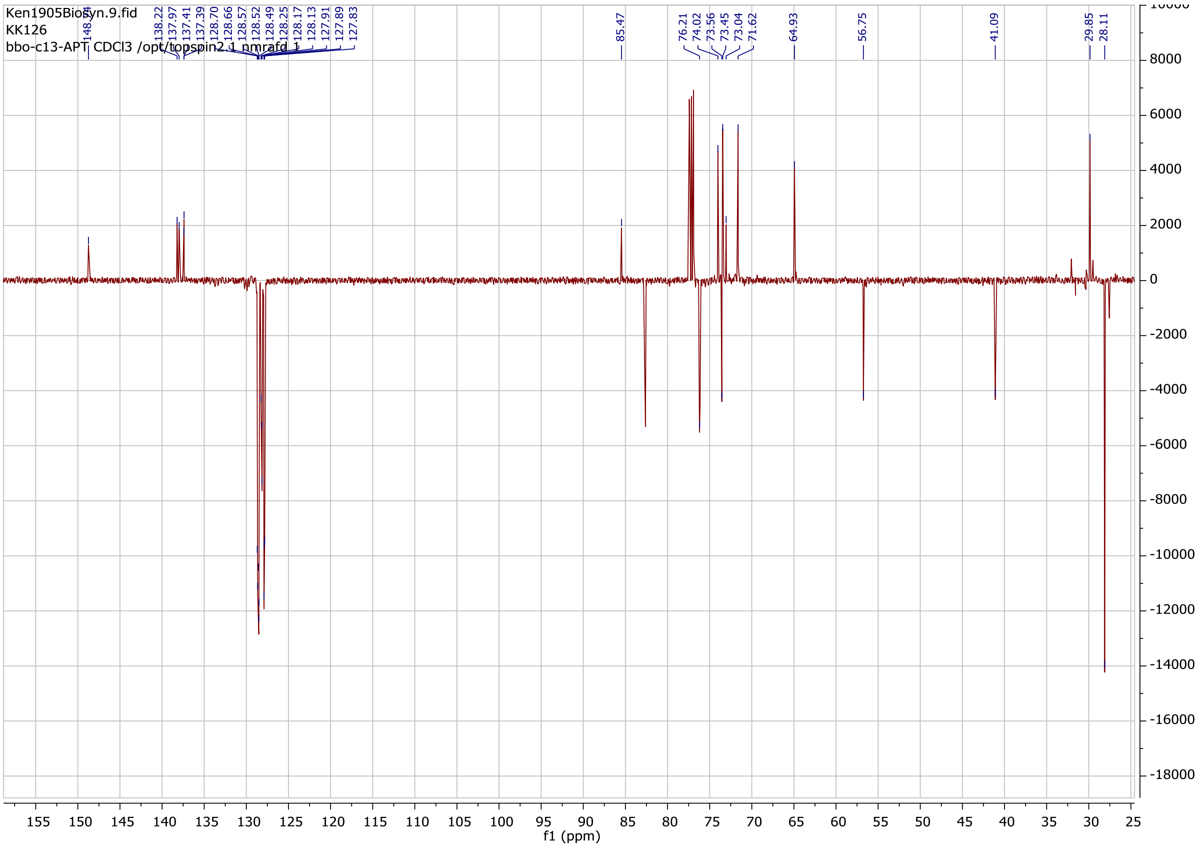

^{1}H-NMR and ^{13}C-NMR spectra of **17** in $CDCl_3$

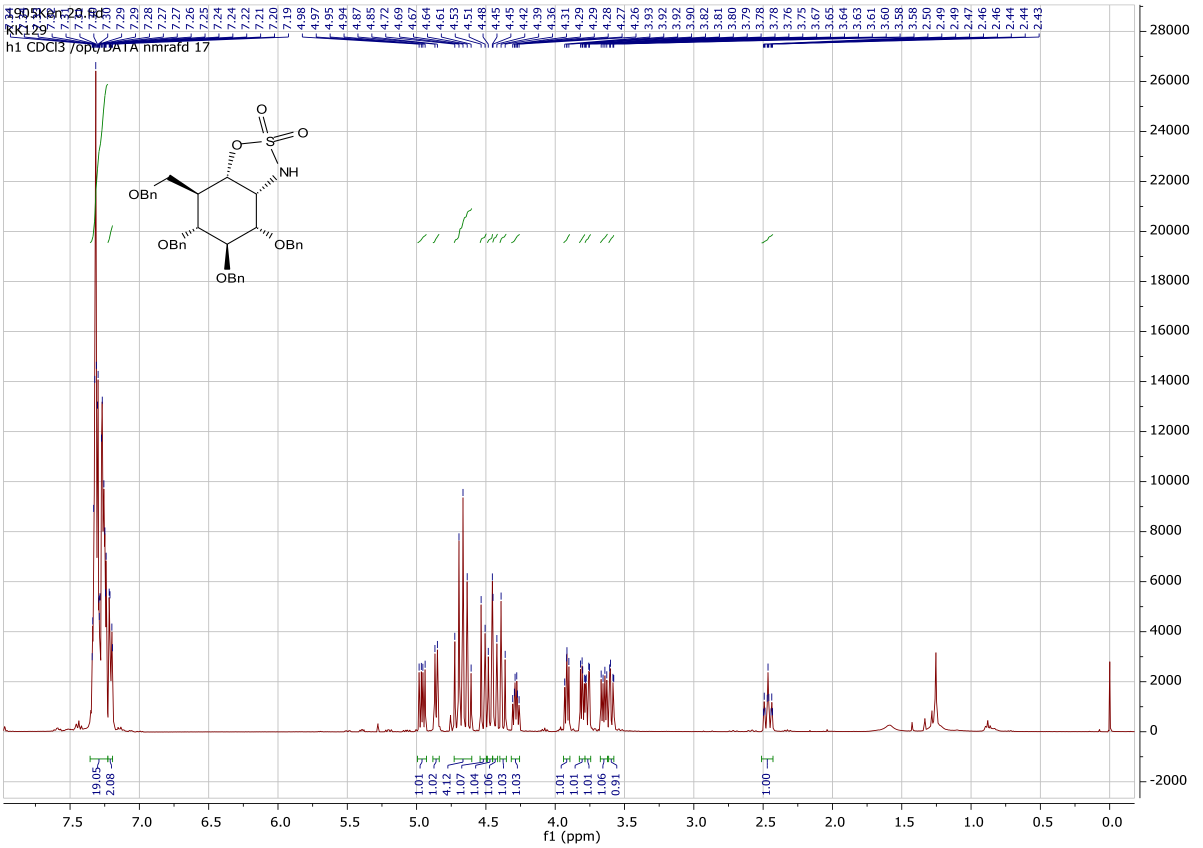

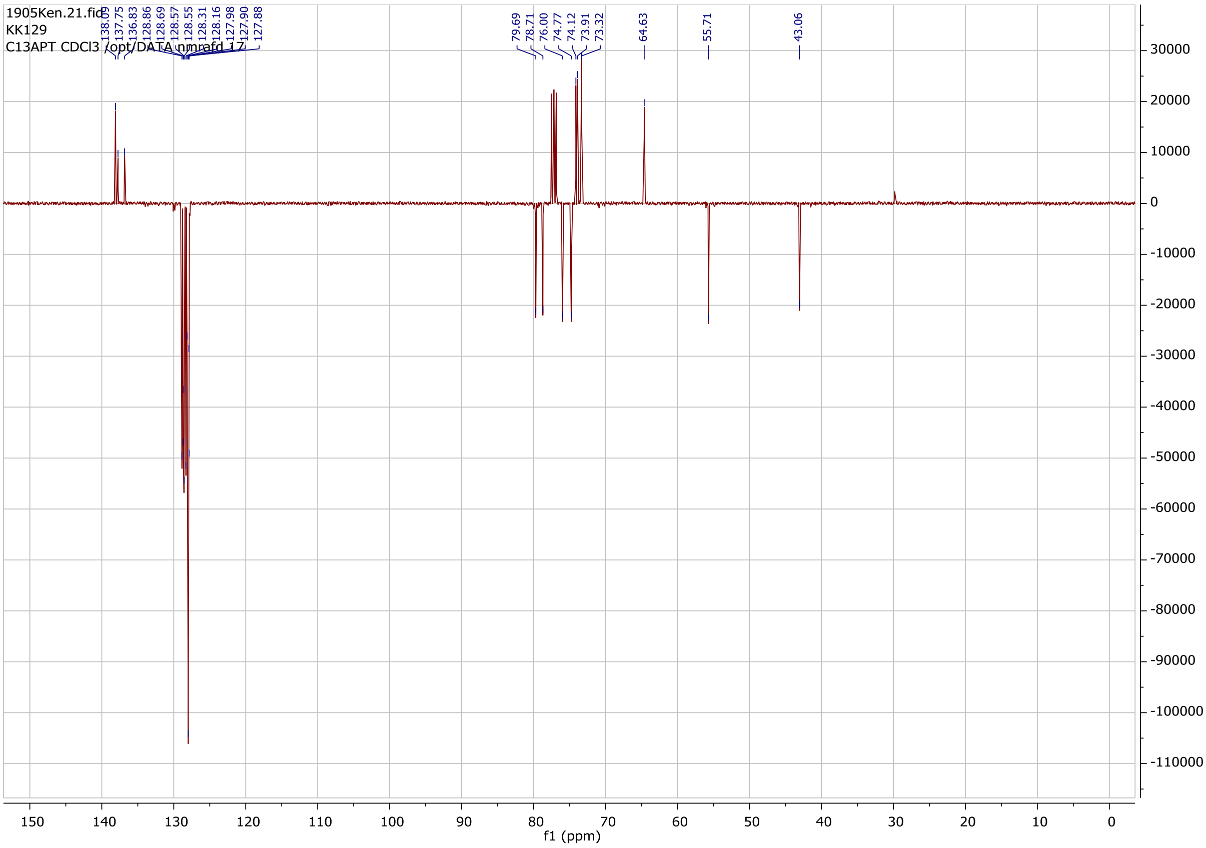

^{1}H-NMR and ^{13}C-NMR spectra of **18** in $CDCl_3$

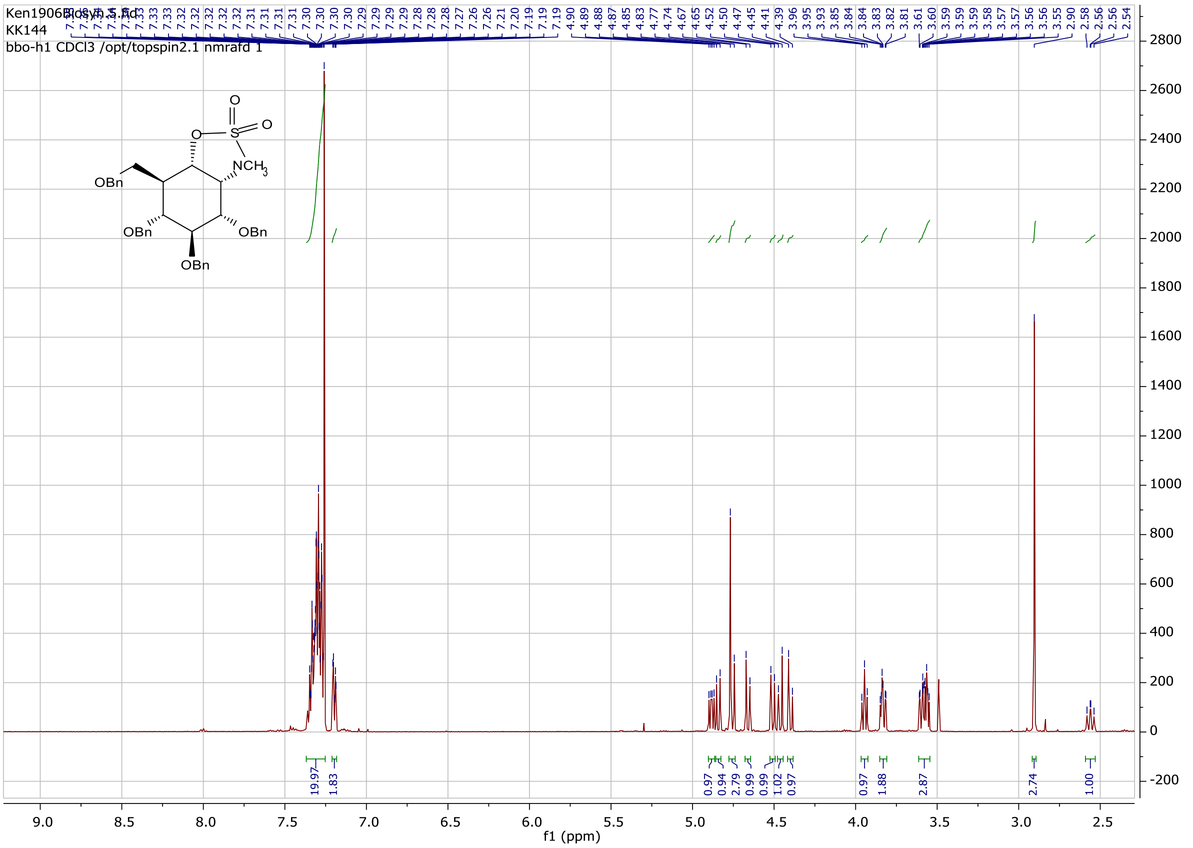

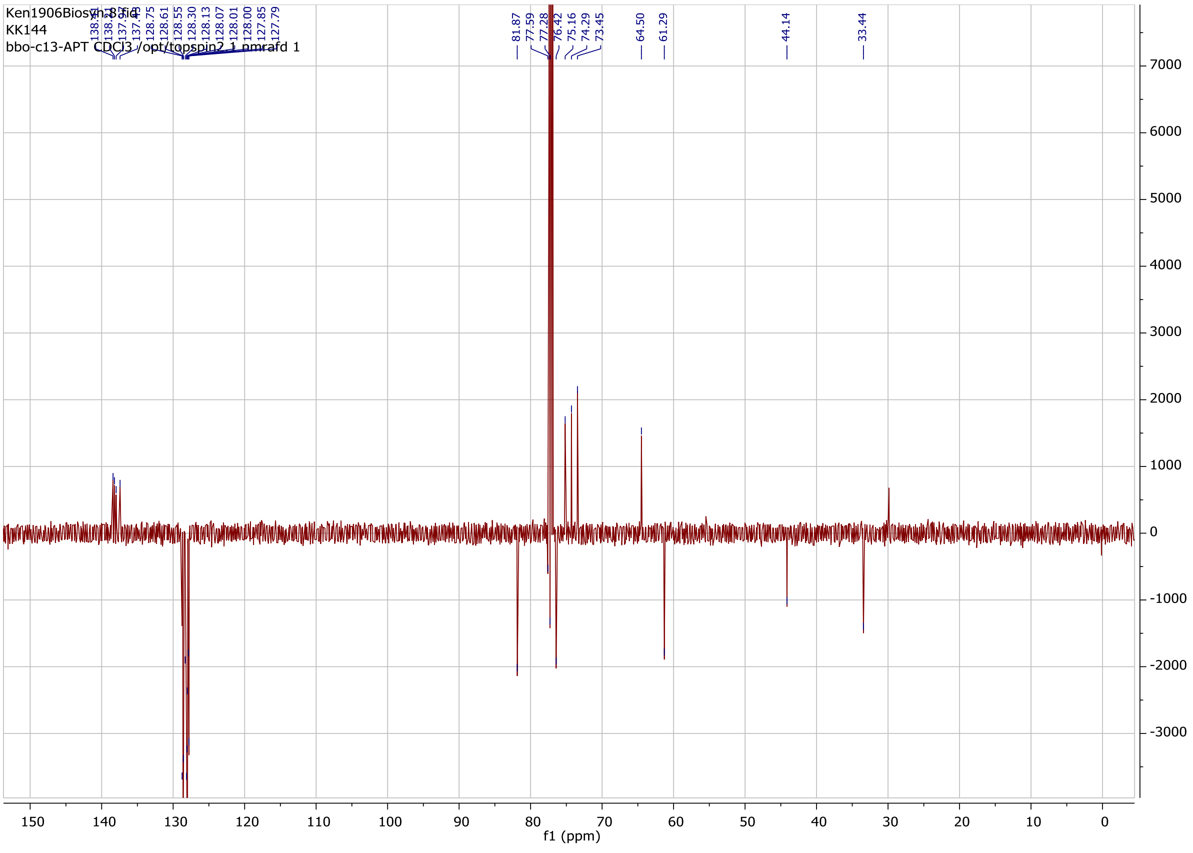

^{1}H-NMR and ^{13}C-NMR spectra of **4** in MeOD

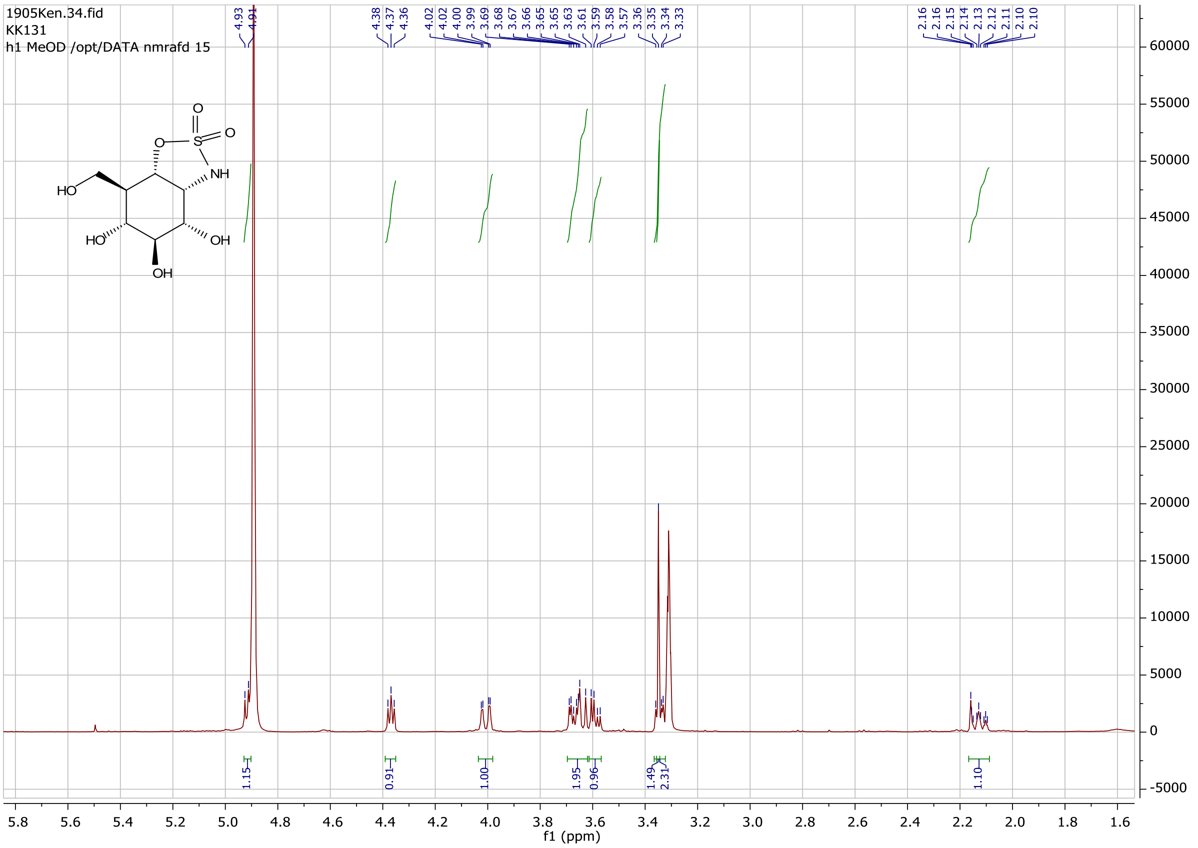

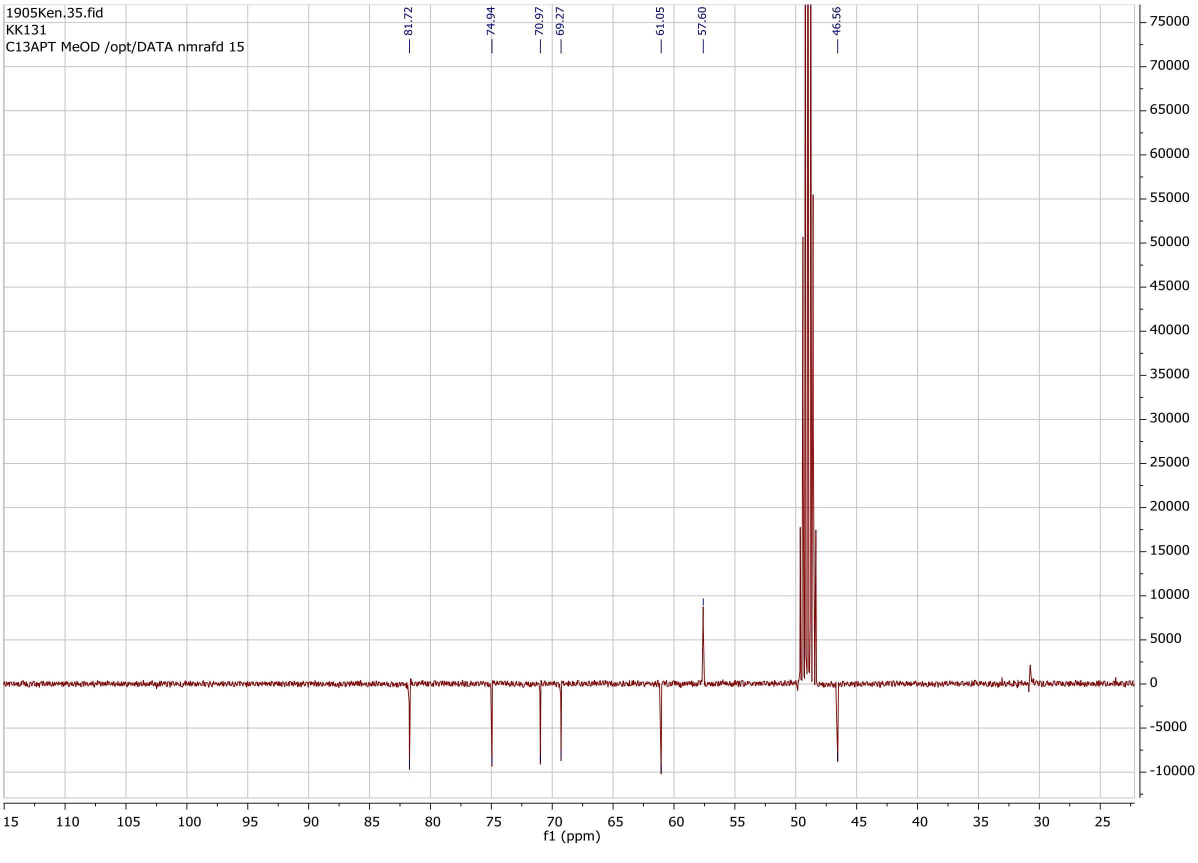

^{1}H-NMR and ^{13}C-NMR spectra of **5** in MeOD

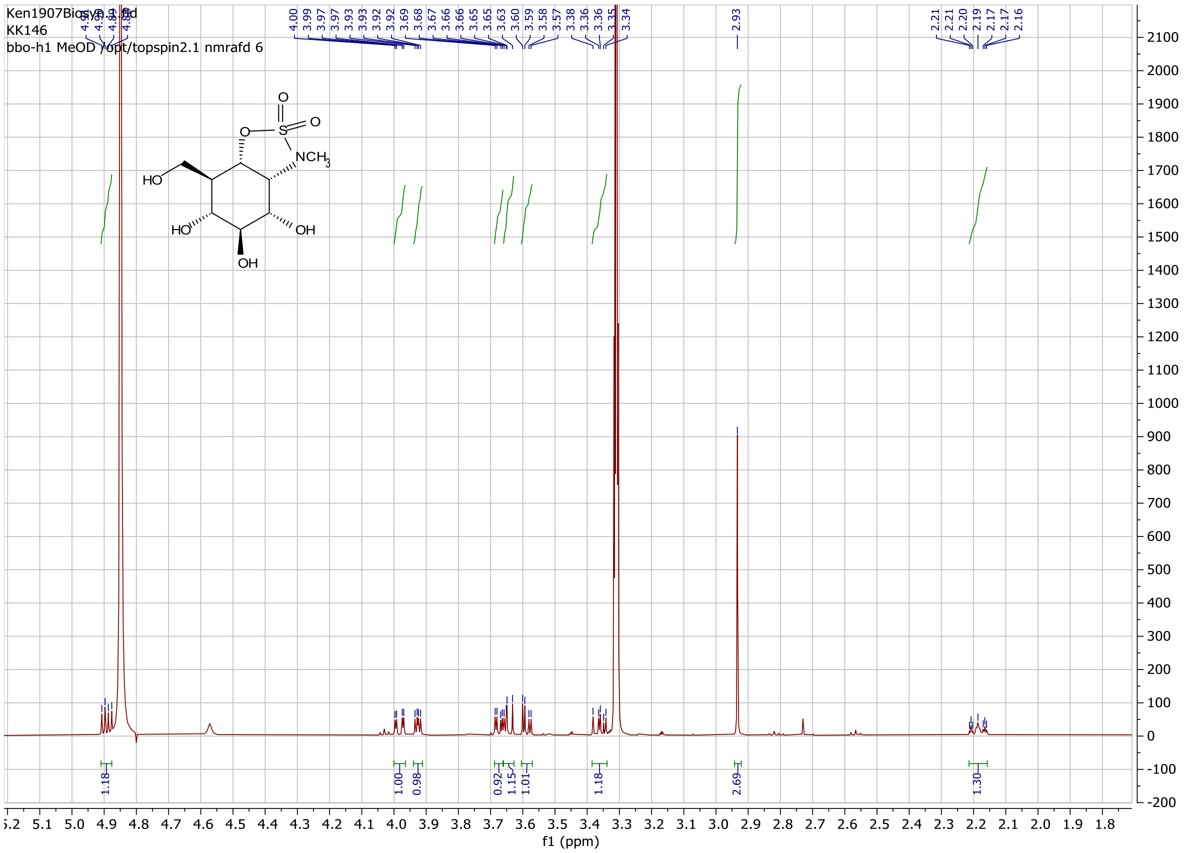

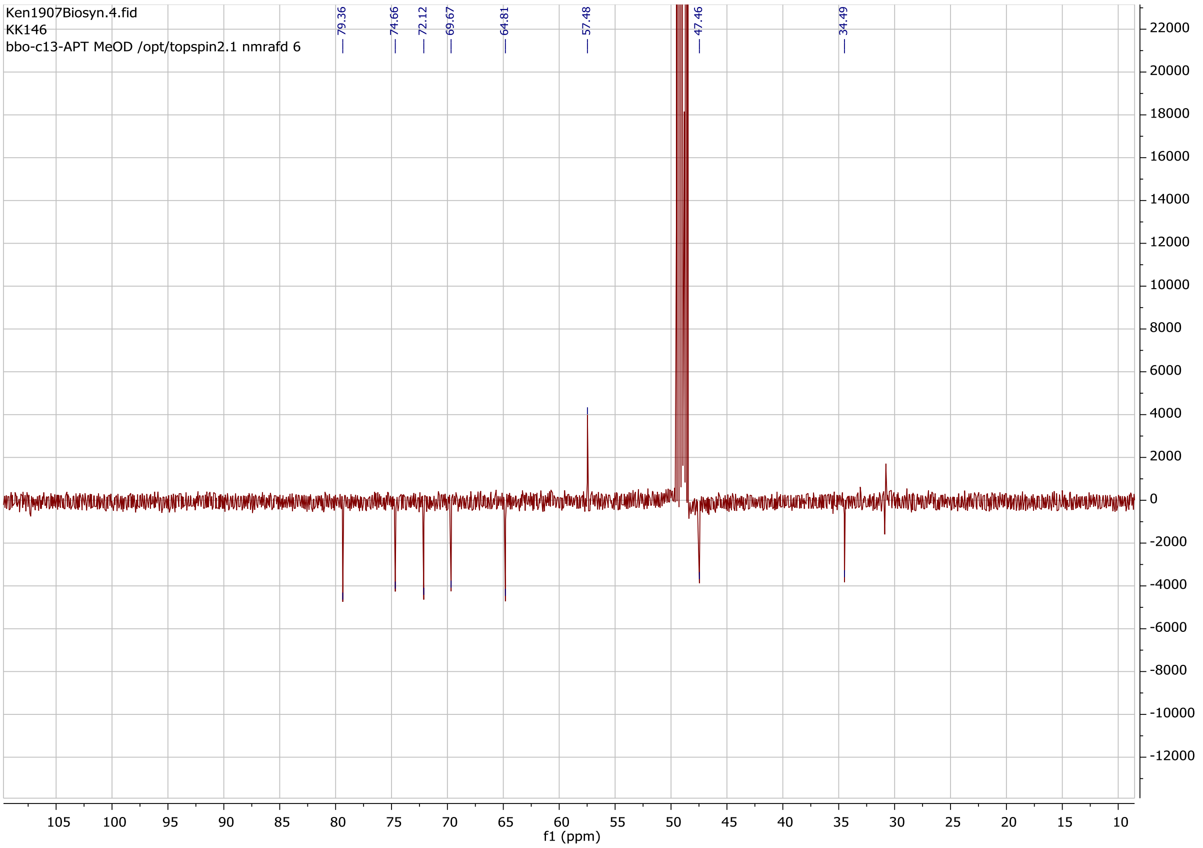

^{1}H-NMR and ^{13}C-NMR of **19** in $CDCl_3$:

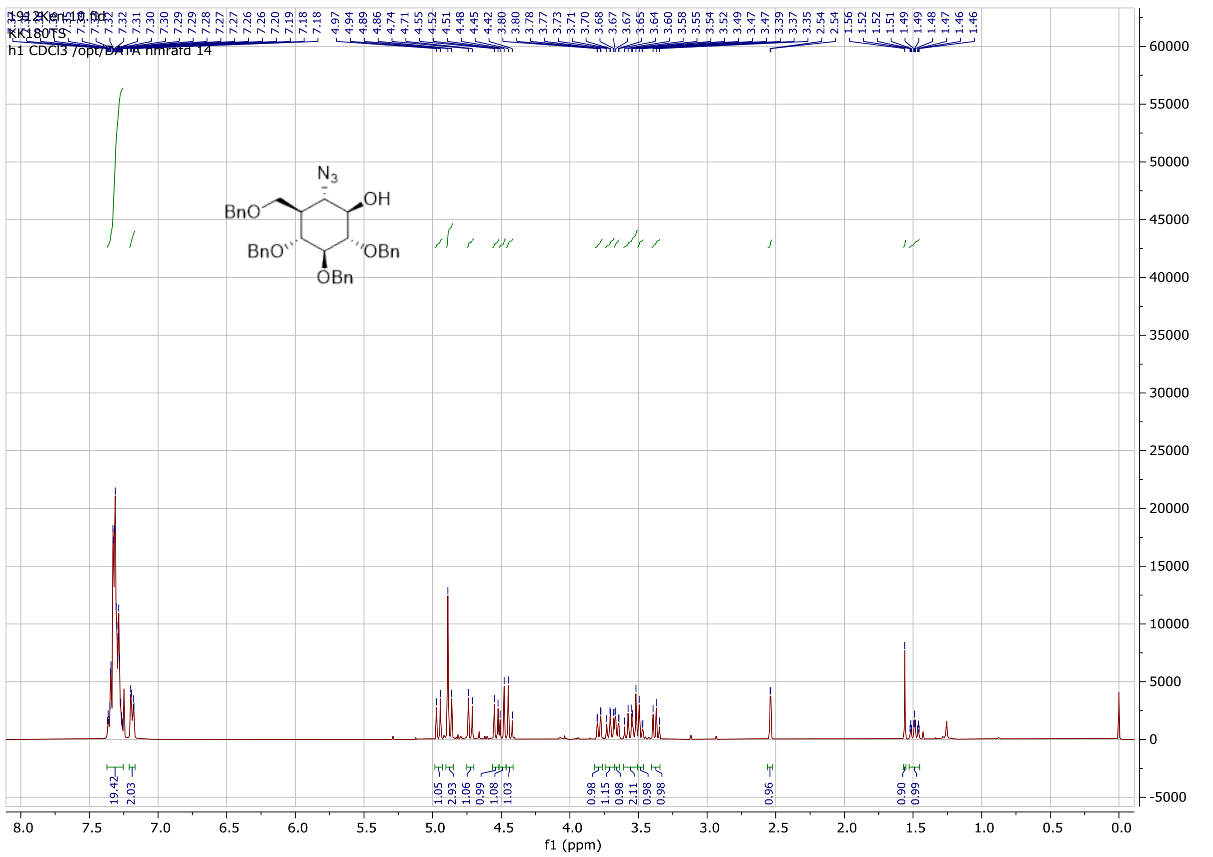

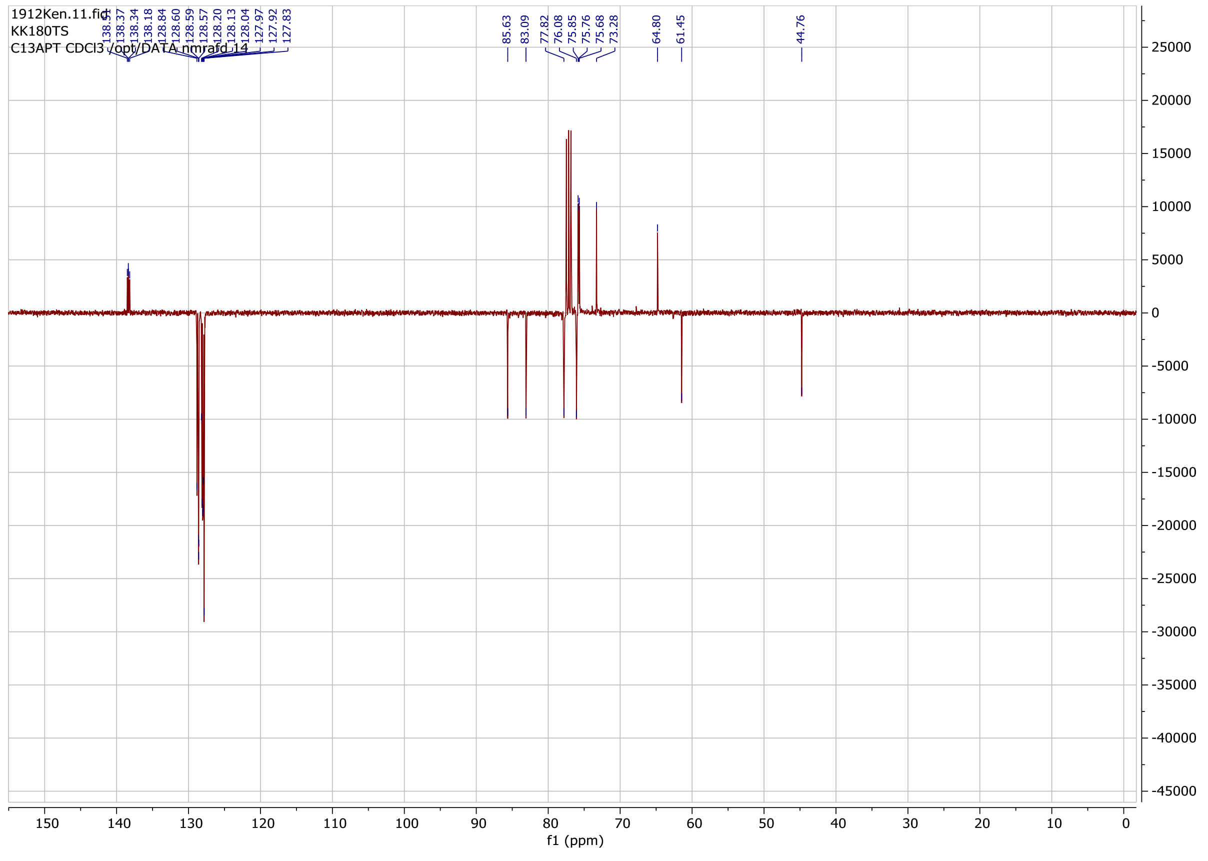

^{1}H-NMR and ^{13}C-NMR of **20** in $CDCl_3$:

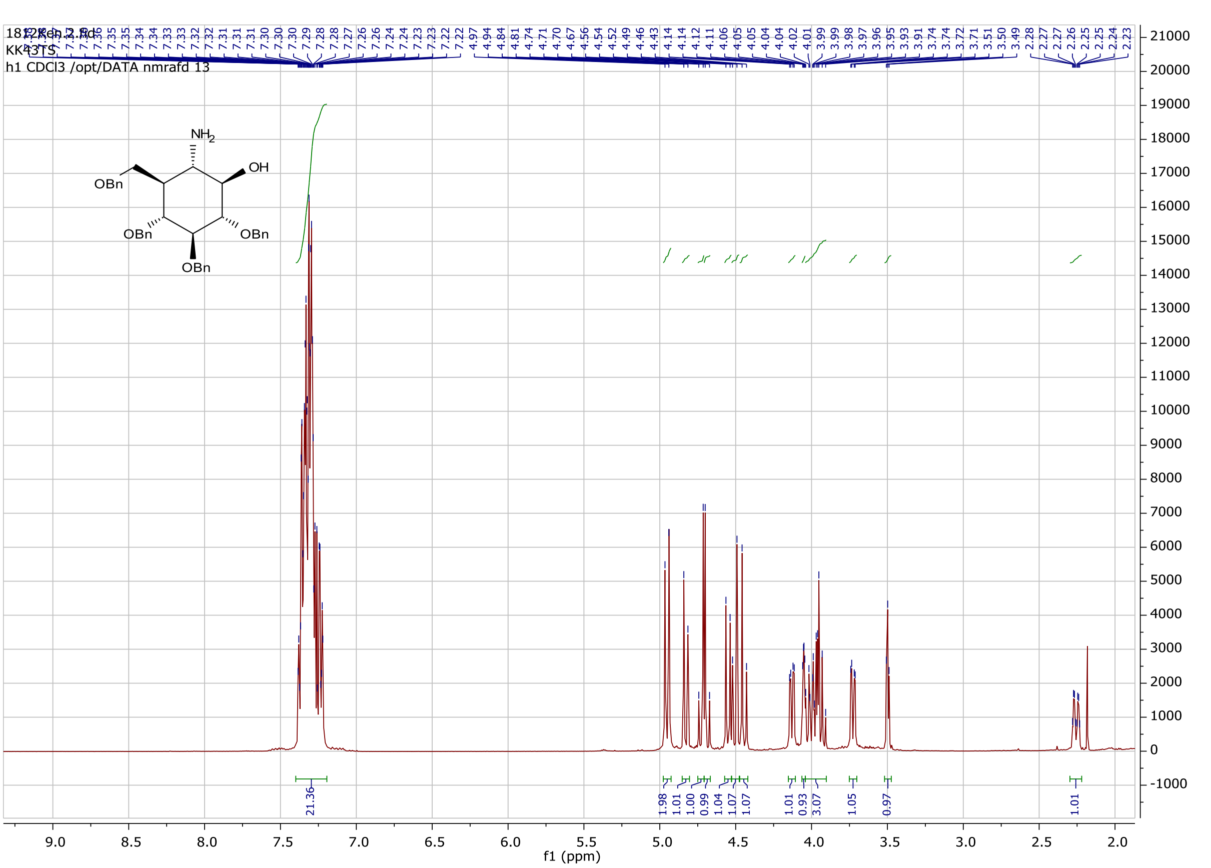

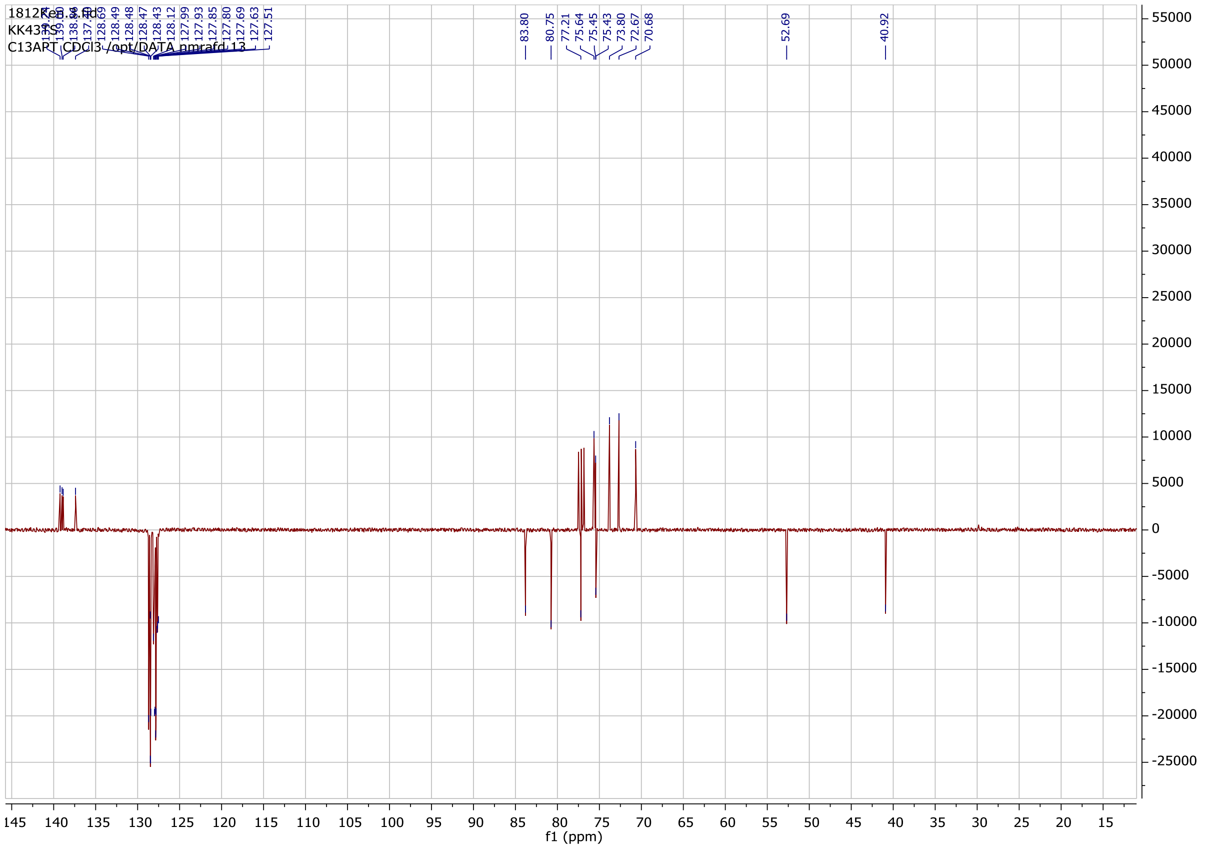

^{1}H-NMR and ^{13}C-NMR of **22** in $CDCl_3$:

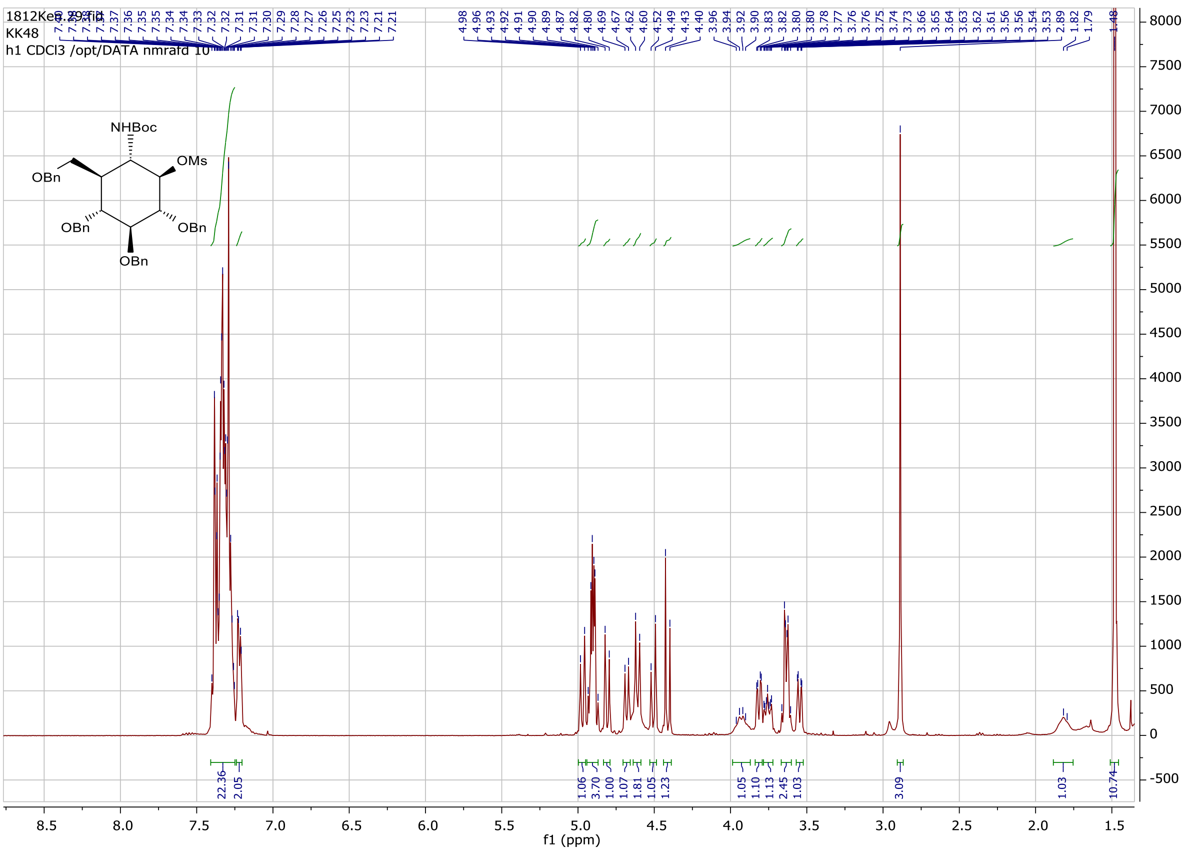

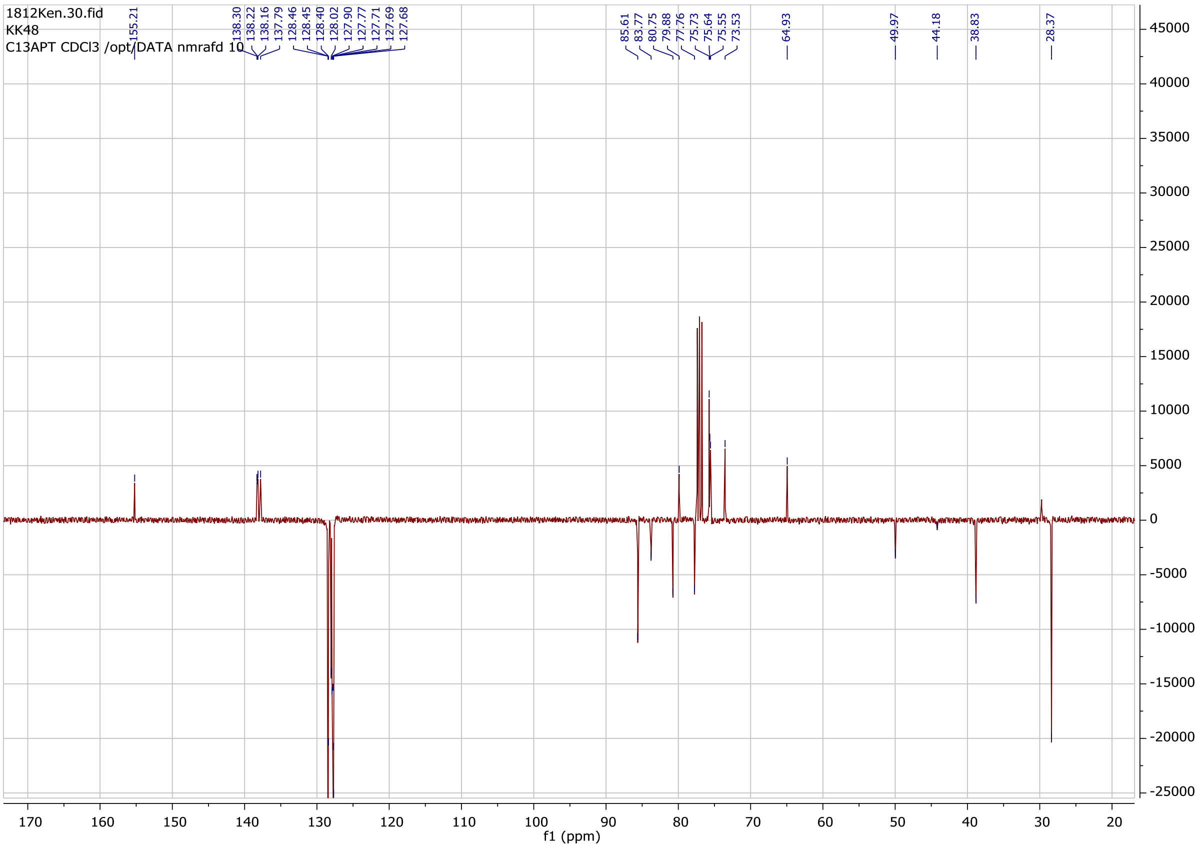

^{1}H-NMR and ^{13}C-NMR of **32** in $CDCl_3$:

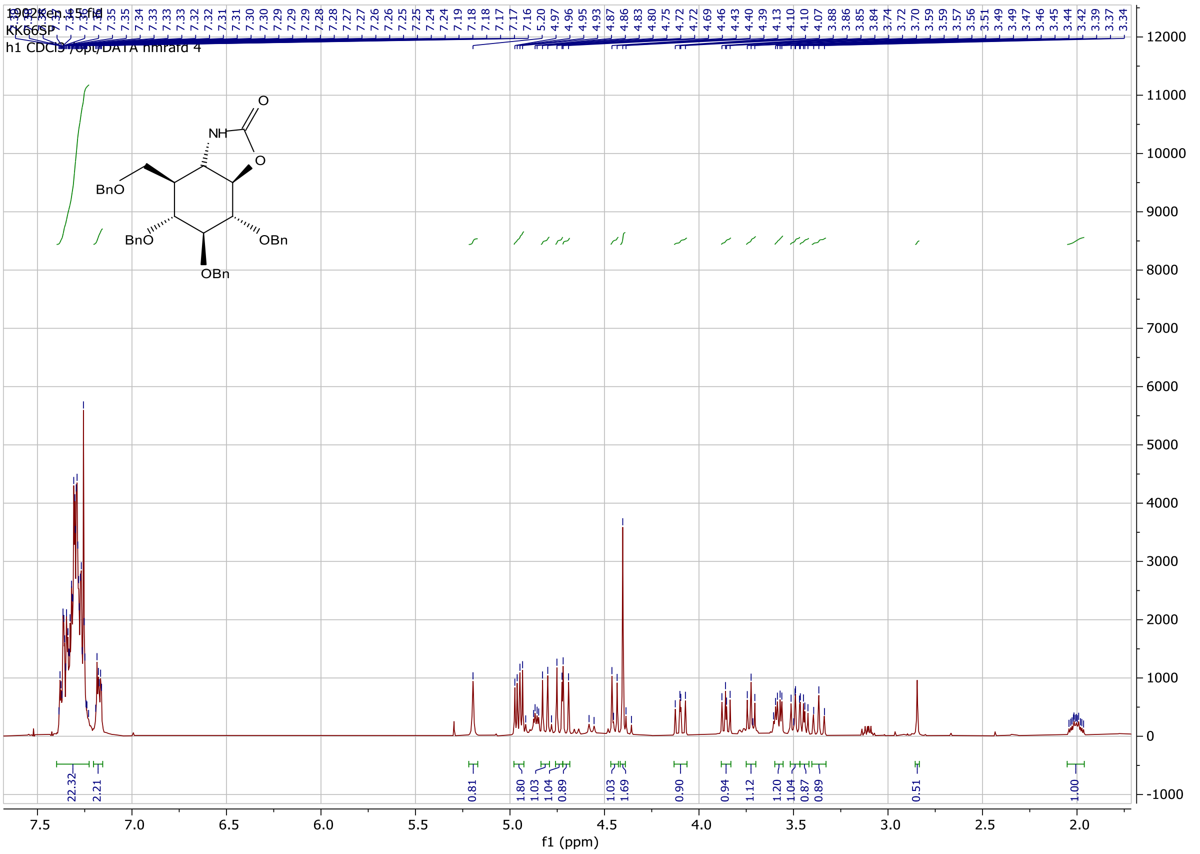

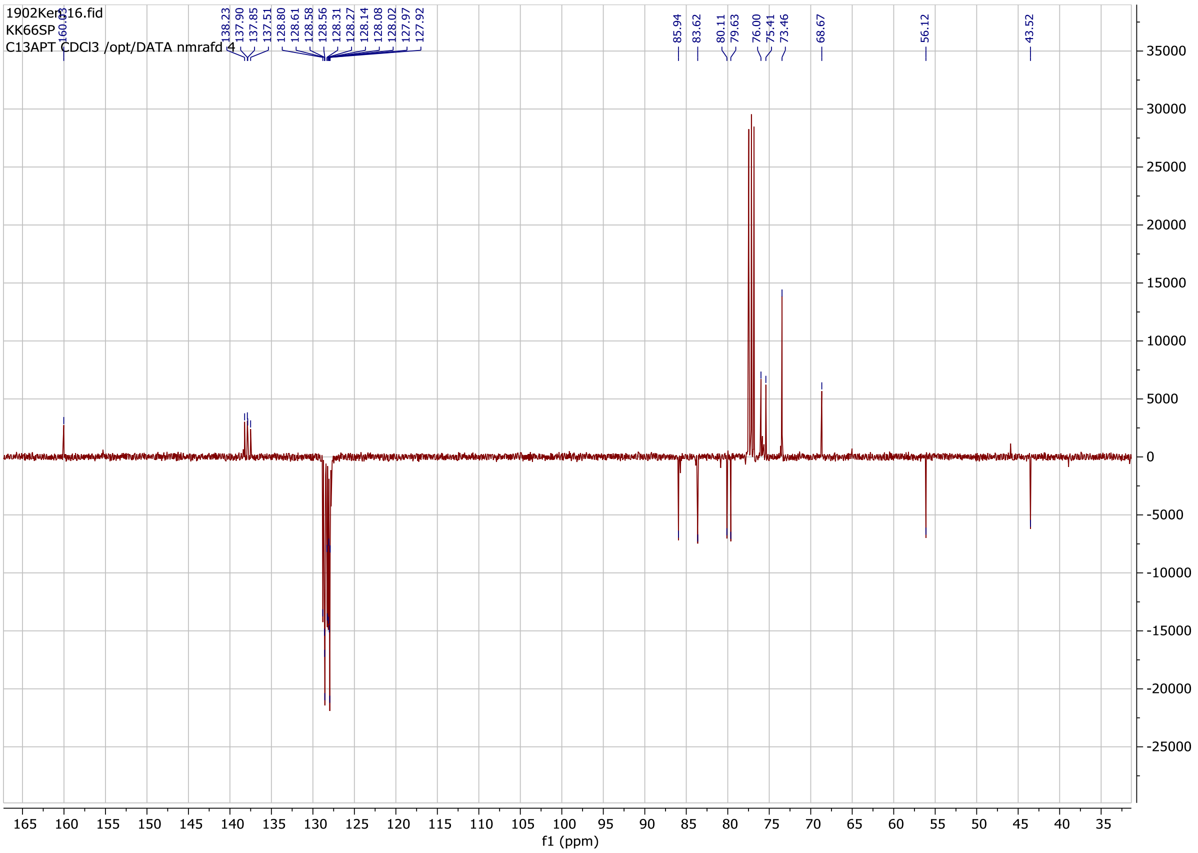

^{1}H-NMR and ^{13}C-NMR of **23** in $CDCl_3$:

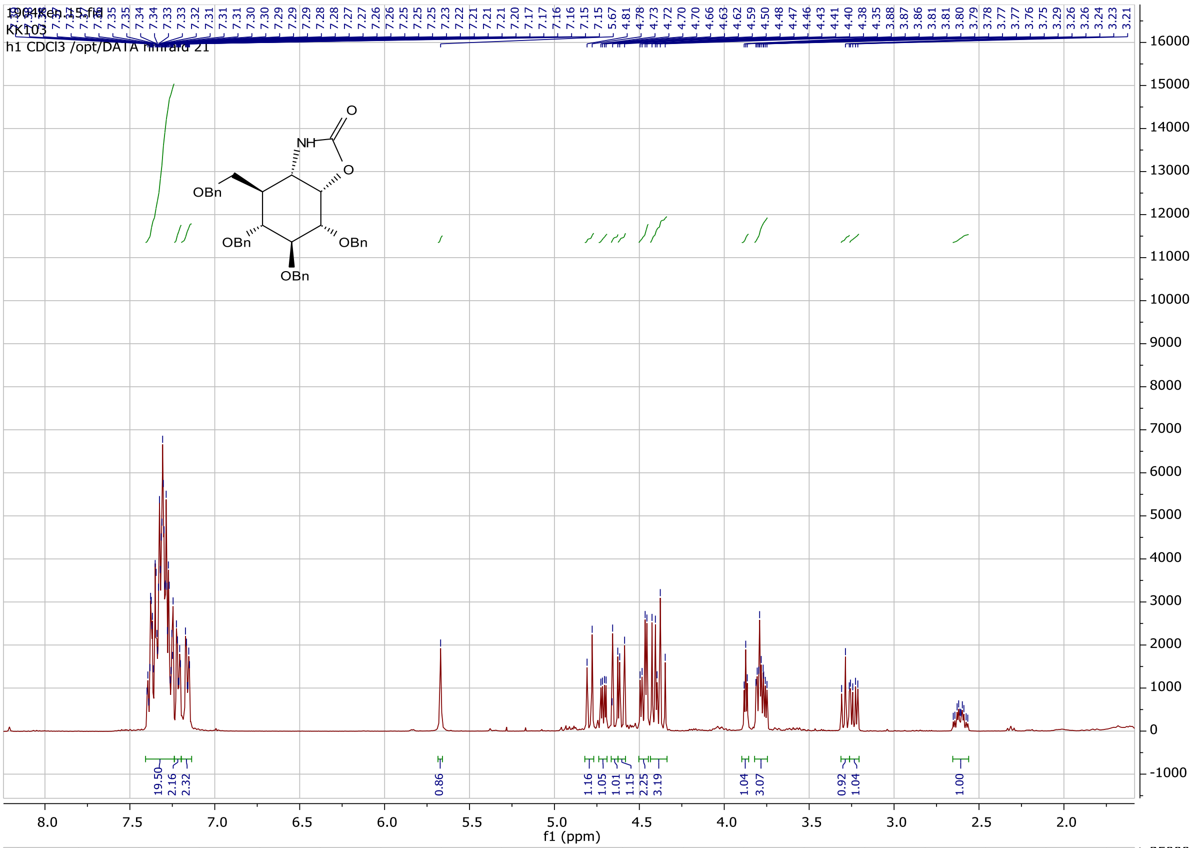

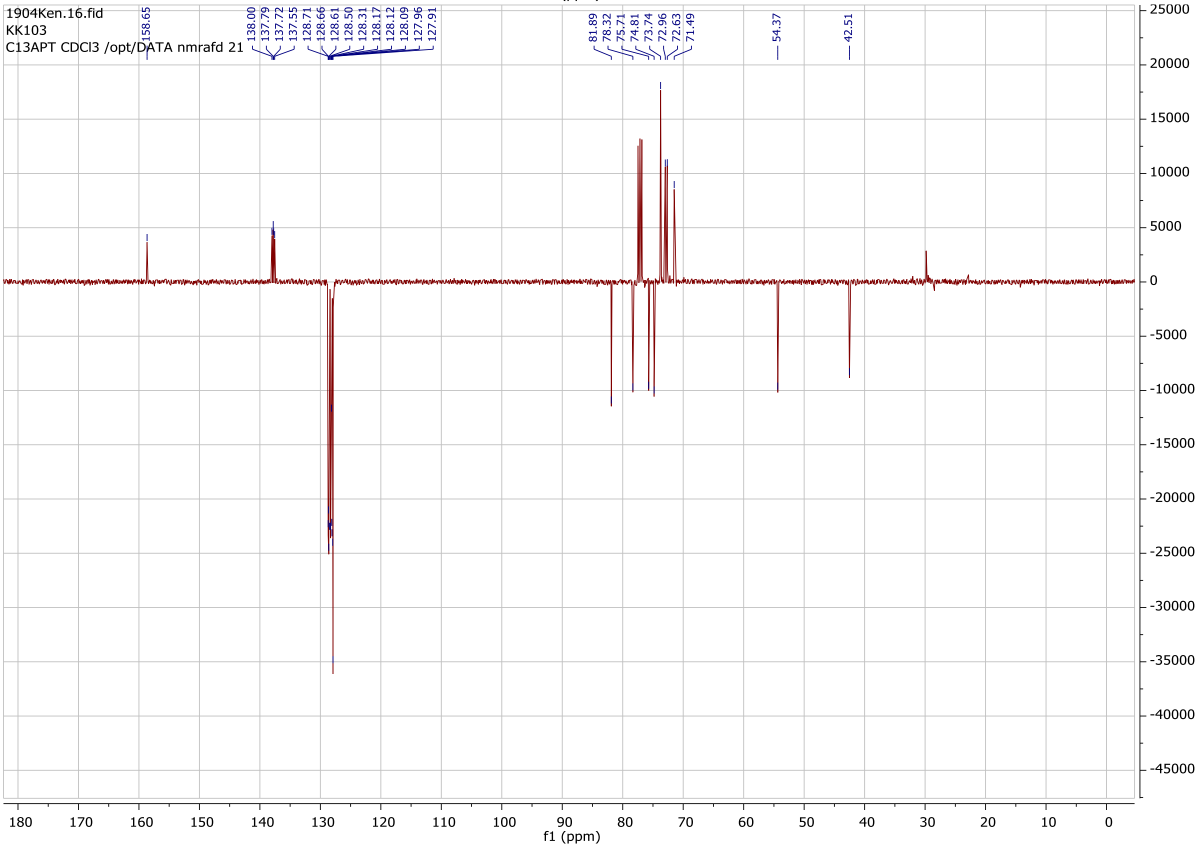

^{1}H-NMR and ^{13}C-NMR of **24** in $CDCl_3$:

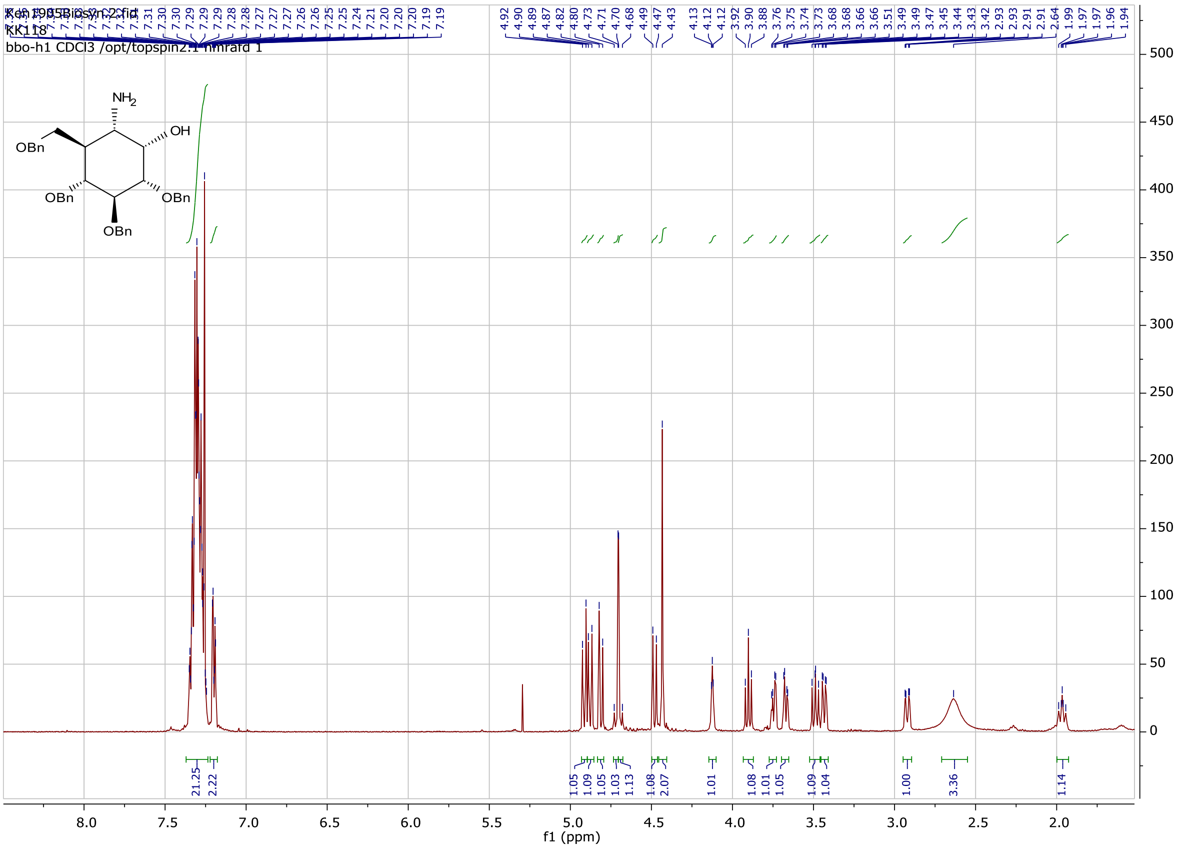

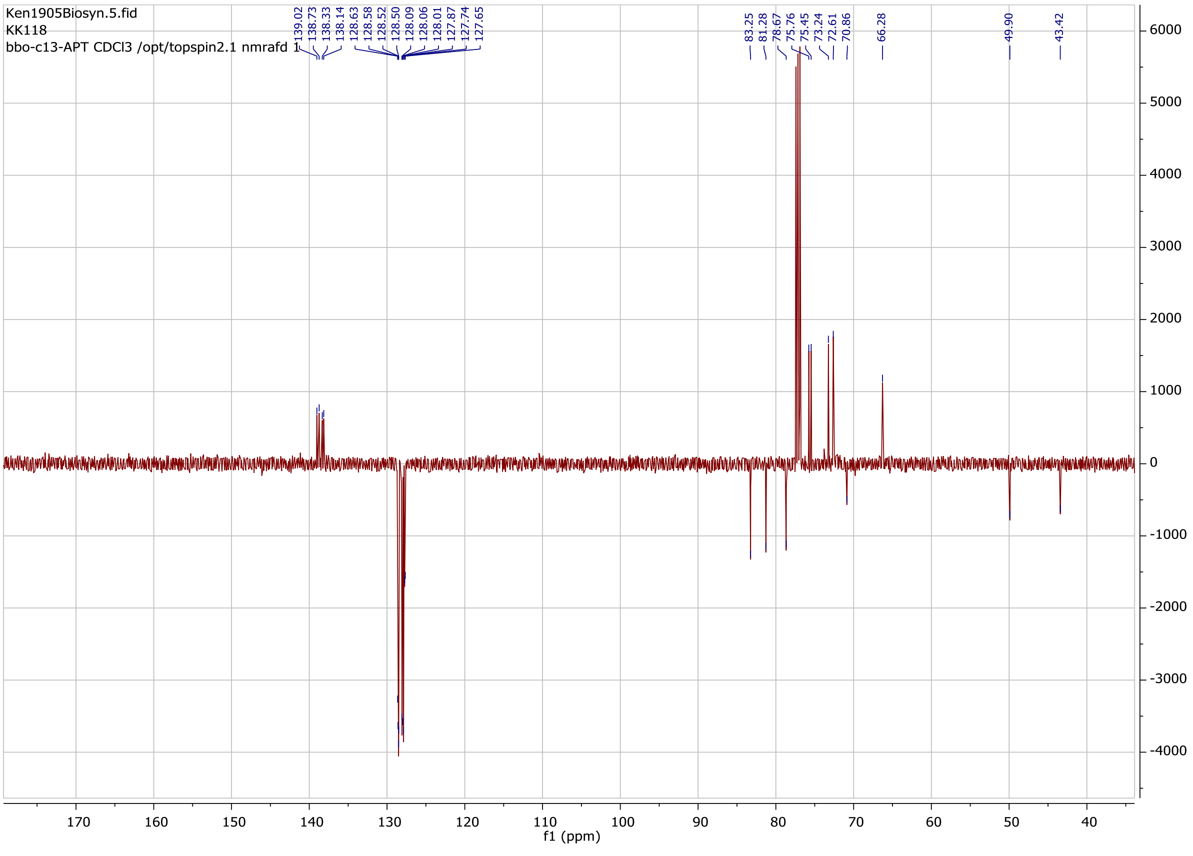

^{1}H-NMR and ^{13}C-NMR of **25** in $CDCl_3$:

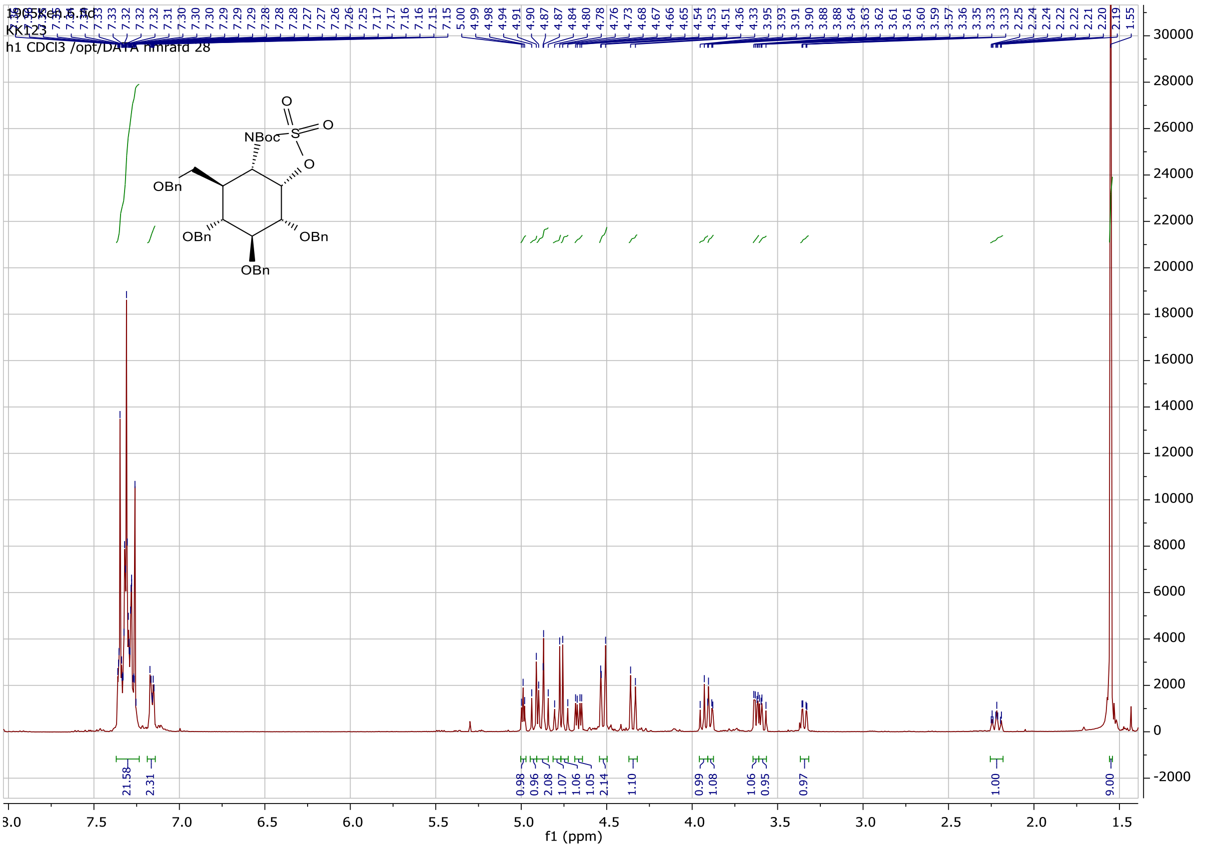

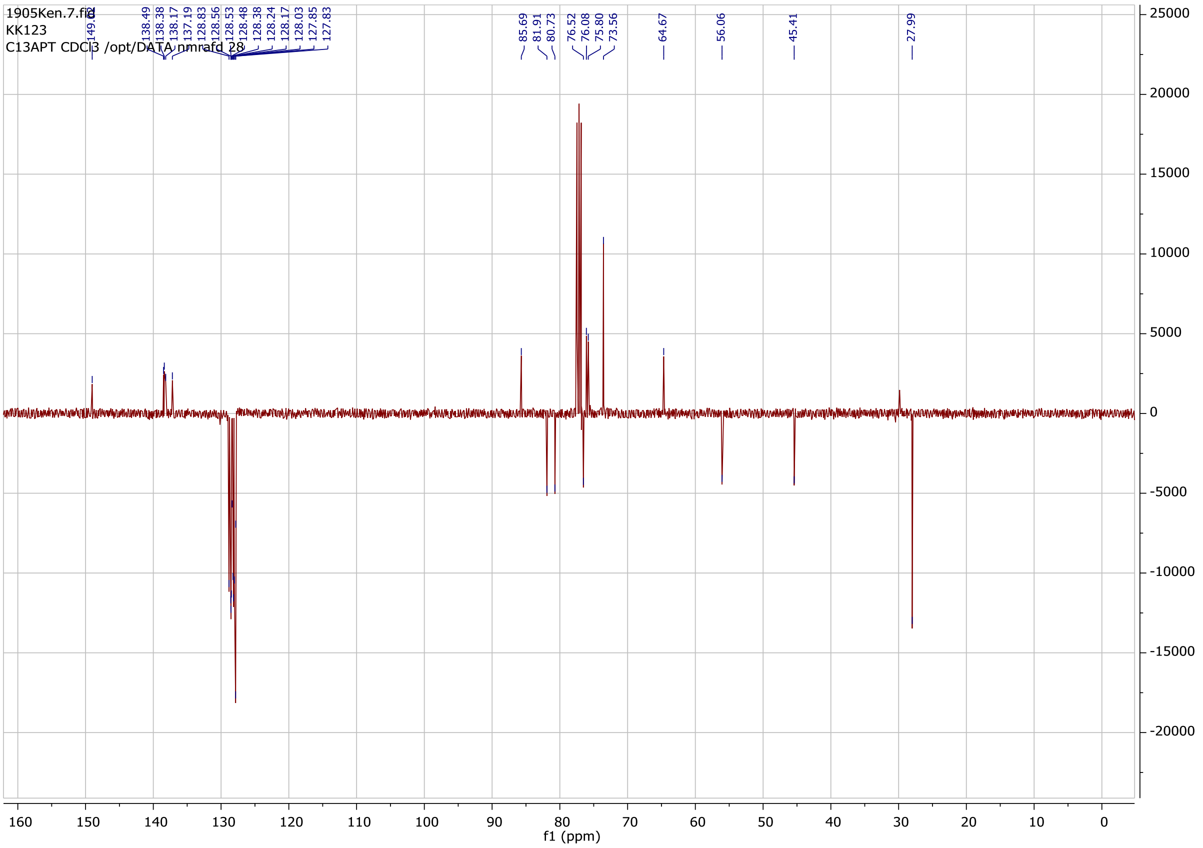

^1H-NMR and ^{13}C-NMR of **26** in $CDCl_3$:

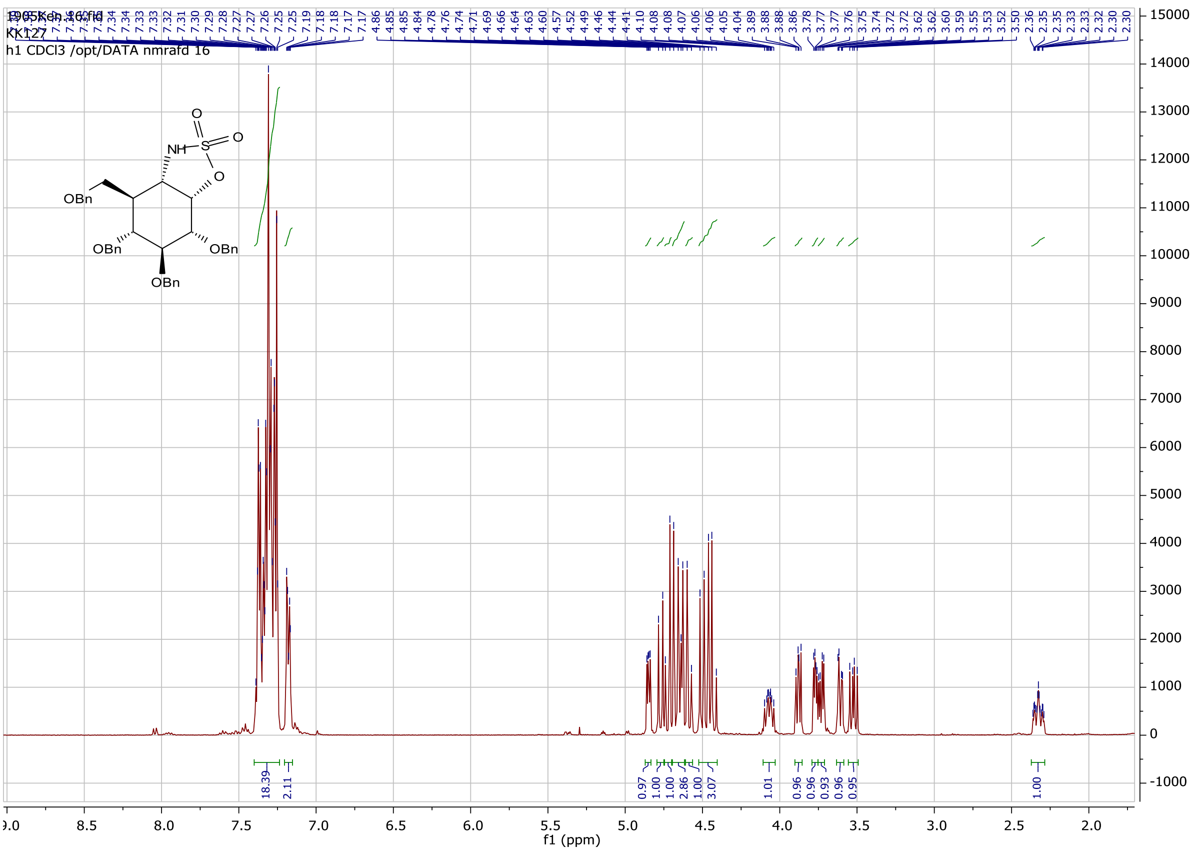

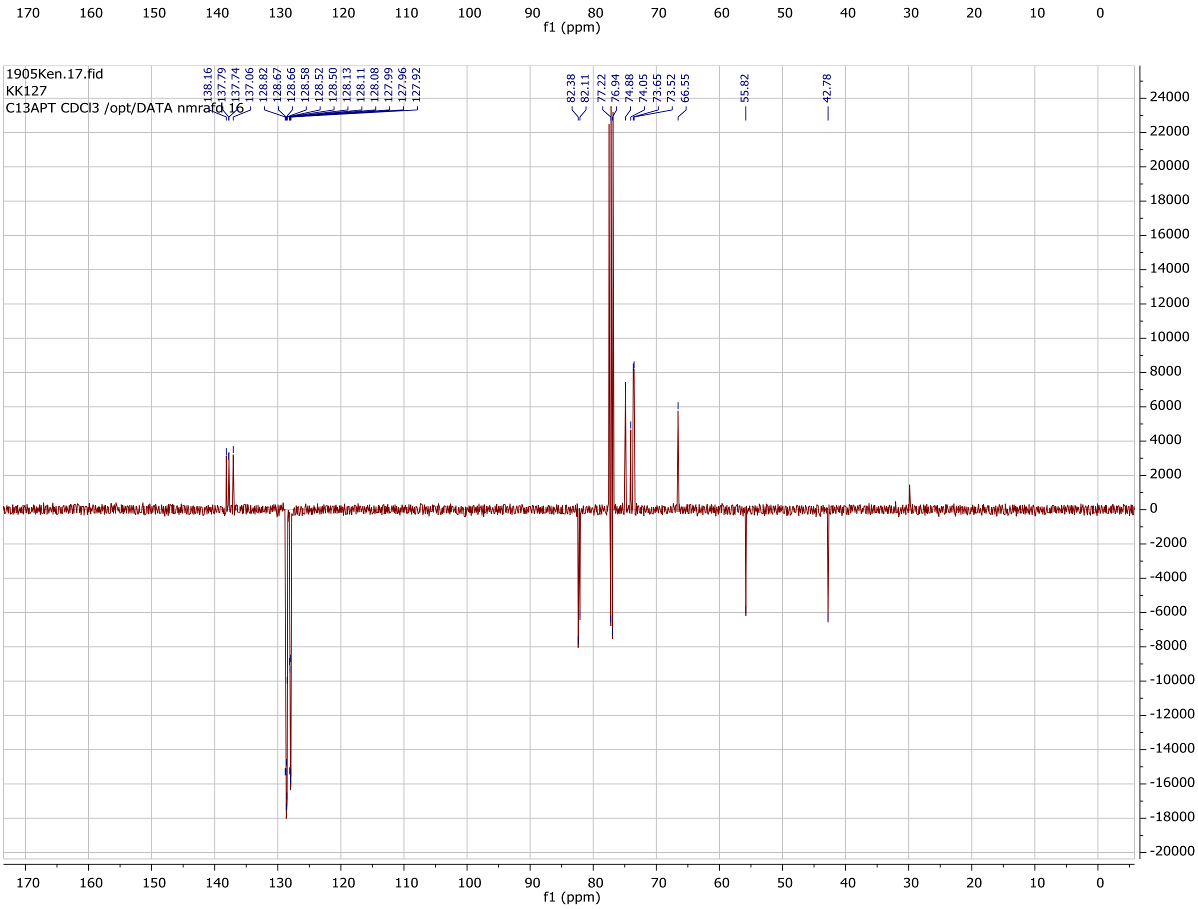

^{1}H-NMR and ^{13}C-NMR of **27** in $CDCl_3$:

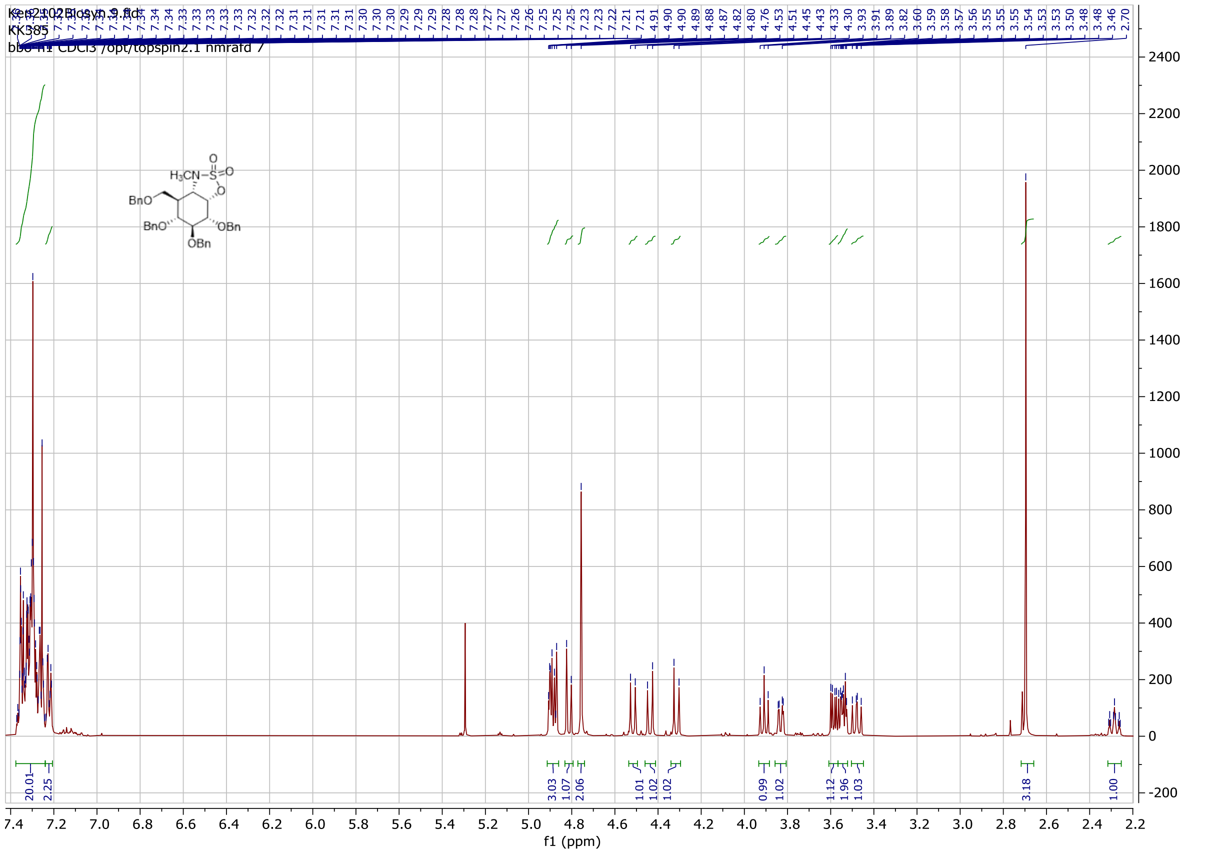

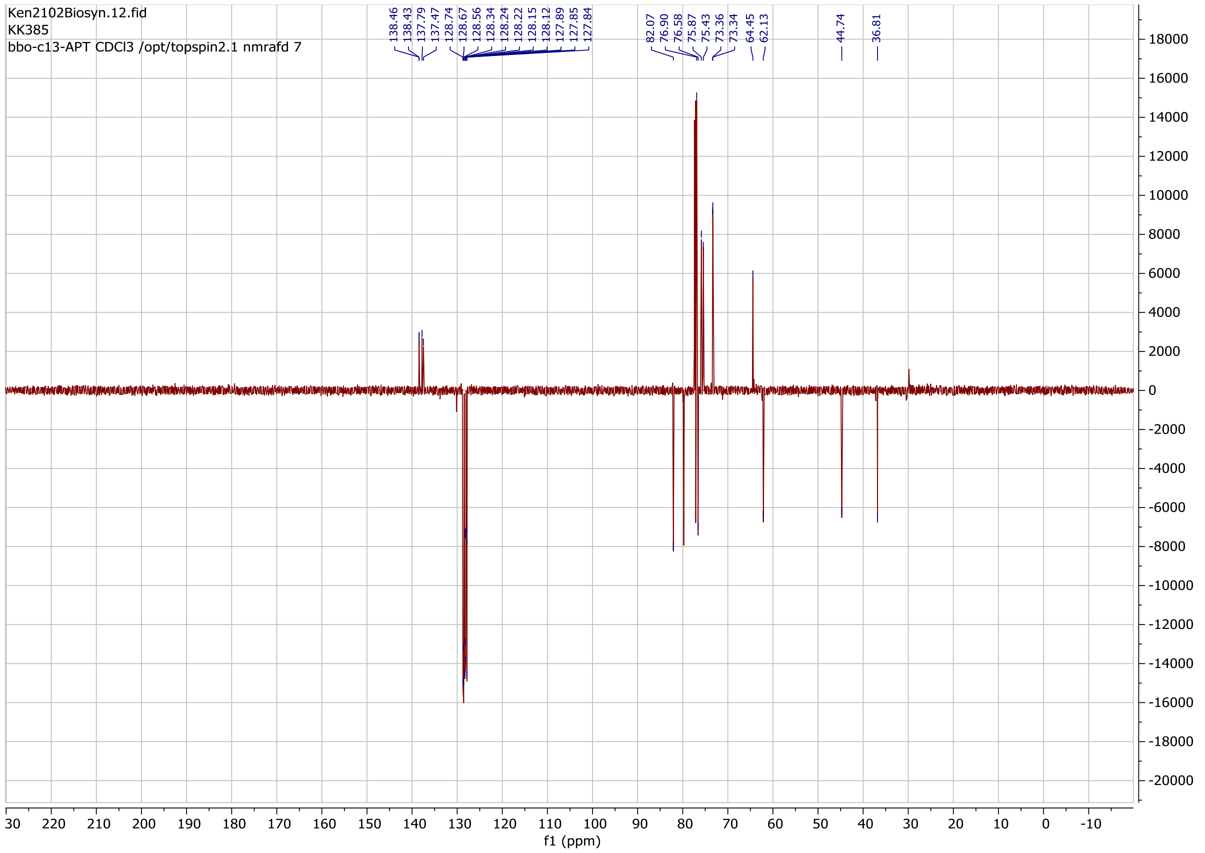

^{1}H-NMR and ^{13}C-NMR spectra of **6** in MeOD

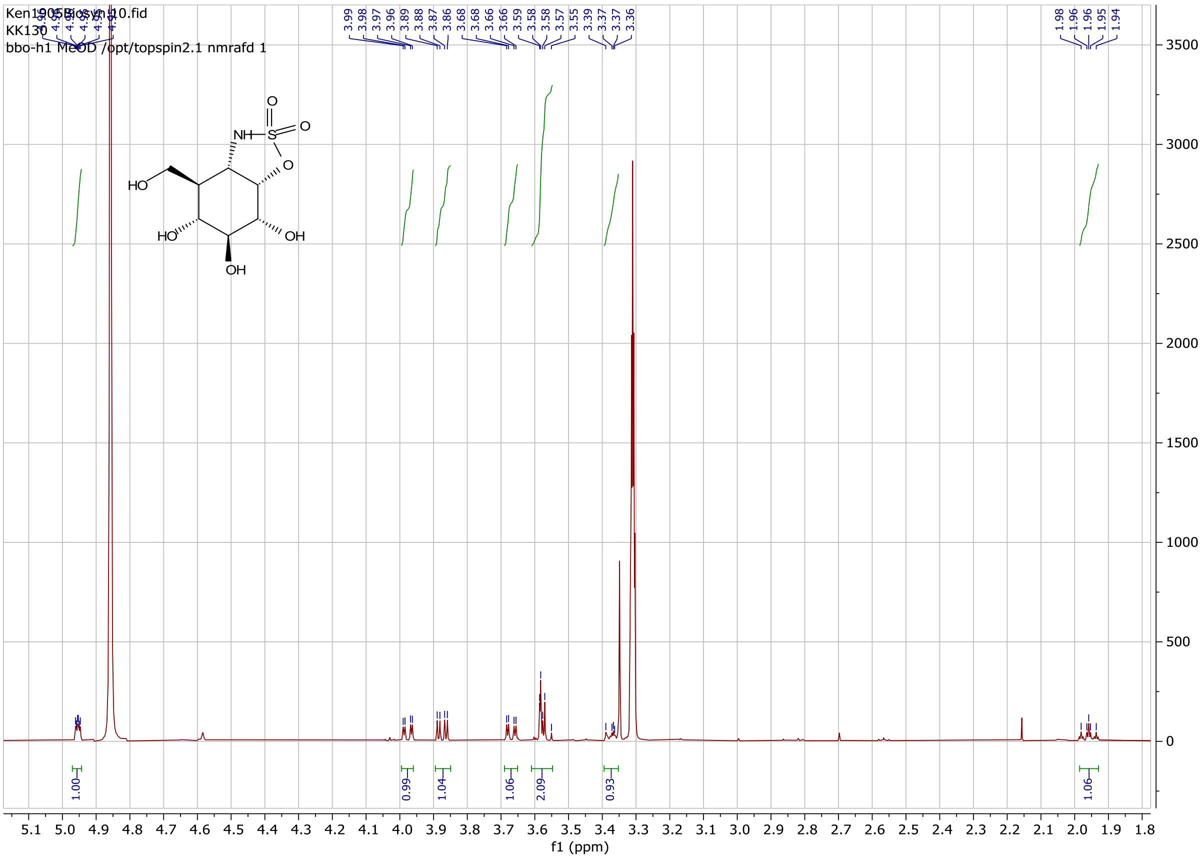

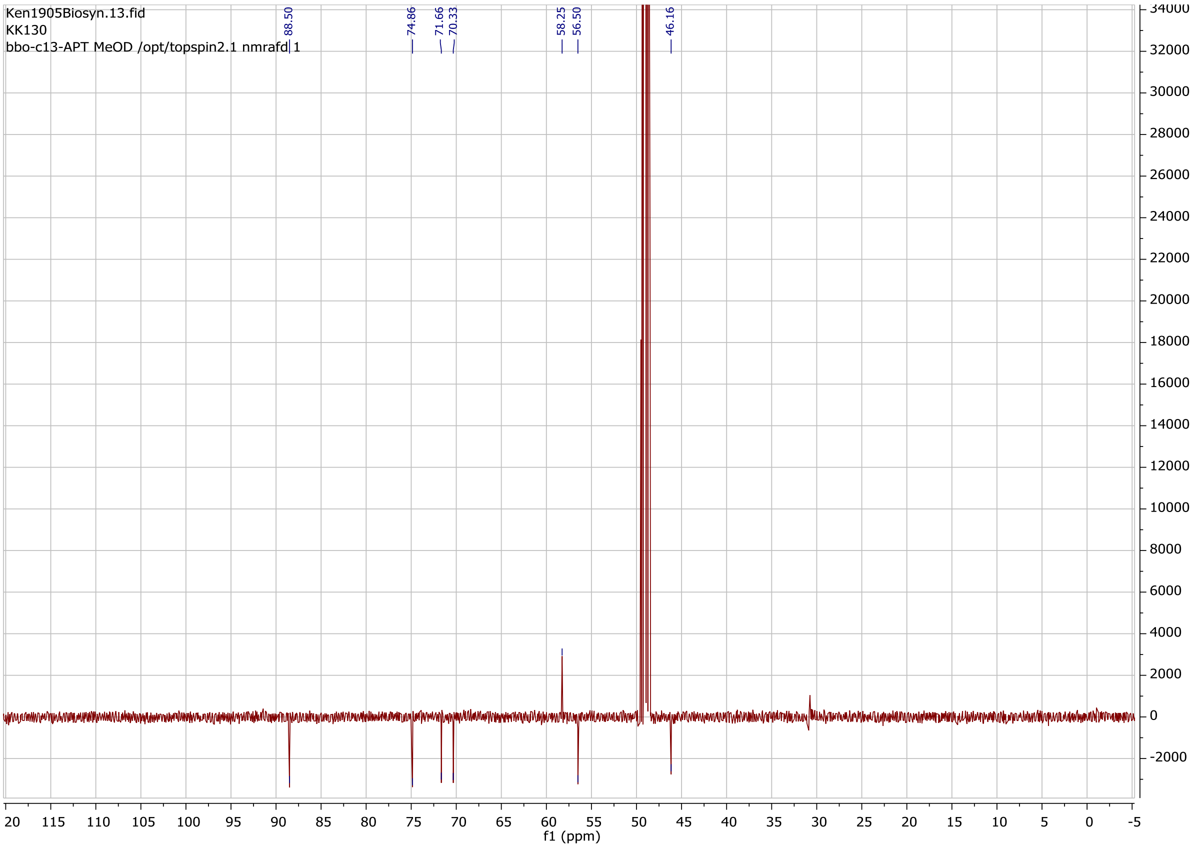

^{1}H-NMR and ^{13}C-NMR spectra of **7** in MeOD

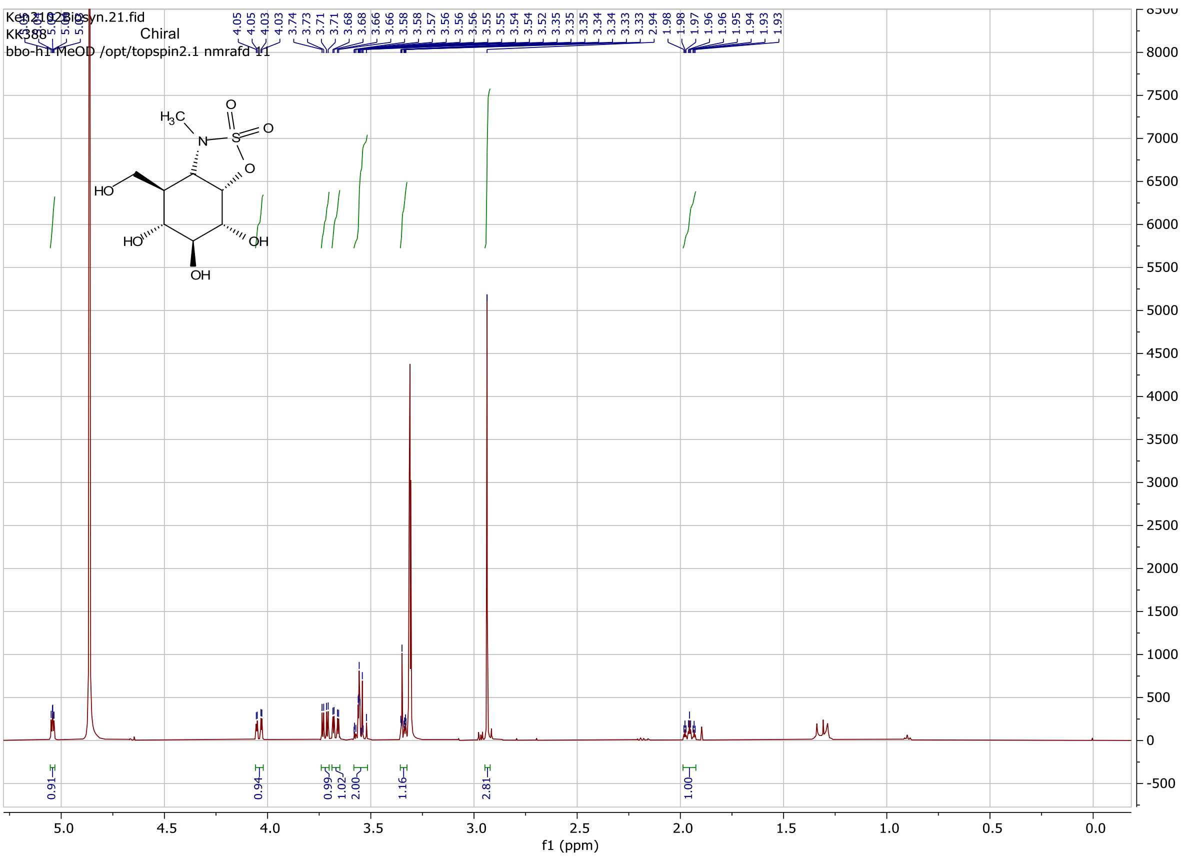

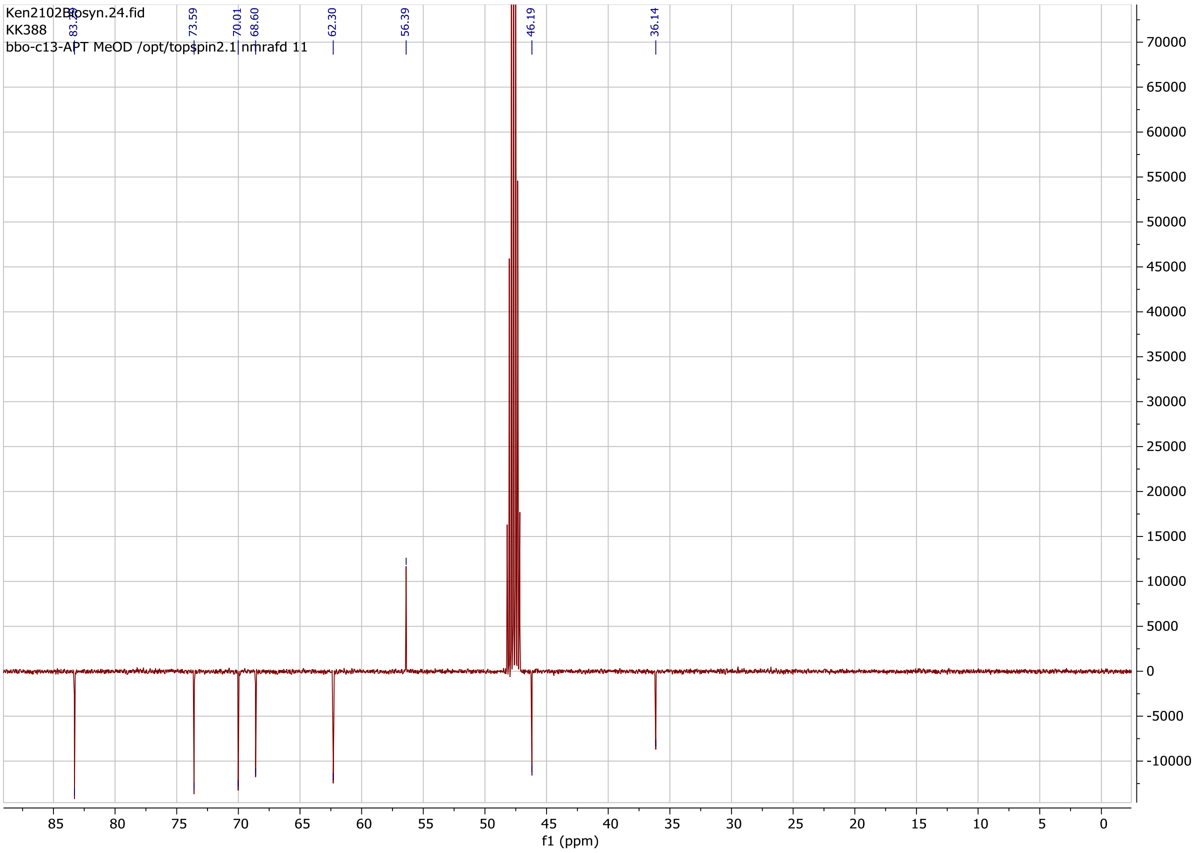

^{1}H-NMR and ^{13}C-NMR of **33** in $CDCl_3$:

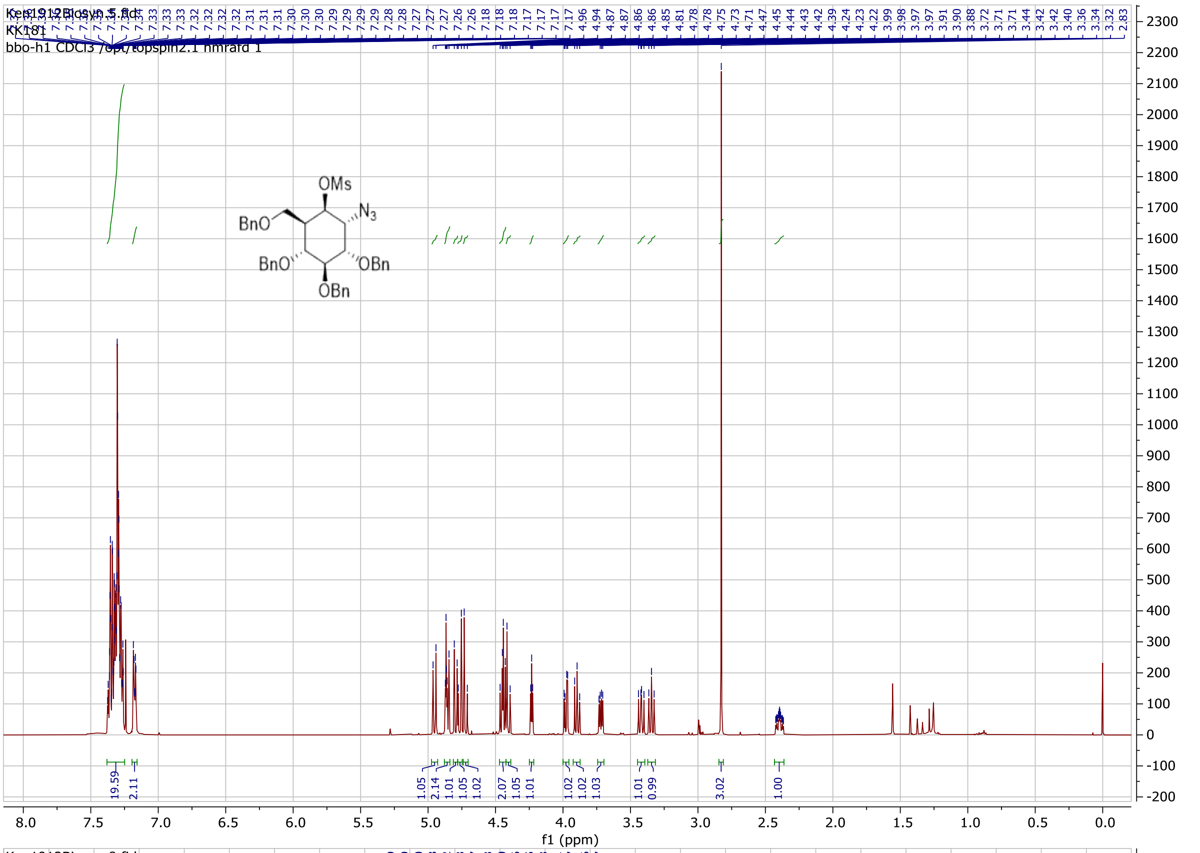

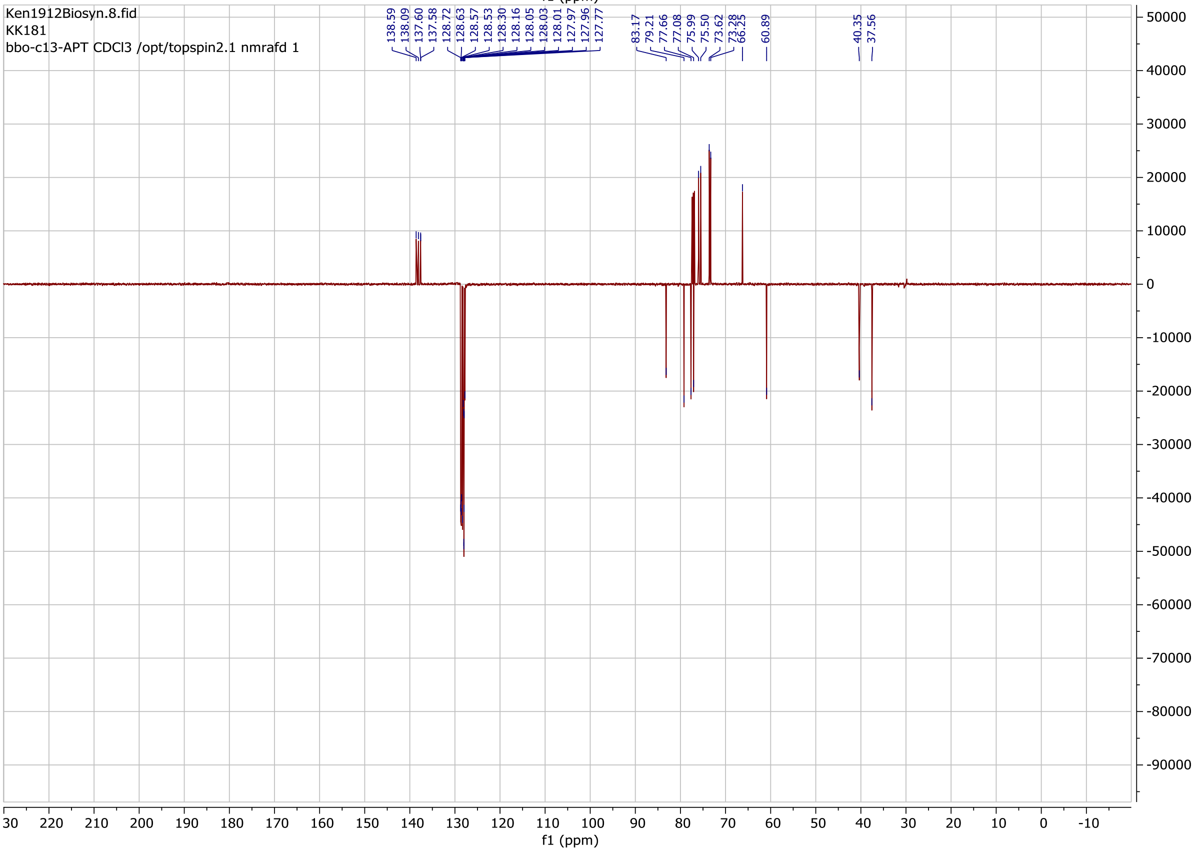

^{1}H-NMR and ^{13}C-NMR of **28** in $CDCl_3$:

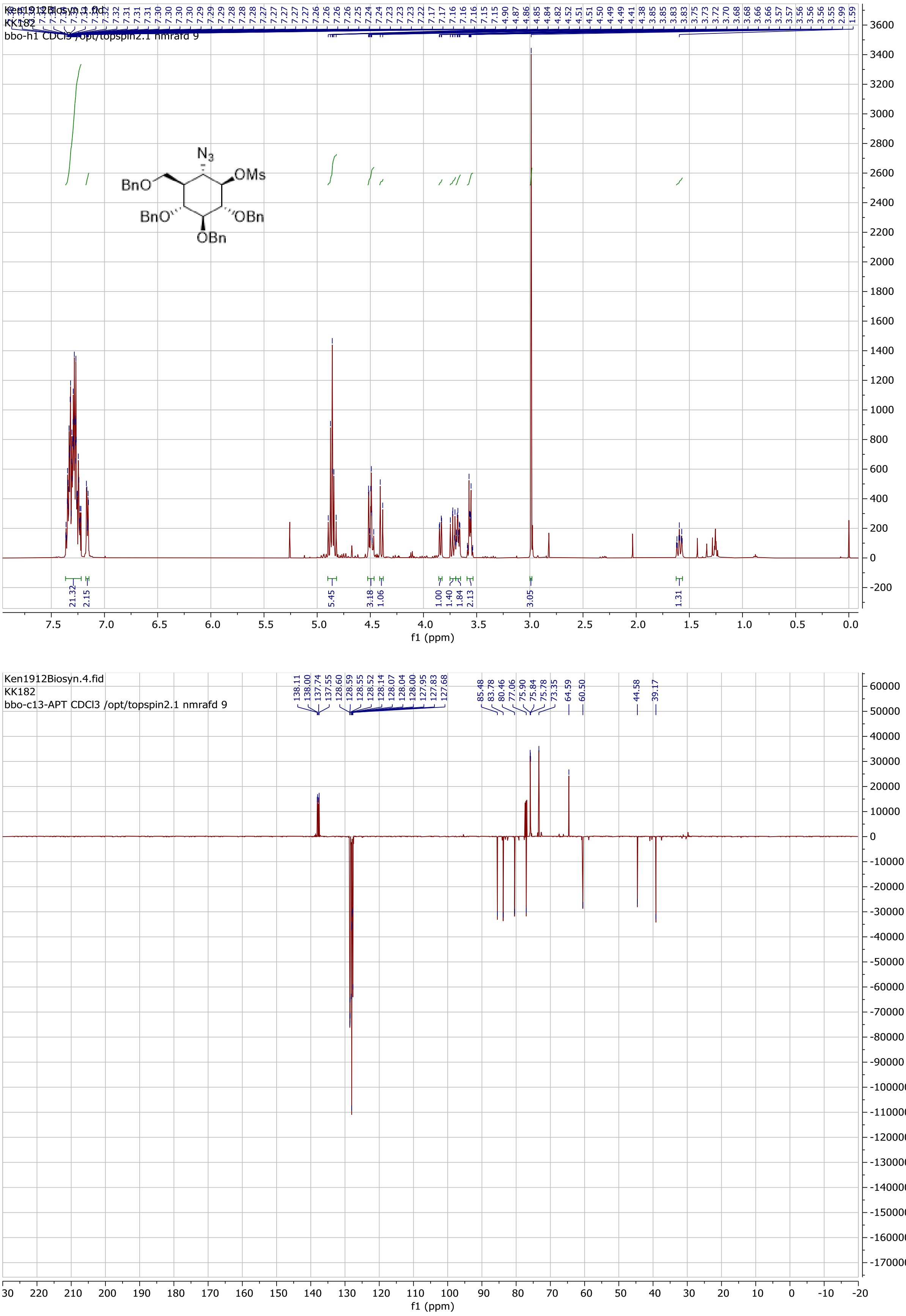

^{1}H-NMR and ^{13}C-NMR of **29** in $CDCl_3$:

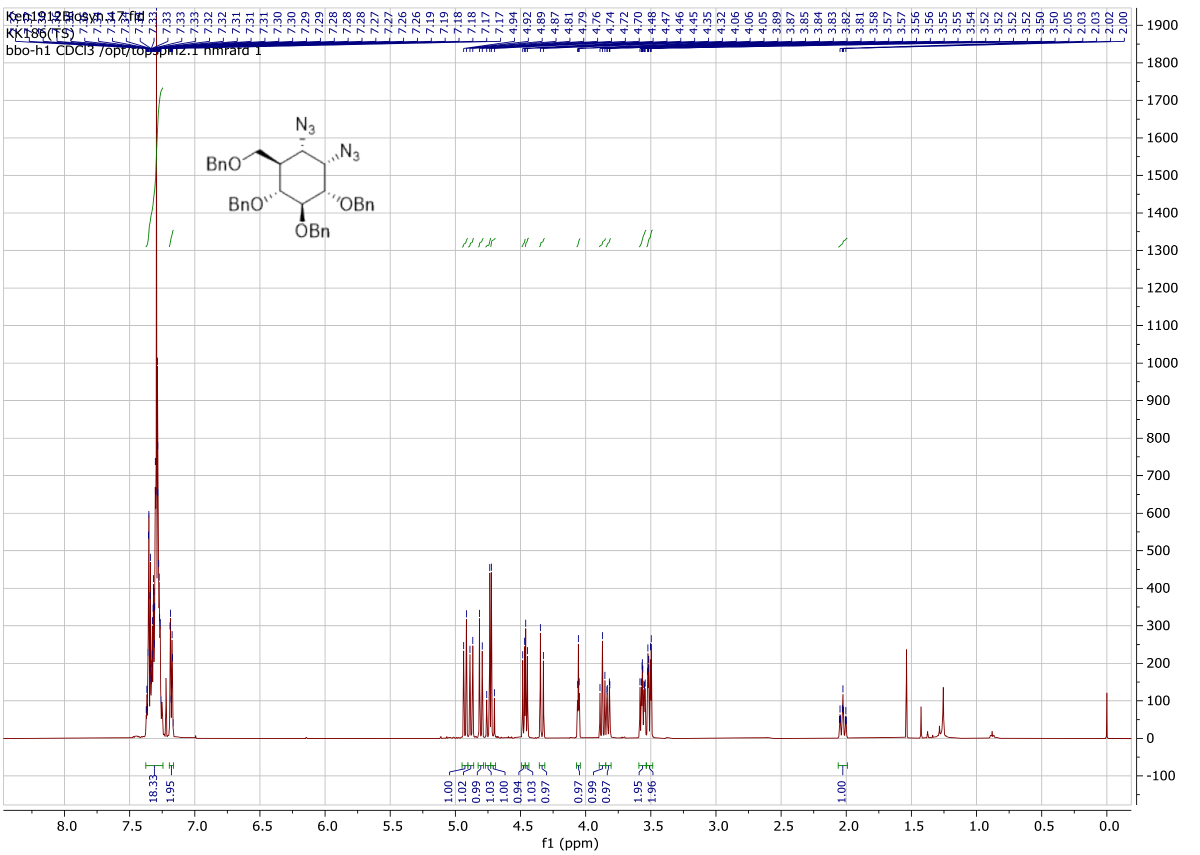

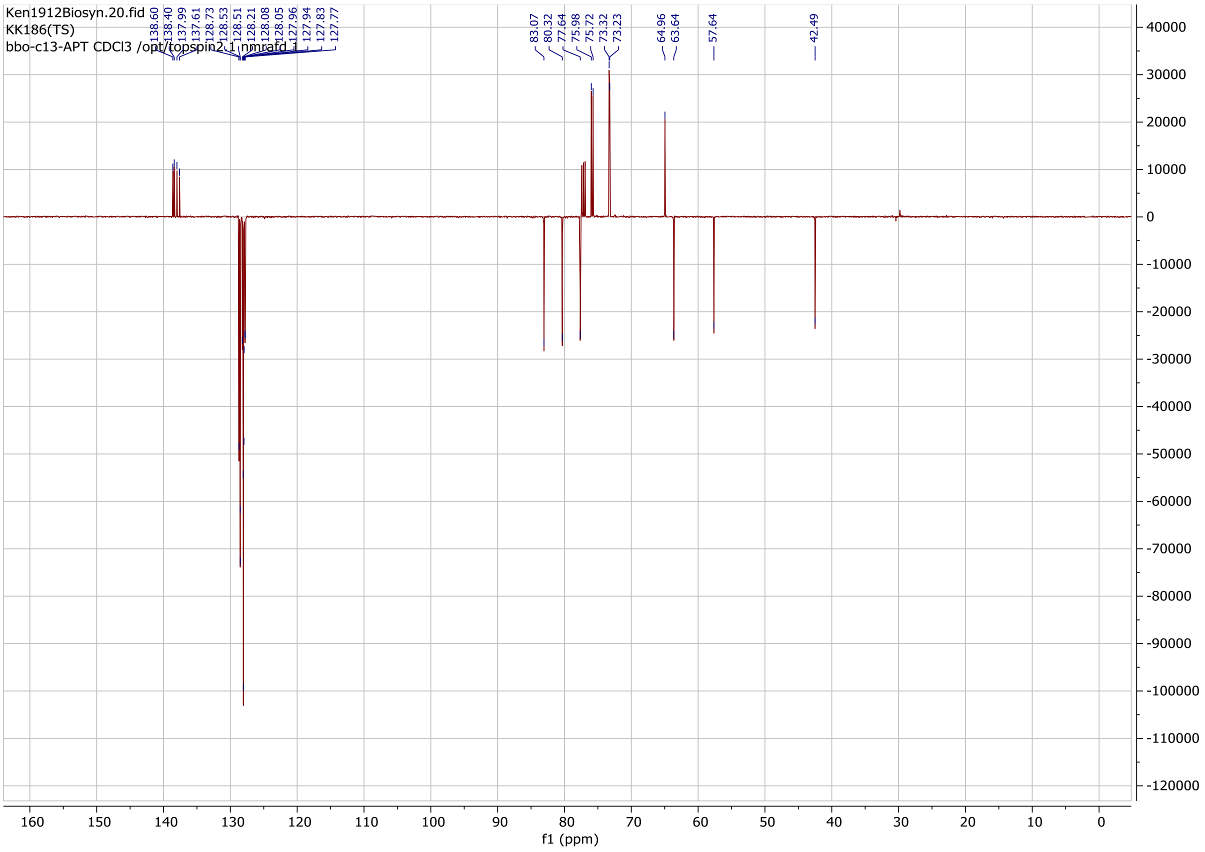

^{1}H-NMR and ^{13}C-NMR of **30** in $CDCl_3$:

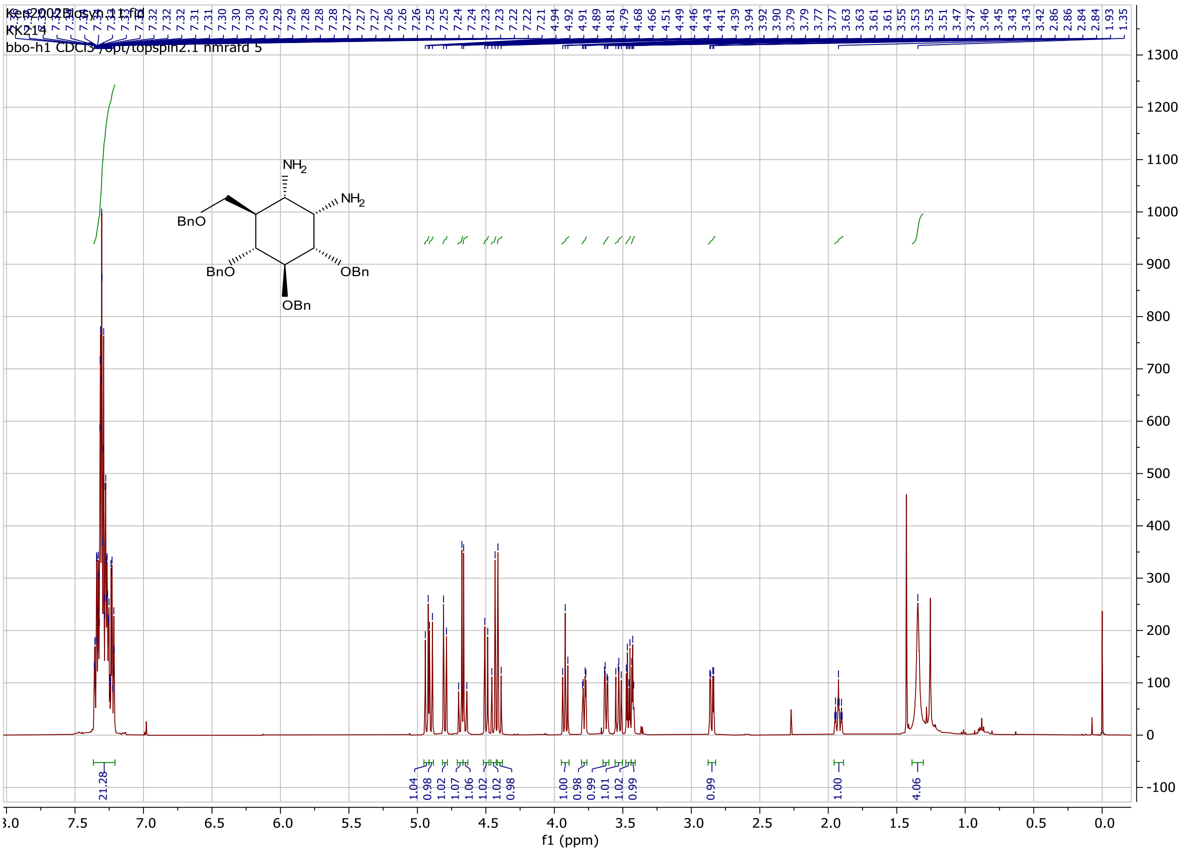

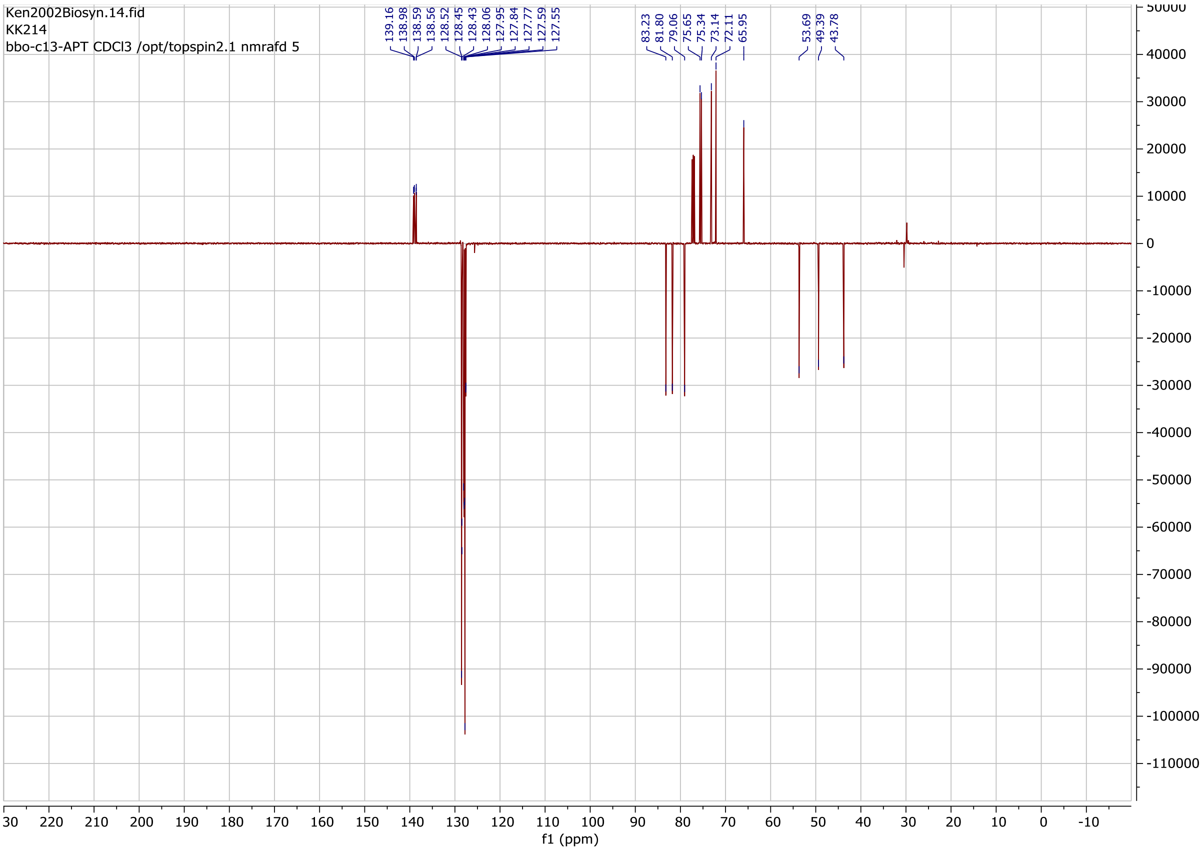

^{1}H-NMR and ^{13}C-NMR of **31** in $CDCl_3$:

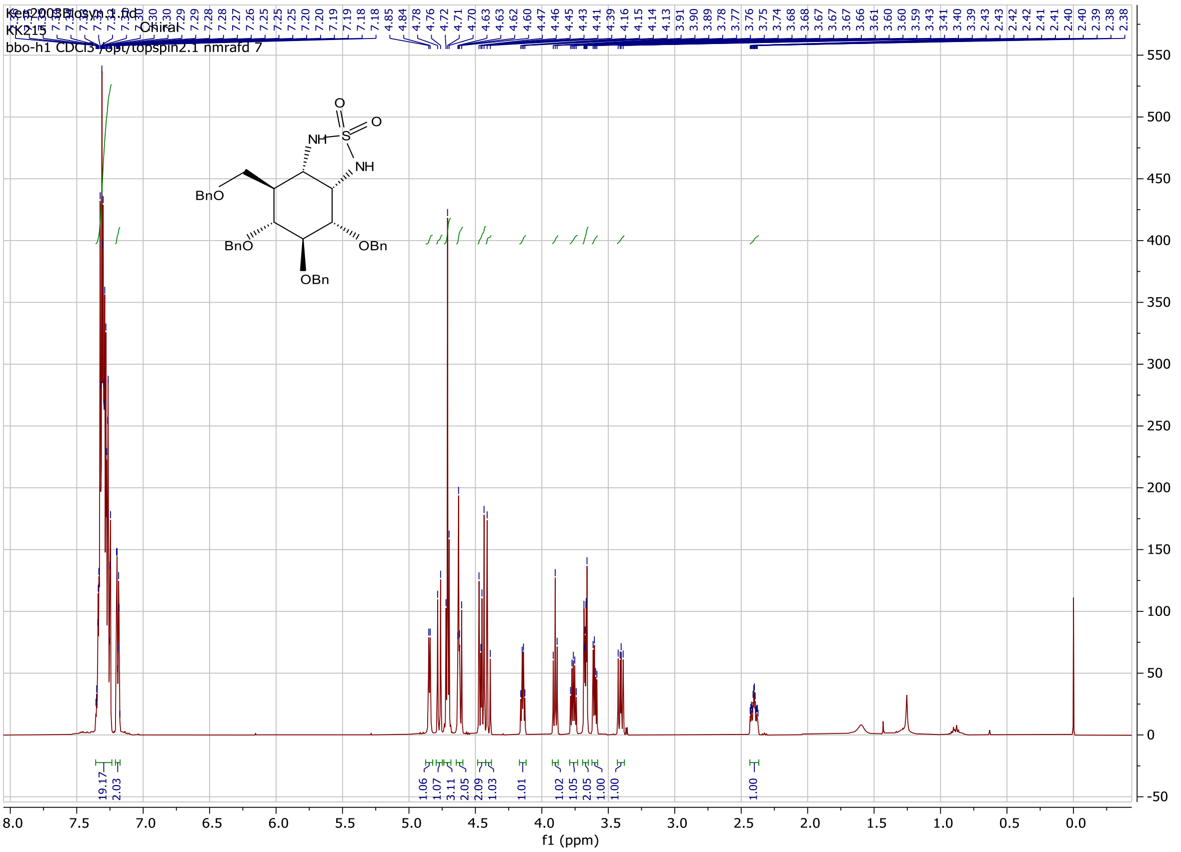

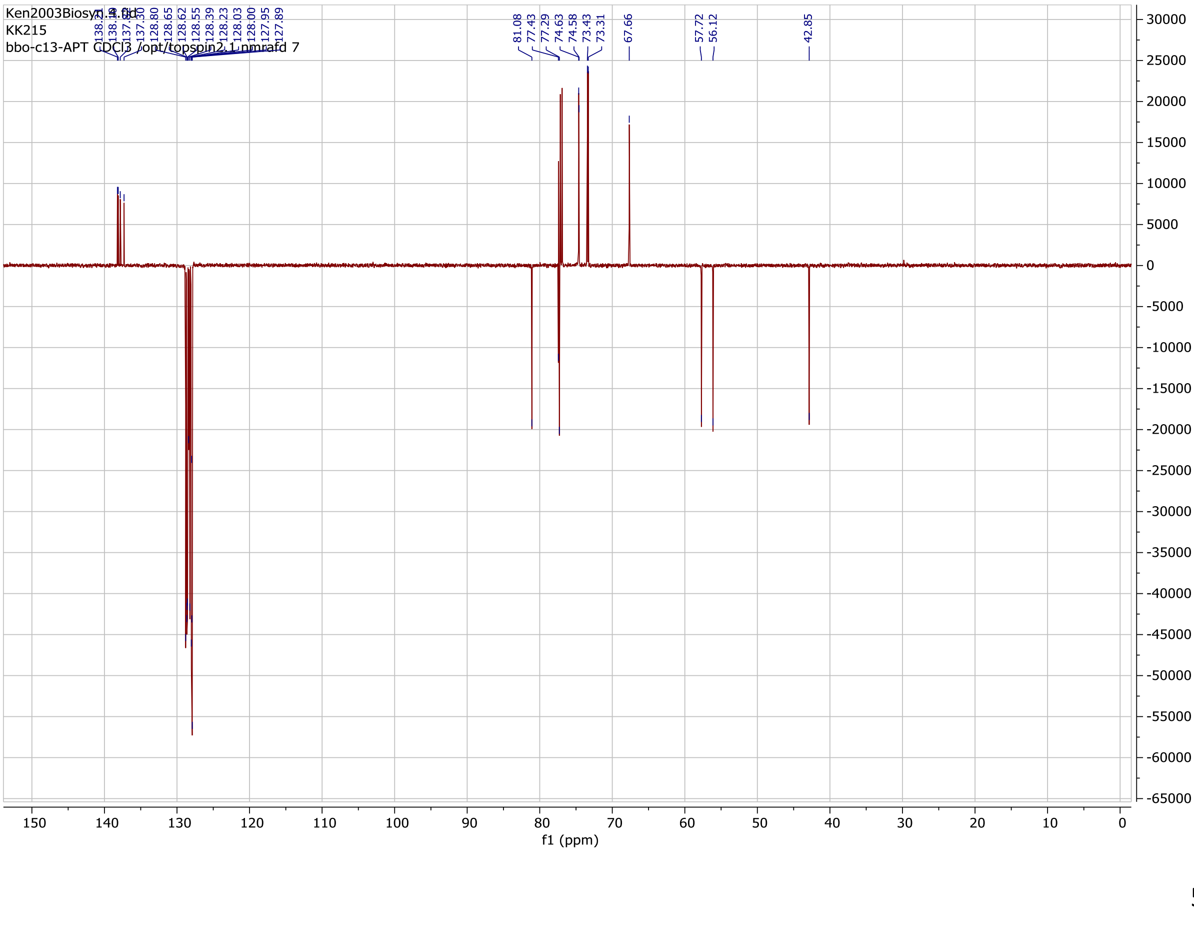

^{1}H-NMR and ^{13}C-NMR of **8** in MeOD:

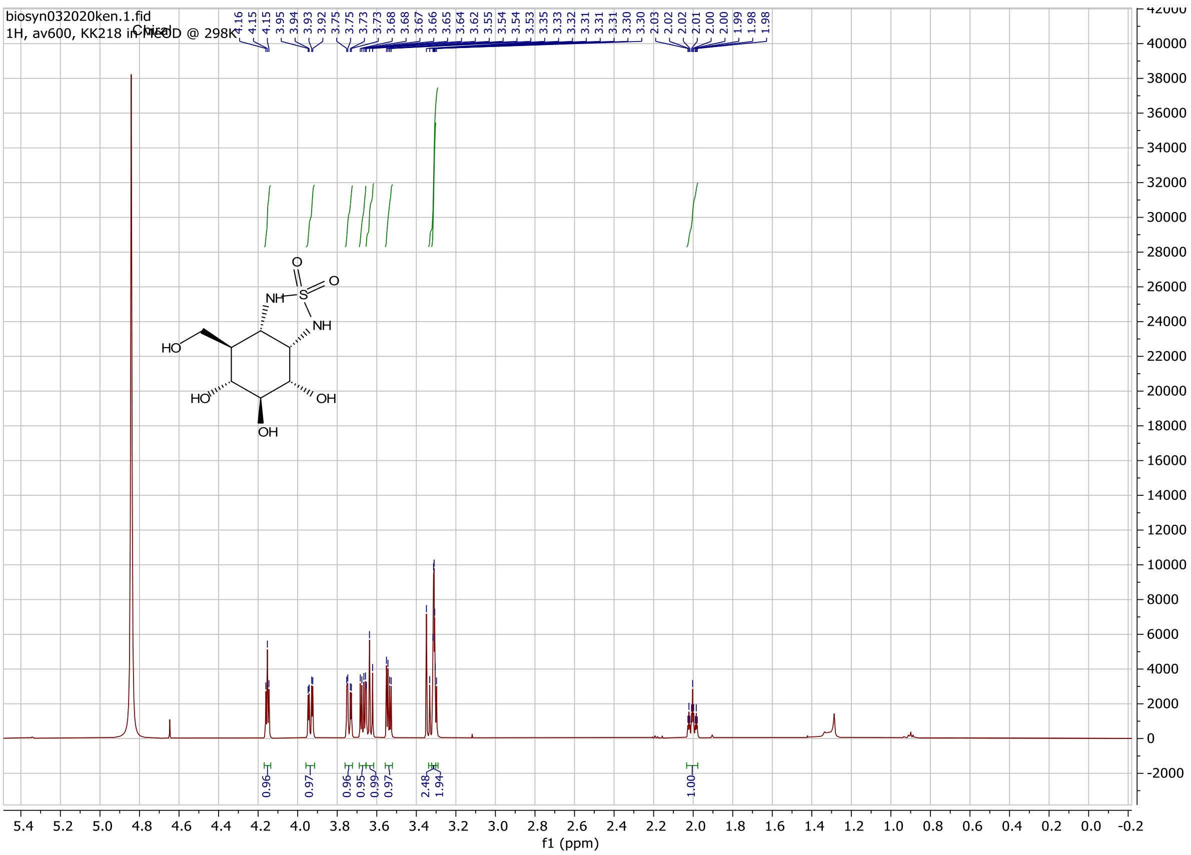

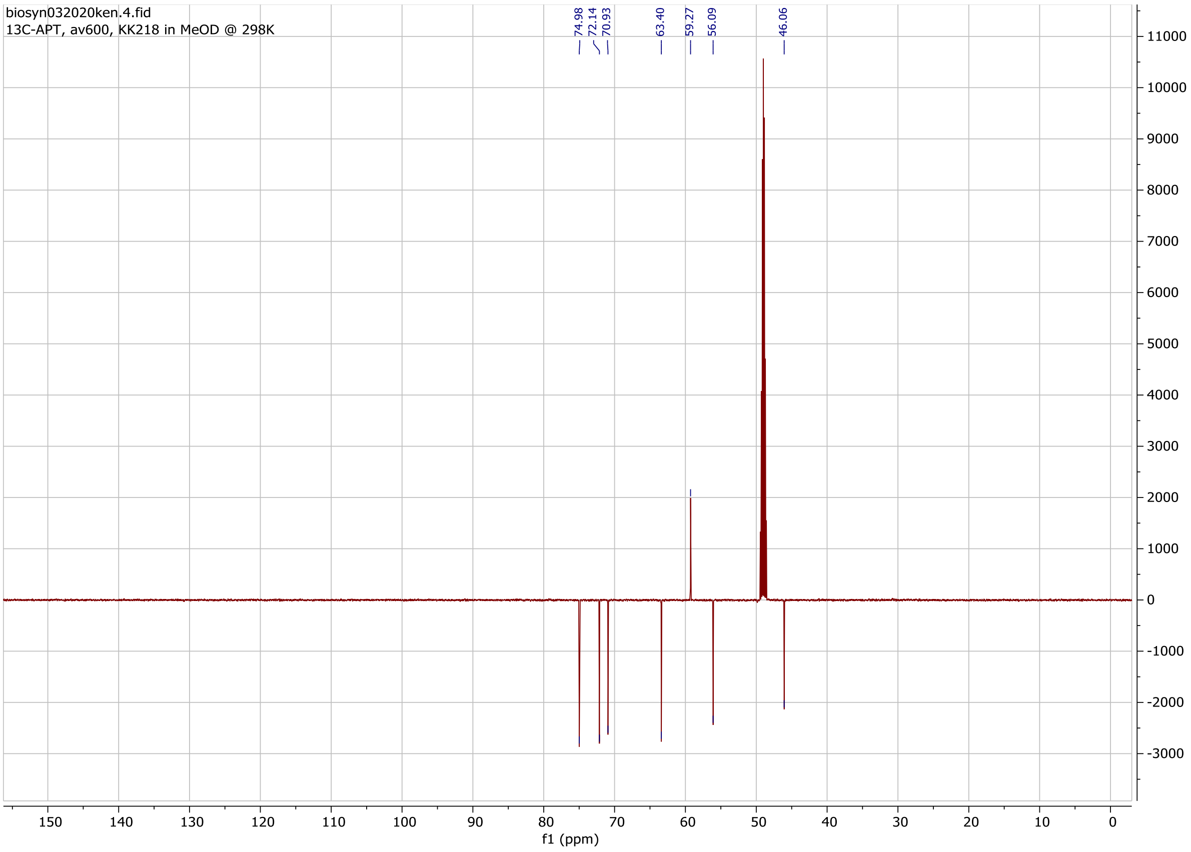

5. References

(1) Artola, M.; Wu, L.; Ferraz, M. J.; Kuo, C. L.; Raich, L.; Breen, I. Z.; Offen, W. A.; Codée, J. D. C.; Van Der Marel, G. A.; Rovira, C.; et al. 1,6-Cyclophellitol Cyclosulfates: A New Class of Irreversible Glycosidase Inhibitor. *ACS Cent. Sci.* **2017**, *3* (7), 784–793.

(2) Roig-Zamboni, V.; Cobucci-Ponzano, B.; Iacono, R.; Ferrara, M. C.; Germany, S.; Bourne, Y.; Parenti, G.; Moracci, M.; Sulzenbacher, G. Structure of Human Lysosomal Acid α-Glucosidase-A Guide for the Treatment of Pompe Disease. *Nat. Commun.* **2017**, *8*, 1111.

(3) Kabsch, W. XDS. *Acta Crystallogr. D. Biol. Crystallogr.* **2010**, *66*, 125–132.

(4) Winn, M. D.; Ballard, C. C.; Cowtan, K. D.; Dodson, E. J.; Emsley, P.; Evans, P. R.; Keegan, R. M.; Krissinel, E. B.; Leslie, A. G. W.; McCoy, A.; et al. Overview of the CCP4 Suite and Current Developments. *Acta Crystallographica Section D: Biological Crystallography*. **2011**, pp 235–242.

(5) Kovalevskiy, O.; Nicholls, R. A.; Long, F.; Carlon, A.; Murshudov, G. N. Overview of Refinement Procedures within REFMAC5: Utilizing Data from Different Sources. *Acta Crystallogr. Sect. D, Struct. Biol.* **2018**, *74* (Pt 3), 215–227.

(6) Lebedev, A. A.; Young, P.; Isupov, M. N.; Moroz, O. V.; Vagin, A. A.; Murshudov, G. N. JLigand: A Graphical Tool for the CCP4 Template-Restraint Library. *Acta Crystallogr. Sect. D Biol. Crystallogr.* **2012**, *68* (4), 431–440.

(7) Emsley, P.; Lohkamp, B.; Scott, W. G.; Cowtan, K. Features and Development of Coot. *Acta Crystallogr. D. Biol. Crystallogr.* **2010**, *66* (Pt 4), 486–501.

(8) Williams, C. J.; Headd, J. J.; Moriarty, N. W.; Prisant, M. G.; Videau, L. L.; Deis, L. N.; Verma, V.; Keedy, D. A.; Hintze, B. J.; Chen, V. B.; et al. MolProbity: More and Better Reference Data for Improved All-Atom Structure Validation. *Protein Sci.* **2018**, *27* (1), 293–315.

(9) Larsbrink, J.; Izumi, A.; Hemsworth, G. R.; Davies, G. J.; Brumer, H. Structural Enzymology of Cellvibrio Japonicus Agd31B Protein Reveals α-Transglucosylase Activity in Glycoside Hydrolase Family 31. *J. Biol. Chem.* **2012**, *287* (52), 43288–43299.

(10) Winter, G. Xia2: An Expert System for Macromolecular Crystallography Data Reduction. *J. Appl. Crystallogr.* **2010**, *43* (1), 186–190.

(11) Murshudov, G. N.; Skubák, P.; Lebedev, A. A.; Pannu, N. S.; Steiner, R. A.; Nicholls, R. A.; Winn, M. D.; Long, F.; Vagin, A. A. REFMAC5 for the Refinement of Macromolecular Crystal Structures. *Acta Crystallogr. Sect. D Biol. Crystallogr.* **2011**, No. D67, 355–367.

(12) Emsley, P.; Cowtan, K. Coot: Model-Building Tools for Molecular Graphics. *Acta Crystallogr. Sect. D Biol. Crystallogr.* **2004**, *60* (12 I), 2126–2132.

(13) Jiang, J.; Kuo, C. L.; Wu, L.; Franke, C.; Kallemeijn, W. W.; Florea, B. I.; Van Meel, E.; Van Der Marel, G. A.; Codée, J. D. C.; Boot, R. G.; et al. Detection of Active Mammalian GH31 α-Glucosidases in Health and Disease Using in-Class, Broad-Spectrum Activity-Based Probes. *ACS Cent. Sci.* **2016**, *2* (5), 351–358.

(14) Witte, M. D.; Kallemeijn, W. W.; Aten, J.; Li, K. Y.; Strijland, A.; Donker-Koopman, W. E.; van den Nieuwendijk, A. M. C. H.; Bleijlevens, B.; Kramer, G.; Florea, B. I.; et al. Ultrasensitive in Situ Visualization of Active Glucocerebrosidase Molecules. *Nat. Chem. Biol.* **2010**, *6* (12), 907–913.

(15) Davies, G. J.; Rowland, R. J.; Chen, Y.; Breen, I.; Wu, L.; Offen, W. A.; Beenakker, T.; Su, Q.; van den Nieuwendijk, A. M. C. H.; Aerts, J. M. F. G.; et al. Design, Synthesis and Structural Analysis of Glucocerebrosidase Imaging Agents. *Chem. – A Eur. J.* **2021**.

(16) Artola, M.; Kuo, C.-L.; Lelieveld, L. T.; Rowland, R. J.; Van Der Marel, G. A.; Codée, J. D. C.; Boot, R. G.; Davies, G. J.; Aerts, J. M. F. G.; Overkleeft, H. S. Functionalized Cyclophellitols Are Selective Glucocerebrosidase Inhibitors and Induce a Bona Fide Neuropathic Gaucher Model in Zebrafish. *J. Am. Chem. Soc.* **2019**, *141* (10), 4214–4218.

(17) Li, K. Y.; Jiang, J.; Witte, M. D.; Kallemeijn, W. W.; Van Den Elst, H.; Wong, C. S.; Chander, S. D.; Hoogendoorn, S.; Beenakker, T. J. M.; Codée, J. D. C.; et al. Synthesis of Cyclophellitol, Cyclophellitol

Aziridine, and Their Tagged Derivatives. *European J. Org. Chem.* 2014, *2014* (27), 6030–6043.

(18) Schröder, S. P.; Wu, L.; Artola, M.; Hansen, T.; Offen, W. A.; Ferraz, M. J.; Li, K.-Y.; Aerts, J. M. F. G.; Van Der Marel, G. A.; Codée, J. D. C.; et al. Gluco-1 H -Imidazole: A New Class of Azole-Type β-Glucosidase Inhibitor. *J. Am. Chem. Soc.* 2018, *140* (15).
